# Serum calcium and 25-hydroxyvitamin D in relation to longevity, cardiovascular disease and cancer: a Mendelian randomization study

**DOI:** 10.1038/s41525-021-00250-4

**Published:** 2021-10-14

**Authors:** Shuai Yuan, John A. Baron, Karl Michaëlsson, Susanna C. Larsson

**Affiliations:** 1 Unit of Cardiovascular and Nutritional Epidemiology, Institute of Environmental Medicine, Karolinska Institutet, Stockholm, Sweden; 2 Department of Epidemiology, Geisel School of Medicine at Dartmouth, Hanover, NH USA; 3 Department of Medicine, University of North Carolina School of Medicine, Chapel Hill, NC USA; 4 Department of Epidemiology, Gillings School of Global Public Health, University of North Carolina, Chapel Hill, NC USA; 5 Unit of Medical Epidemiology, Department of Surgical Sciences, Uppsala University, Uppsala, Sweden

**Keywords:** Predictive markers, Cardiovascular diseases, Cancer, Risk factors

## Abstract

Associations of serum calcium (S-Ca) and 25-hydroxyvitamin D (S-25(OH)D) concentrations with longevity, cardiovascular disease, and cancer are not clear. We conducted a Mendelian randomization study to examine the associations of S-Ca and S-25(OH)D with longevity and risk of cardiovascular disease and cancer. The primary genetic instruments for S-Ca and S-25(OH)D were obtained from genome-wide association meta-analyses that included 61,054 individuals for S-Ca and up to 79,366 individuals for S-25(OH)D. Genetic variants associated with S-Ca and S-25(OH)D in the UK Biobank were used as confirmatory instruments. We obtained summary-level data for associations of these instruments with individual survival later than the 90^th^ versus at most the 60^th^ percentile of expected age at death from a genome-wide association meta-analysis including 11,262 cases and 25,483 controls, and with parental longevity (both parents in top 10% percentile) from the UK Biobank including 7,182 cases and 79,767 controls. Data for cardiovascular disease (111,108 cases and 107,684 controls) and cancer (38,036 cases and 180,756 controls) were obtained from the FinnGen consortium. A one standard deviation increase in genetically-predicted S-Ca concentration was associated with lower odds of longevity (odds ratio, 0.72; 95% CI, 0.55-0.95) and increased risk of cardiovascular disease (odds ratio, 1.11; 95% CI, 1.03-1.20). The associations were consistent in confirmatory analyses. There was no evidence supporting an association between genetically-predicted S-Ca and cancer, and no associations of genetically-predicted S-25(OH)D with the studied outcomes. Lifelong higher levels of S-Ca but not S-25(OH)D may shorten life expectancy and increase the risk of cardiovascular disease.

## Introduction

Calcium and vitamin D supplements are frequently used for the prevention and treatment of osteoporosis despite weak and inconsistent evidence that they prevent fractures in community-dwelling women and men^1–4^. Several other benefits and adverse effects of calcium^5^ and vitamin D^6,7^ have been suggested. Epidemiological evidence regarding the associations of these nutrients with all-cause and cardiovascular disease mortality is inconsistent and data on longevity are scarce^8–14^. The net benefit on health by calcium and vitamin D supplement use is accordingly unclear.

Supplementation with calcium and vitamin D, alone or together, leads to increases in serum calcium (S-Ca) concentration with a peak 4 h after each ingestion and a more long-lasting elevation in serum 25-hydroxyvitamin D (S-25(OH)D), the marker metabolite for vitamin D status^15–18^. Whether regular calcium supplementation elevates S-Ca after several months of use is debatable^19^ but this question has great importance for the understanding of the effects of calcium supplementation and S-Ca on health outcomes since continued use for many years is recommended.

The Mendelian randomization (MR) design can overcome residual confounding and other biases in epidemiological studies, thereby strengthening causal inference for an exposure–outcome association by leveraging genetic variants as instrumental variables for an exposure^20^. In this study, we first explored the long-term effects of calcium and calcium plus vitamin D supplementation on S-Ca concentrations in a meta-analysis. Then we used the MR design to assess the associations of genetically predicted lifelong small increases of S-Ca and S-25(OH)D concentrations with longevity as well as with risk of overall cardiovascular disease and cancer.

## Results

### Meta-analysis of the effect of calcium supplementation on S-Ca

There were 7 studies (473 participants with calcium supplements of 500–1600 mg/day and 464 controls) included in the meta-analysis of calcium supplementation and 7 studies (400 participants with calcium supplements of 500–1200 mg/day and vitamin D supplementation of 500–1000 IU/day and 372 controls) included in the meta-analysis of calcium plus vitamin D supplementation. The interventions lasted from 6 months to 4 years. S-Ca concentrations were significantly higher in individuals who had been assigned supplementation with calcium alone or calcium plus vitamin D compared with those given placebo (Supplementary Figs. 1 and 2). The differences in S-Ca were 0.03 mmol/L (95% confidence interval (CI), 0.02, 0.04) and 0.04 mmol/L (95% CI, 0.02, 0.05) for individuals given calcium and calcium plus vitamin D supplements, respectively.

The *I*^2^ statistics were 64.8 and 52.6% for the meta-analysis of calcium supplementation alone and that for calcium plus vitamin D, respectively, indicating moderate heterogeneity across studies in differences in S-Ca after supplementation. However, similar differences were also seen in the random-effects model. Both the direction and magnitude of associations remained in the sensitivity analyses confined to studies reporting fasting post-treatment S-Ca (Supplementary Figs. 3 and 4). We found no evidence of small study bias in the two meta-analyses. The *p* values of the Egger’s tests were 0.599 and 0.902 for calcium supplementation and calcium plus vitamin D supplementation, respectively. The funnel plots are displayed in Supplementary Fig. 5 and we detected a symmetry distribution of dots and no possible outliers.

### MR analyses of S-Ca and S-25(OH)D in relation to longevity

**Fig. 1:**
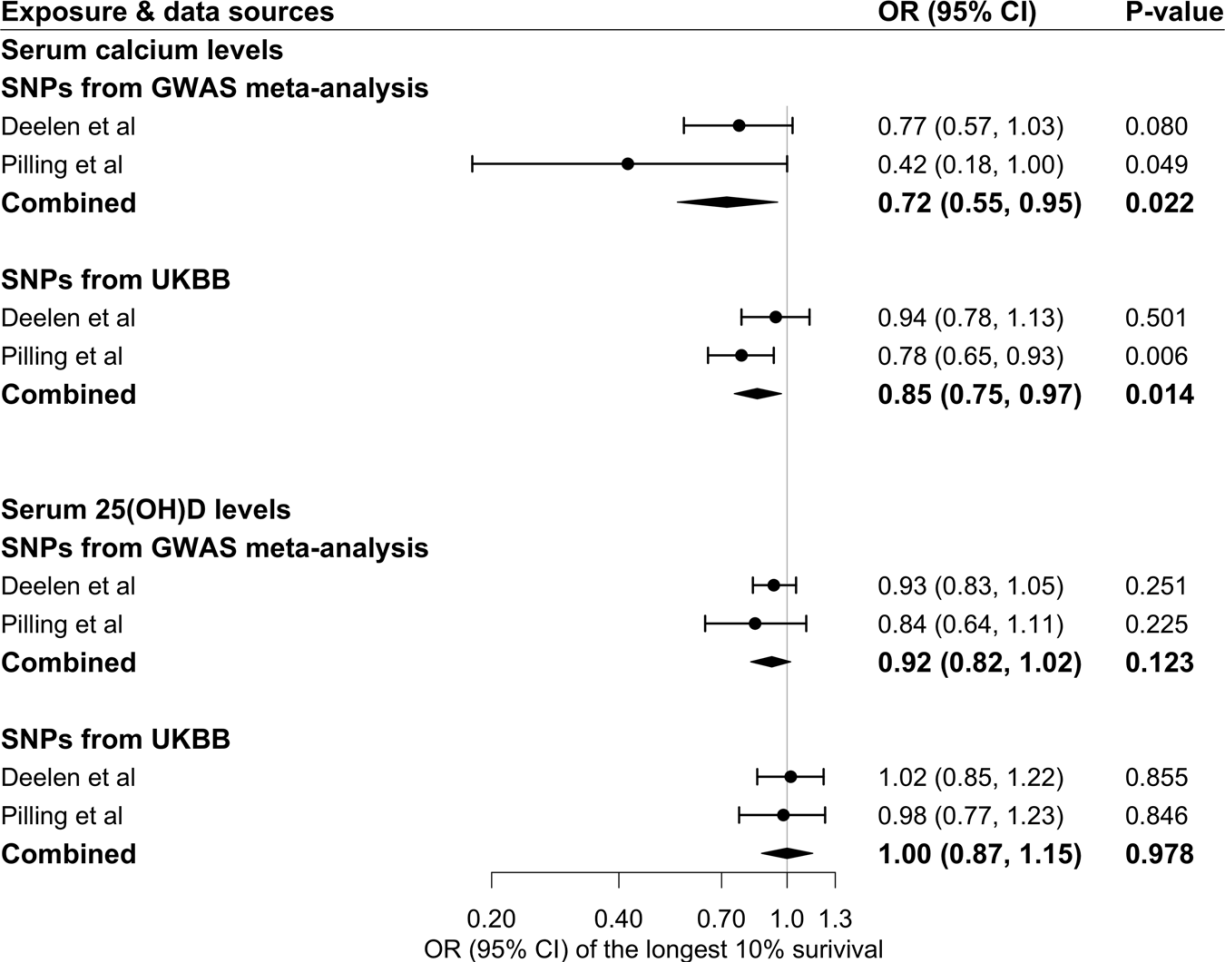
Associations of genetically predicted serum calcium and 25(OH)D concentrations with longevity in two independent study samples. 25(OH)D 25-hydroxyvitamin D, CI confidence interval, GWAS genome-wide association study, OR odds ratio, SNP single-nucleotide polymorphism, UKBB UK Biobank. The estimates of MR analyses were derived from inverse-weighted model with random effects and scaled to one standard deviation increase in serum calcium and 25(OH)D concentrations. The cases were defined by living to an age beyond the 90th survival percentile based on individual cohort life tables from census data from the appropriate country, sex, and birth cohort and the controls were individuals who died at or before the 60th percentile of the expected age at death or whose age at the last follow-up visit was ≤60th survival percentile in Deelen et al. study. The cases were individuals with both parents’ lifespan in top 10% (father’s age ≥86 years and mother’s age ≥90 years) in Pilling et al. study.

Genetically predicted lifelong higher S-Ca was associated with lower odds of longevity, an association that was consistent in the two data sources (Fig. 1). For one standard deviation (SD) increase in the predicted S-Ca, the combined odds ratio (OR) for survival beyond the 90th percentile was 0.72 (95% CI, 0.55, 0.95). The association remained consistent in confirmatory analysis using instruments from the UK Biobank albeit with attenuated magnitude (corresponding OR 0.85; 95% CI, 0.75, 0.97) (Fig. 1) and in the supplementary analysis based on data from a genome-wide association study (GWAS) meta-analysis of the UK Biobank and LifeGen (Supplementary Table 3). In contrast, genetically predicted S-25(OH)D was not associated with longevity (Fig. 1). These findings for the associations of S-Ca and S-25(OH)D with longevity were consistent across all sensitivity analyses (Supplementary Table 4). No likely heterogeneity among single-nucleotide polymorphisms (SNPs) and pleiotropy (*p* values for the intercept of MR-Egger >0.05) was observed in the primary analyses for S-Ca and S-25(OH)D.

### MR analyses of S-Ca and S-25(OH)D in relation to cardiovascular disease and cancer

**Fig. 2:**
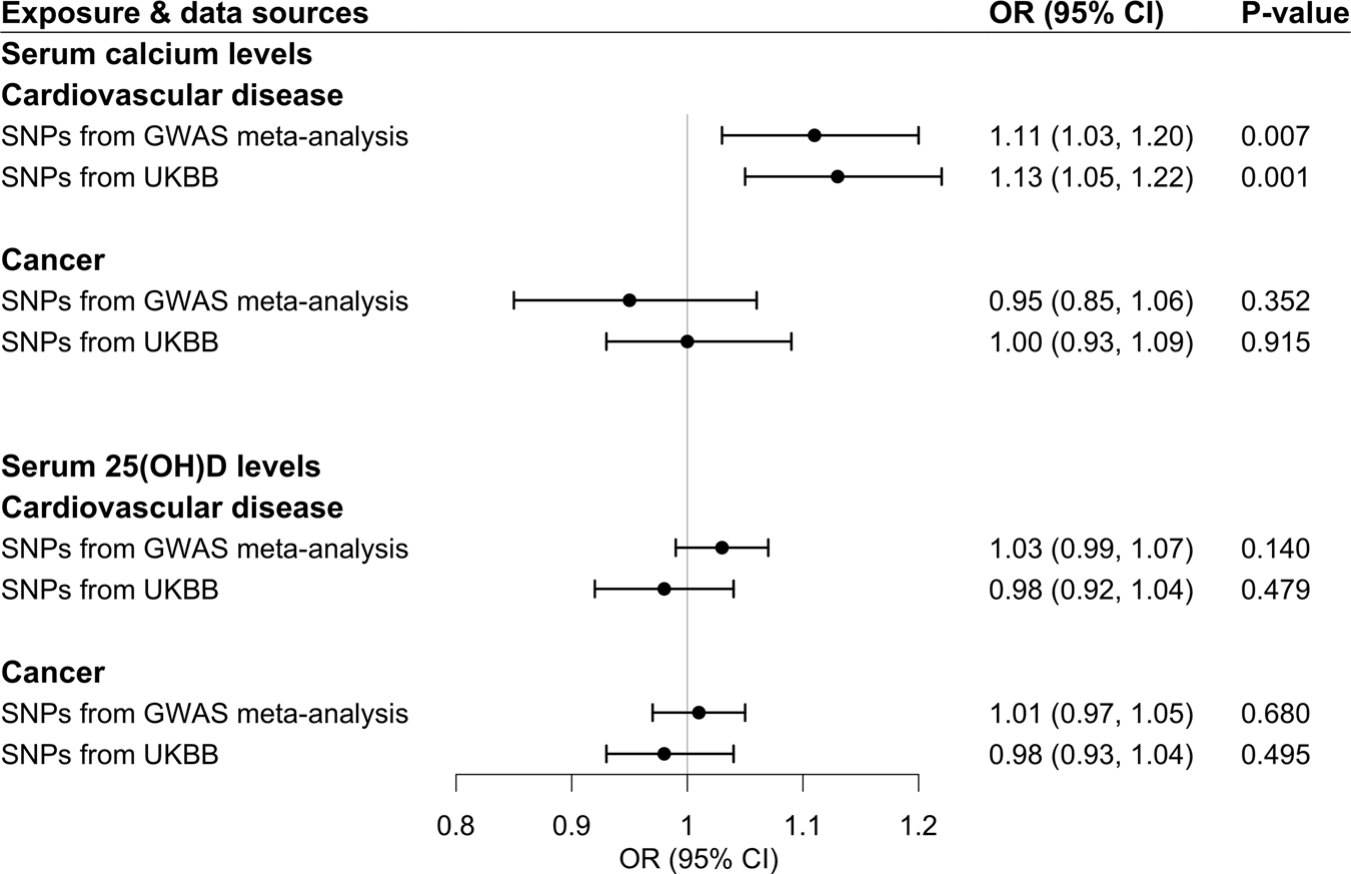
Associations of genetically predicted serum calcium and 25(OH)D concentrations with cardiovascular disease and cancer in the FinnGen consortium. 25(OH)D 25-hydroxyvitamin D, CI confidence interval, CVD cardiovascular disease, GWAS genome-wide association study, OR odds ratio, SNP single-nucleotide polymorphism, UKBB UK Biobank. The estimates of MR analyses were derived from inverse-weighted model with random effects and scaled to one standard deviation increase in serum calcium and 25(OH)D concentrations.

Associations of genetically predicted S-Ca and S-25(OH)D concentrations with cardiovascular disease and cancer are displayed in Fig. 2. Genetic predisposition to higher S-Ca was associated with an increased risk of cardiovascular disease, with an OR per SD increase in predicted S-Ca of 1.11 (95% CI, 1.03, 1.20) in the analysis based on the primary genetic instrument and 1.13 (95% CI, 1.05, 1.22) in the analysis using the confirmatory genetic instrument (Fig. 2). There was no association of genetically predicted S-Ca with cancer or of genetically predicted S-25(OH)D with either cardiovascular disease or cancer (Fig. 2). The positive association between predicted S-Ca and cardiovascular disease remained in the weighted median analysis (Supplementary Table 5).

## Discussion

Our meta-analysis indicated that calcium and calcium plus vitamin D supplementation increased fasting S-Ca concentrations over 1–4 years of use. MR analyses showed that genetically predicted lifelong increases of S-Ca concentrations were associated with lower odds of longevity and with an increased risk of cardiovascular disease. There was no evidence supporting associations of S-Ca with cancer and of S-25(OH)D with longevity, cardiovascular disease, or cancer.

Calcium supplementation (1000 mg dose) leads to an acute elevation of S-Ca with a peak of about 0.09 mmol/L increase after 4 h^17^. S-Ca is homeostatically regulated and the effect of prolonged calcium supplementation on fasting S-Ca has not previously been comprehensively assessed. Here we conducted a meta-analysis to address this question and found that both calcium and calcium plus vitamin D supplementation led to increased fasting S-Ca after several years of supplement use. Mean fasting S-Ca was observed to be higher (~0.04 mmol/L, about a half SD change on the population level) in the group assigned calcium and vitamin D supplements than in the placebo group. This change in S-Ca corresponds well with the previously published short-term change in S-Ca measured 12 h after a dose of calcium carbonate and calcium citrate^21^, the two compounds most widely used for supplementation^21^. This change in S-Ca might imply a substantial shift in the proportion of individuals with very high concentrations. Hence, the moderate nadir mean change could have a considerable impact in high-risk individuals.

Our findings linking genetically higher S-Ca concentrations to lower life expectancy are consistent with some, but not all, traditional observational studies on calcium intake or circulating calcium in relation to mortality. A meta-analysis of eight cohort studies showed a 13% increased risk of all-cause mortality per one SD increase in S-Ca concentrations; however, this finding might be affected by confounding^11^. Similarly, a cohort study of 61,433 Swedish women with 19 years of follow-up found that women with a total calcium intake >1400 mg/day versus 600–1000 mg/day had a 40% increased risk of death from all causes and a 49% higher cardiovascular mortality^22^. In contrast, other observational studies whose participants had on average lower calcium intake found inverse associations of dietary calcium with death from all causes^8,10,23^ and cardiovascular disease^8,23^. With regard to supplemental calcium intake, two large-scale studies in older women had divergent conclusions that supported^24^ and opposed^22^ calcium supplement use in prolonging longevity. These inconsistent findings may potentially be related to residual confounding in the observational studies. The National Osteoporosis Foundation and American Society for Preventive Cardiology developed a guideline that total calcium intake <2500 mg/day should be considered safe from a standpoint of cardiovascular disease and cardiovascular and all-cause mortality^25^. Nonetheless, it was noted that the risk of death was 157% higher for individuals with daily calcium supplementation (≥500 mg) versus no treatment among women with a dietary calcium intake >1400 mg/day^22^. Our estimates are not directly comparable to those from previous studies as our results were scaled per SD increment of S-Ca, whereas the change in long-term S-Ca from high dietary or supplemental calcium intake may be smaller, as suggested by our meta-analysis of intervention studies of calcium supplementation.

Our study further showed a possible causal association between elevated S-Ca and increased risk of cardiovascular disease, which may explain the observed association between S-Ca and longevity. A meta-analysis of 8 studies^11^ and a subsequent cohort study including 441,738 participants of the AMORIS database also showed a positive association between circulating calcium concentrations and risk of cardiovascular disease^26^. This association was confirmed in a large-scale study that included 16,718 women and its incorporation into a meta-analysis with 8 other studies on calcium supplements and cardiovascular events^27^. Two recent systematic reviews, however, suggested no effect of calcium supplements on cardiovascular disease risk^9,25^. With regard to individual cardiovascular diseases, genetically predicted S-Ca was associated with coronary artery disease and myocardial infarction^28^ but not with ischemic stroke^29^, heart failure^30^, or atrial fibrillation^31^ in previous MR studies. The elevated S-Ca may exert effects on vascular calcification, endothelial function, blood coagulation, and altered gene expression induced by signaling through the calcium-sensing receptor expressed in the endothelium and vascular smooth muscle cells^32^, thereby increasing the risk of cardiovascular disease. Calcium intake might be associated with certain specific cancers^33–35^, but data on the association of S-Ca with overall cancer risk are limited^36^.

Our study shows that the levels of S-Ca increase with dietary calcium supplement use. Menopause-related calcium loss from bone and osteoporotic calcium loss are also important in raising S-Ca levels^37,38^. In addition, physical inactivity has been shown to increase S-Ca^39^, so an indirect detrimental effects of calcium loss from bone during immobilization could be a decrease in longevity and increase in risk of cardiovascular disease.

Findings on the associations of vitamin D with mortality and risk of cardiovascular disease are conflicting. Most observational studies have reported an inverse association of circulating S-25(OH)D concentrations with mortality^40^ and cardiovascular disease^41^. Nonetheless, evidence from randomized controlled trials does not confirm a reduced risk of all-cause mortality or cardiovascular disease with vitamin D supplementation^13,14,42^. Our MR findings confirm and extend the findings of the trials by showing that increased S-25(OH) concentrations are not associated with longevity or cardiovascular disease in the general population. Our study found no association between S-25(OH)D and cancer risk, in agreement with results of a meta-analysis of randomized controlled trials showing that vitamin D supplementation reduced total cancer mortality but not total cancer incidence^43^. Likewise, a recent large-scale randomized controlled trial revealed that vitamin D supplementation did not result in a lower incidence of invasive cancer^42^. A small proportion (~20%) of the population in the UK Biobank had S-25(OH)D levels in the deficiency range in the UK Biobank. Although no potential adverse effects of vitamin D supplementation have been reported in previous studies, the risk of hypercalcemia appears to increase with large supplemental doses of vitamin D taken for ≥1 year^44^. Given the potentially different effects of bioavailable and total 25(OH)D concentrations^45–47^ on disease and mortality, additional large clinical studies are warranted to disentangle these complex associations and potential threshold effects in both fair- and dark-skinned individuals.

There are three important assumptions of MR studies. The first is that the genetic instruments should be robustly associated with the exposure. In our study, we selected SNPs associated with S-Ca and S-25(OH)D at the genome-wide significance level. For SNPs with linkage disequilibrium, the SNP with the lowest *p* value for the genome-wide association was retained. In addition, we calculated the *F*-statistic for all sets of instruments to assess the strength of the instrumental variables. These were all >10, indicating good strength of the genetic instruments we used. However, whether these SNPs reflect the levels of S-Ca and S-25(OH)D in the outcome populations could not be examined. The second assumption is that the SNPs used as instruments for the exposure (i.e., S-Ca and S-25(OH)D in this study) should not be associated with important confounders in the study population. Because of our reliance on summary-level genetic data, we were unable to examine this issue. The third assumption is that instruments in an MR study should influence the risk of outcomes via the exposure only, not via alternative pathways (i.e., absence of pleiotropy). We assessed this assumption using MR-Egger analysis and did not observe any indication of pleiotropy, except for S-25(OH)D proxied by SNPs from the UK Biobank in relation to longevity in the data from the GWAS conducted by Pilling et al. The associations also remained consistent in the MR-PRESSO analysis after removal of potential outliers. Nonetheless, we cannot completely rule out the possibility that observed associations were influenced by horizontal pleiotropy.

There are several strengths of the present study. We examined the associations of lifelong higher S-Ca and S-25(OH)D with longevity in two independent outcome data sources with up to total of 123,694 individuals. The consistency of the results in primary and confirmatory analyses solidified the causal inference on the detrimental effect of high S-Ca on longevity. Although our analyses were confined to European populations, population structure was adjusted for in GWASs for exposures and outcomes. Thus, it is unlikely that population stratification bias affected our results, but we cannot generalize our findings to non-European populations with potentially different mean S-Ca and S-25(OH)D concentrations. We used summary-level data from large genetic consortia in combination with the UK Biobank study, thereby assuring high statistical power to detect weak associations.

Limitations of this summary-level MR study are that we could not assess possible non-linear associations and interaction effects of S-Ca and S-25(OH)D with other modifiable factors (e.g., smoking, healthy dietary patterns, alcohol consumption, and obesity). Another limitation is that, in the longevity analysis, the inclusion of individuals lost to follow-up before the 60th percentile of age at death in controls introduces some misclassification since these individuals could have survived beyond the 60th percentile and potentially even beyond the age corresponding to the 90th survival percentile. However, the misclassification in controls was estimated to be small^48^. Thus, this bias is unlikely to be substantial. We detected an attenuated association between genetically predicted S-Ca levels and longevity in the supplementary analysis using SNPs from UK Biobank, which might reflect the compromised validity of certain instruments.

Longevity of subjects and parental lifespan are different, though related phenotypes. Nevertheless, there is strong genetic correlation between the two^48^, so the UK Biobank data provide useful confirmation of the findings in our main longevity analysis. Parental longevity similarly confirmed a GWAS of longevity in a previous study^48^. Of course, this is not surprising: parents share half of their genetic code (including longevity-associated genes) as well as possibly healthy lifestyles with their offspring.

In summary, genetically proxied lifelong higher S-Ca but not S-25(OH)D was associated with an increased risk of cardiovascular disease and lower odds of longevity. These adverse impacts of high S-Ca concentrations may dominate over the positive effects since current evidence does not support the general use of such supplements for the prevention of fractures in healthy community-dwelling adults.

## Methods

### Meta-analysis of the effects of calcium supplementation on S-Ca

Studies included in the present meta-analysis were identified from a published meta-analysis assessing the effect of calcium and calcium plus vitamin D supplementations on bone mineral density. That meta-analysis included 32 trials of calcium supplementation and 19 trials of supplementation with calcium plus vitamin D supplementation^49^. We reviewed all included studies and extracted demographic features, dose and duration of supplementation, and post-treatment S-Ca information from all studies that reported S-Ca after intervention. Detailed methods are presented in Supplementary Methods 1.

### MR study design

The present MR study was based on summary-level data from published and publicly available GWASs regarding S-Ca and S-25(OH)D, longevity, cardiovascular disease, and cancer. Detailed information on the included studies and consortia is presented in Supplementary Table 1. We first assessed the associations of genetically proxied S-Ca and S-25(OH)D with longevity in two independent data sources. Subsequently, to understand the underlying mechanisms of any associations of S-Ca and S-25(OH)D with longevity, further MR analyses were conducted to assess the associations of genetically predicted S-Ca and S-25(OH)D with cardiovascular disease and cancer. The used genetic studies have had been permitted by corresponding ethical committees. All participants have had provided inform consent. The present analyses, based on summary-level data, have been approved by the Swedish Ethical Review Authority.

### Instrument selection

A meta-analysis of 28 GWASs of S-Ca that included up to 61,054 participants of European ancestry identified 7 independent (*r*^2^ < 0.01) SNPs associated with S-Ca at the genome-wide significant level (*p* < 5 × 10^−8^)^50^. Those SNPs were used as the primary set of genetic instruments for S-Ca in this study. A confirmatory set of 198 SNPs associated with S-Ca at *p* < 5 × 10^−8^ was obtained from the UK Biobank study that included 315,153 participants of European ancestry^51^. Detailed description of the instrument selection procedure for S-Ca in the UK Biobank is described in Supplementary Methods 2. Similarly, we used seven SNPs associated with S-25(OH)D from meta-analyses of GWASs^52,53^. One of these SNPs, rs117913124 with low frequency but a large effect on S-25(OH)D, was identified from a sample of 42,274 participants of European ancestry^52^, and the other six SNPs were selected from 31 cohorts with 79,366 participants of European ancestry^53^. We also used 143 SNPs as confirmatory instruments for S-25(OH)D from a recent study with 417,580 participants of European ancestry in the UK Biobank^54^. Detailed information on genetic instruments for S-Ca and S-25(OH)D is displayed in Table 1.

**Table 1:** Genetic instruments for serum calcium and 25(OH)D concentrations.

Exposure	Data source	SNPs	VE%	*F*-statistic^a^	Mean concentration^b^
Calcium	Meta-analysis of 28 GWASs	7	0.9	47/111	2.4 mmol/L
Calcium	UK Biobank	198	10.5	17/41	2.4 mmol/L
25(OH)D	Meta-analysis of 31 and 17 GWASs	7	5.3	263/623	NA
25(OH)D	UK Biobank	143	7.5	18/42	49.6 nmol/L

*25(OH)D* 25-hydroxyvitamin D, *GWAS* genome-wide association study, *NA* not available, *VE* variance explained, *SNP* single-nucleotide polymorphism.

^a^*F*-statistic was calculated based on the variance explained by used SNPs in the exposure GWAS and the sample size of GWAS by Deelen et al. or Pilling et al.

^b^The between-participant SDs for calcium concentrations in the meta-analysis of GWAS and UK Biobank were 0.12 and ~0.09 mmol/L, respectively. The SD for 25(OH)D concentrations in the UK Biobank was ~21.14 nmol/L.

### Outcome sources

Summary-level data for longevity were available from a discovery meta-analysis of 18 GWAS cohorts of longevity. Long-lived cases were 11,262 European-descent participants who lived to an age ≥90th survival percentile based on individual cohort life tables from census data from each country, by sex and birth cohort. Early mortality controls were 25,483 European descent individuals who died at or before the 60th percentile of the expected age at death or whose age at the last follow-up visit was ≤60th survival percentile^48^. In one included study, the 60th and 90th survival percentile corresponded, respectively, to age 75 and 89 years for men and 83 and 94 years for women. Since many of the included studies comprised individuals who were relatively old at baseline, the number of controls was less than the number of cases in some studies^48^. We also used summary-level data on longevity potential from the UK Biobank with 7182 cases and 79,767 controls^55^, which was not included in either discovery or replication stages of the previous GWAS meta-analysis described above^48^. Given that participants in UK Biobank were generally young, cases were defined by requiring both parents’ lifespan in the top 10% of longevity (father’s age ≥86 years and mother’s age ≥90 years). This phenotype, though conceptually different from longevity, has a strong genetic correlation with longevity^48^. In supplementary analyses, we examined the associations of S-Ca and S-25(OH)D with lifespan using data from a GWAS meta-analysis on parental lifespan in UK Biobank and LifeGen including over 1 million individuals^56^. Summary-level data for cardiovascular disease (111,108 cases and 107,684 controls) and cancer (38,036 cases and 180,756 controls) were obtained from the fifth wave of analyses of the FinnGen consortium^57^. Detailed data on involved cohorts, genotypes, endpoint definition, and association test in the FinnGen consortium are available on the FinnGen webpage. Diagnostic information for cardiovascular disease and cancer is presented in Supplementary Table 2.

### Statistical analysis

Both fixed- and random-effects inverse-variance weighted models were used to estimate the difference in means of S-Ca between arms with and without calcium or calcium plus vitamin D supplementation from the included studies. The *I*^2^ statistic was calculated to present heterogeneity among estimates from different studies. Funnel plot and Egger’s test were used to evaluate small study bias and publication bias. The meta-analysis was performed using “metan” package in Stata/SE 15.0.

The random-effects inverse-variance weighted approach was employed in the primary statistical analysis in MR analysis^58^. Estimates for S-Ca and S-25(OH)D in relation to longevity from the two data sources were combined using fixed-effects meta-analysis. Several sensitivity analyses with different assumptions and strengths, including the weighted median^59^, MR-Egger regression^60^, and MR-PRESSO^61^ methods, were used to examine the consistency of results and detect and correct for possible pleiotropy. Assuming ≥50% weight from valid instruments, the weighted median method can provide consistent MR estimates^59^. MR-Egger regression can detect pleiotropic effects and provide estimate after correction of pleiotropy, although it compromises statistical power^60^. The MR-PRESSO model can detect possible outliers and generate estimates after removal of outliers, thereby correcting for horizontal pleiotropy^61^. Cochrane’s *Q* was calculated in the inverse-variance weighted models to assess heterogeneity among estimates of used SNPs and the *p* value for the intercept in MR-Egger regression^60^ was used to examine possible pleiotropy (*p* < 0.05). ORs and the corresponding CIs were scaled to one between-person SD increase in genetically predicted S-Ca (equal to 0.09–0.12 mmol/L and S-Ca peak difference with supplementation by 1000 mg calcium carbonate or citrate after up to 3 months of use^17^) and S-25(OH)D concentrations. All analyses were performed using the mrrobust package^62^ in Stata/SE 15.0 and the TwoSampleMR package^63^ in R Software 3.6.0.

## Supplementary information

Supplementary Information

## Data Availability

The present study was based on publicly available summary-level genetic data. All used data are available in Supplementary Tables 6 and 7. All used data are available upon a reasonable request to the corresponding author. The Neal Lab data on UK Biobank can be obtained via http://www.nealelab.is/uk-biobank. The summary-level data for Deelen et al. GWAS on longevity can be obtained via www.longevitygenomics.org/downloads. The summary-level data for Piling et al. GWAS on longevity can be found in MR-Base platform (http://app.mrbase.org/) by searching ebi-a-GCST003392. The summary-level data of GWAS in UK Biobank and LifeGen can be obtained via 10.7488/ds/2463.

## Code availability

Codes used in this study can be obtained upon a reasonable request to the corresponding author.
