# Supplementary material for: Serum calcium and 25-hydroxyvitamin D in relation to longevity, cardiovascular disease and cancer: a Mendelian randomization study
Source: NPJ Genom Med. 2021 Oct 14;6:86. doi: 10.1038/s41525-021-00250-4 (PMC8516873; doi:10.1038/s41525-021-00250-4)
Supplement: Supplementary file 1 — Supplementary Information [file 41525_2021_250_MOESM1_ESM.pdf]

Supplementary materials

Serum Calcium and 25-Hydroxyvitamin D in Relation to Longevity, Cardiovascular Disease and Cancer: A Mendelian Randomization Study

Shuai Yuan, John A. Baron, Karl Michaëlsson, Susanna C. Larsson

Supplementary Methods 1: Meta-analysis of the long-term effects of calcium and calcium plus vitamin D supplementation on S-Ca
Supplementary Methods 2: Instrument selection for S-Ca in UK Biobank

Supplementary Figure 1. Long-term effect of calcium supplementation on S-Ca concentrations
Supplementary Figure 2. Long-term effect of calcium plus vitamin D supplementation on S-Ca concentrations
Supplementary Figure 3. Long-term effect of calcium supplementation on fasting S-Ca concentrations
Supplementary Figure 4. Long-term effect of calcium plus vitamin D supplementation on fasting S-Ca concentrations
Supplementary Figure 5. Funnel plots for two meta-analyses

Supplementary Table 1. Used studies and consortia in the present study
Supplementary Table 2. Diagnostic information for cardiovascular disease and cancer in FinnGen
Supplementary Table 3. Associations of genetically predicted serum calcium and 25(OH)D with parent lifespans
Supplementary Table 4. Associations of genetically predicted serum calcium and 25(OH)D with longevity in sensitivity analyses
Supplementary Table 5. Associations of genetically predicted serum calcium and 25(OH)D with cardiovascular disease and cancer in sensitivity analyses
Supplementary Table 6. Data for analyses of serum calcium
Supplementary Table 7. Data for analyses of serum 25(OH)D

Supplementary Methods 1: Meta-analysis of long-term effects of calcium and calcium plus vitamin D supplementations on S-Ca

Meta-analysis procedure

Studies included in the meta-analysis those in a published meta-analysis assessing calcium and calcium plus vitamin D supplementations in relation to bone mineral density (Tai V et al, 2015, BMJ). That meta-analysis included 32 clinical trials of calcium supplementation of which seven reported data on S-Ca concentrations after the calcium supplement intervention and were included here. The meta-analysis also included 19 trials of supplementation with calcium plus vitamin D. Of these, seven provided data for total S-Ca and were included in the present meta-analysis. In sensitivity analyses of calcium supplements alone, we excluded the Recker et al, 1977 study (as participants were restricted to a low-calcium diet) and the Ruml et al, 1999 study that provided no information about fasting status. In the analysis of calcium plus vitamin D supplementation we excluded the studies by Baeksgaard et al, 1998, Doetsch et al, 2004 and Harwood et al, 2004 since they did not indicate that bloods were obtained fasting, the Meier et al, 2004 study since bloods were not obtained fasting post-treatment, and Chapuy et al, 2002 since participants with osteomalacia were included.

Statistical analysis: Both fixed and random effects inverse variance weighted models were used to estimate the mean difference in means of S-Ca from included studies. We manually converted reported S-Ca data in other units to mmol/L across studies and aimed to estimate a pooled mean difference in absolute concentrations, rather than a standardized difference. The I^2 statistic was calculated to assess heterogeneity among

estimates from different studies. The funnel plot and egger test were used to assess publication bias. The meta-analysis was performed using "metan" package in Stata/SE 15.0.

Table 1. Included studies in the meta-analysis of long-term effects of calcium supplementation on S-Ca concentrations

No	Study	Setting	Mean age	N-treatment	N-control	Calcium dose (mg/d)	Duration	Fasting blood	Diet	Treatment[a]		Placebo[a]	
										Mean	SD	Mean	SD
1	Recker et al, 1977	Community	57	14	12	1040	2 y	Yes	Low calcium	2.40	0.16	2.35	0.62
2	Lau et al, 1992	Institution	76	12	12	800	10 mon	Yes	Normal	2.33	0.04	2.18	0.16
3	Riggs et al, 1998	Community	66	88	89	1600	4 y	Yes	Normal	2.40	0.10	2.38	0.06
4	Ruml et al, 1999	Community	52	17	28	800	2 y	NA	Normal	2.40	0.08	2.43	0.08
5	Son et al, 2001	Community	72	22	21	1000	10 mon	Yes	Normal	2.35	0.35	2.24	0.43
6	Rajatanavin et al, 2010	Community	66	178	165	500	2 y	Yes	Normal	2.46	0.10	2.41	0.10
7	Nakamura et al, 2012	Community	60	142	137	500	2 y	Yes	Normal	2.29	0.08	2.27	0.10

Units of S-Ca were scaled to mmol/L across studies. Mean values present post-treatment S-Ca concentrations. [a] these are post-treatment values.

Table 2. Included studies in the meta-analysis of long-term effects of calcium plus vitamin D supplementation on S-Ca concentrations

No	Study	Setting	Mean age	N-treatment	N-control	Calcium dose (mg/d)	VD dose (IU/d)	Duration	Fasting blood	Diet	Treatment[a]		Placebo[a]	
											Mean	SD	Mean	SD
1	Orwoll et al, 1990	Community	58	41	36	1000	1000	3 y	Yes	Normal	2.37	0.10	2.36	0.10
2	Baeksgaard et al, 1998	Community	62	65	63	1000	560	2 y	NA	Normal	2.39	0.11	2.35	0.10
3	Chapuy et al, 2002	Institution	85	137	127	1200	800	2 y	Yes	Normal	2.32	0.13	2.27	0.13
4	Doetsch et al, 2004	Community	NA	16	14	1000	800	6 w	NA	Normal	2.38	0.09	2.27	0.09
5	Harwood et al, 2004	Community	81	75	75	1000	800	1 y	NA	Normal	2.42	0.18	2.40	0.20
6	Meier et al, 2004	Community	56	27	16	500	500	6 mon	No	Normal	2.34	0.06	2.35	0.08
7	Zhu et al, 2008	Community	75	39	41	1200	1000	3 y	Yes	Normal	2.42	0.07	2.36	0.12

Units of S-Ca were scaled to mmol/L across studies. Mean values present post-treatment S-Ca concentrations. [a] these are post-treatment values.

References:

1. Tai V, Leung W, Grey A, Reid IR, Bolland MJ. Calcium intake and bone mineral density: systematic review and meta-analysis. BMJ. 2015;351:h4183.
2. Recker RR, Saville PD, Heaney RP. Effect of estrogens and calcium carbonate on bone loss in postmenopausal women. Ann Intern Med. 1977;87(6):649-55.
3. Lau EM, Woo J, Leung PC, Swaminathan R, Leung D. The effects of calcium supplementation and exercise on bone density in elderly Chinese women. Osteoporos Int. 1992;2(4):168-73.
4. Riggs BL, O'Fallon WM, Muhs J, O'Connor MK, Kumar R, Melton LJ 3rd. Long-term effects of calcium supplementation on serum parathyroid hormone level, bone turnover, and bone loss in elderly women. J Bone Miner Res. 1998;13(2):168-74.
5. Ruml LA, Sakhaee K, Peterson R, Adams-Huet B, Pak CY. The effect of calcium citrate on bone density in the early and mid-postmenopausal period: a randomized placebo-controlled study. Am J Ther. 1999;6(6):303-11.
6. Son SM, Chun YN. Effect of oral therapy with alphacalcidol or calcium in Korean elderly women with osteopenia and low dietary calcium. Nutr Res. 2001;21:1347-55.
7. Rajatanavin R, Chailurkit L, Saetung S, Thakkinstian A, Nimitphong H. The efficacy of calcium supplementation alone in elderly Thai women over a 2-year period: a randomized controlled trial. Osteoporos Int. 2013;24(11):2871-7.
8. Nakamura K, Saito T, Kobayashi R, Oshiki R, Kitamura K, Oyama M, Narisawa S, Nashimoto M, Takahashi S, Takachi R. Effect of low-dose calcium supplements on bone loss in perimenopausal and postmenopausal Asian women: a randomized controlled trial. J Bone Miner Res. 2012;27(11):2264-70.
9. Orwoll ES, Oviatt SK, McClung MR, Deftos LJ, Sexton G. The rate of bone mineral loss in normal men and the effects of calcium and cholecalciferol supplementation. Ann Intern Med. 1990;112:29-34.
10. Baeksgaard L, Andersen KP, Hyldstrup L. Calcium and vitamin D supplementation increases spinal BMD in healthy, postmenopausal women. Osteoporos Int. 1998;8:255-60.
11. Chapuy MC, Pamphile R, Paris E, et al. Combined calcium and vitamin D3 supplementation in elderly women: confirmation of reversal of secondary hyperparathyroidism and hip fracture risk: the Decalyos II study. Osteoporos Int. 2002;13:257-64.
12. Doetsch AM, Faber J, Lynnerup N, Watjen I, Bliddal H, Danneskiold-Samsoe B. The effect of calcium and vitamin D3 supplementation on the healing of the proximal humerus fracture: a randomized placebo-controlled study. Calcif Tissue Int. 2004;75:183-8.
13. Harwood RH, Sahota O, Gaynor K, Masud T, Hosking DJ. A randomised, controlled comparison of different calcium and vitamin D supplementation regimens in elderly women after hip fracture: the Nottingham Neck of Femur (NONOF) Study. Age Ageing. 2004;33:45-51.
14. Meier C, Woitge HW, Witte K, Lemmer B, Seibel MJ. Supplementation with oral vitamin D3 and calcium during winter prevents seasonal bone loss: a randomized controlled open-label prospective trial. J Bone Miner Res. 2004;19:1221-30.
15. Zhu K, Devine A, Dick IM, Wilson SG, Prince RL. Effects of calcium and vitamin D supplementation on hip bone mineral density and calcium-related analytes in elderly ambulatory Australian women: a five-year randomized controlled trial. J Clin Endocrinol Metab. 2008;93:743-9.

Supplementary Methods 2: Instrument selection for S-Ca in UK Biobank

Instrumental selection for S-Ca in the UK Biobank was based on summary-level data from the Neale lab (the second-round results of analysis, http://www.nealelab.is/uk-biobank). Among participants in the UK Biobank study, those who were closely related (or at least one of a related pair of individuals) and those with sex chromosome aneuploidies were excluded. The Neale lab imputed genotypes using HRC plus UK10K & 1000 Genomes reference panels. In quality control, they excluded genetic variants with minor allele frequency <0.1% and Hardy-Weinberg equilibrium p-value $<1\times10^{-10}$ and an info score <0.8. Genome-wide association tests were adjusted for age, sex and up to 20 genetic principal components. Detailed information on sample selection, phenotype definition and genome-wide association test models can be found in the webpages for the Neale lab and UK Biobank (http://www.nealelab.is/uk-biobank). The code for data imputation and GWAS analysis can be found at https://github.com/Nealelab/UK_Biobank_GWAS. Based on this summary-level data, we first selected all SNPs associated with S-Ca at $p<5\times10^{-8}$. Among SNPs remained, independent SNPs were selected by the definition of linkage disequilibrium $r^2<0.001$ and clumping distance at 10,000 kb. Instrument selection was performed in MR-Base platform (https://www.mrbase.org/).

Supplementary Figure 1. Long-term effect of calcium supplementation on S-Ca concentrations

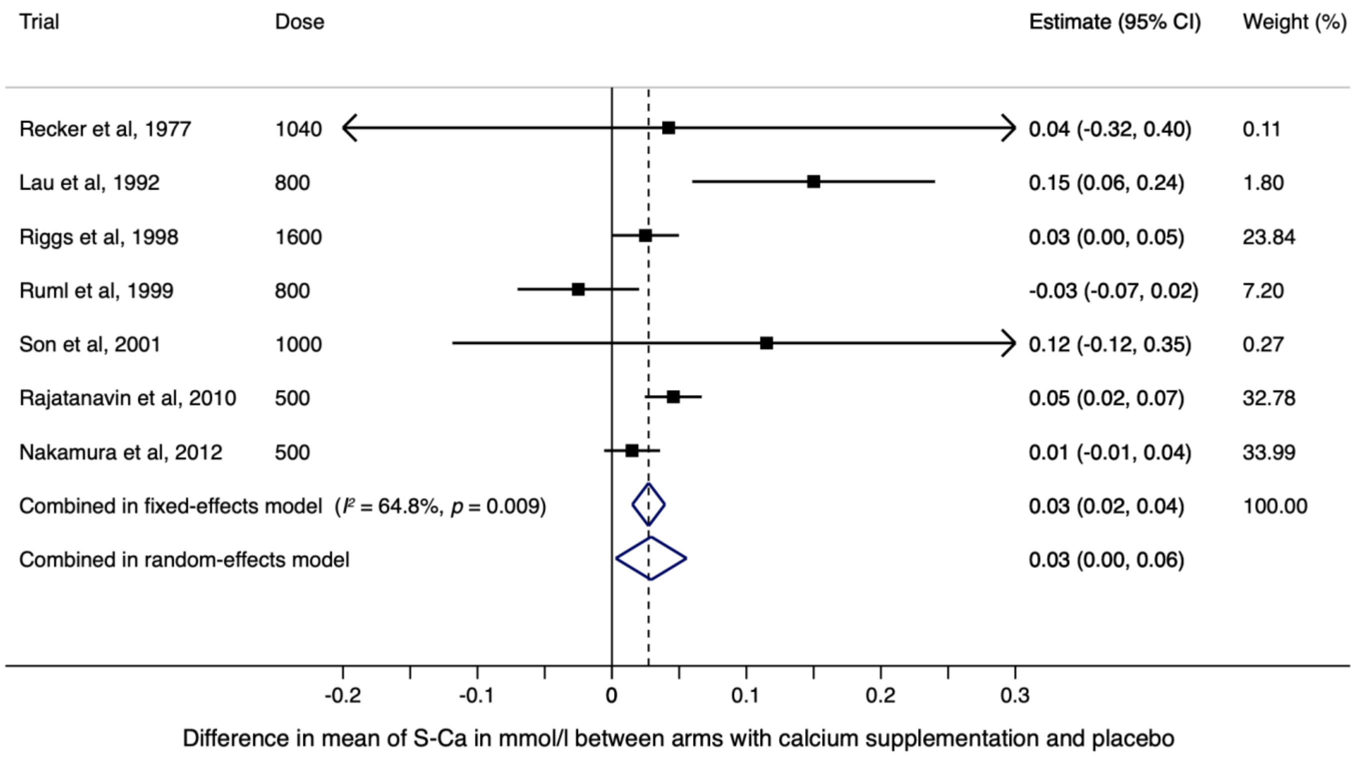

Supplementary Figure 2. Long-term effect of calcium plus vitamin D supplementation on S-Ca concentrations

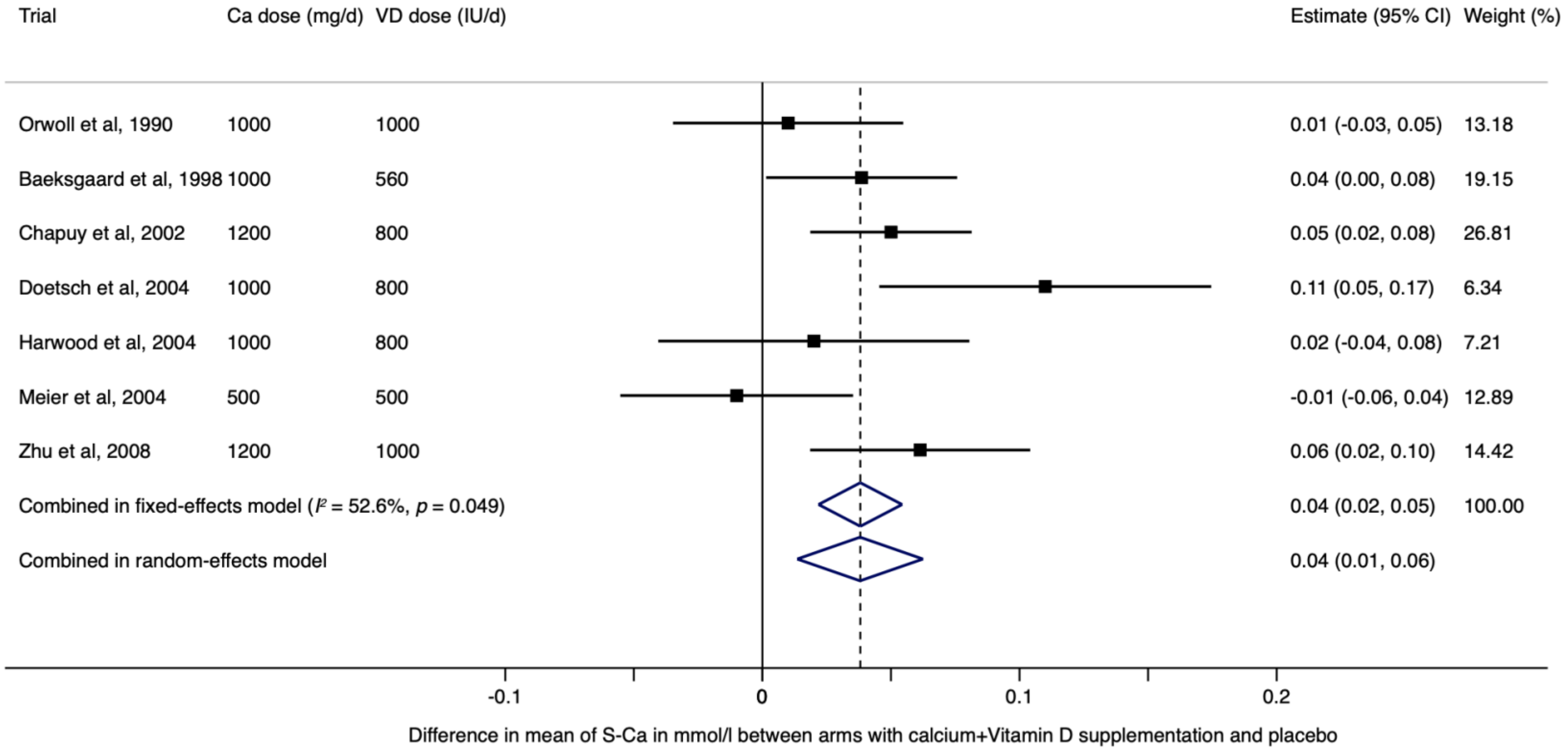

Supplementary Figure 3. Long-term effect of calcium supplementation on fasting S-Ca concentrations

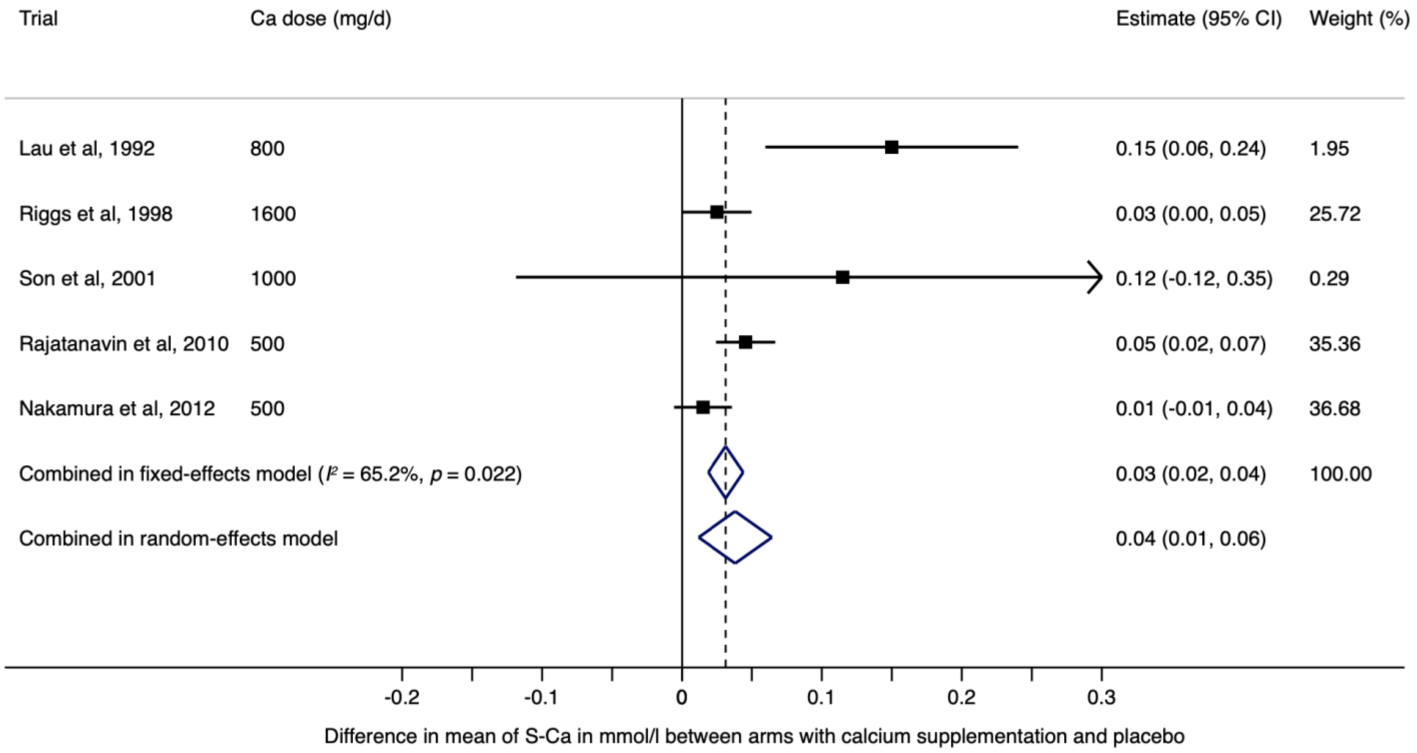

Supplementary Figure 4. Long-term effect of calcium plus vitamin D supplementation on fasting S-Ca concentrations

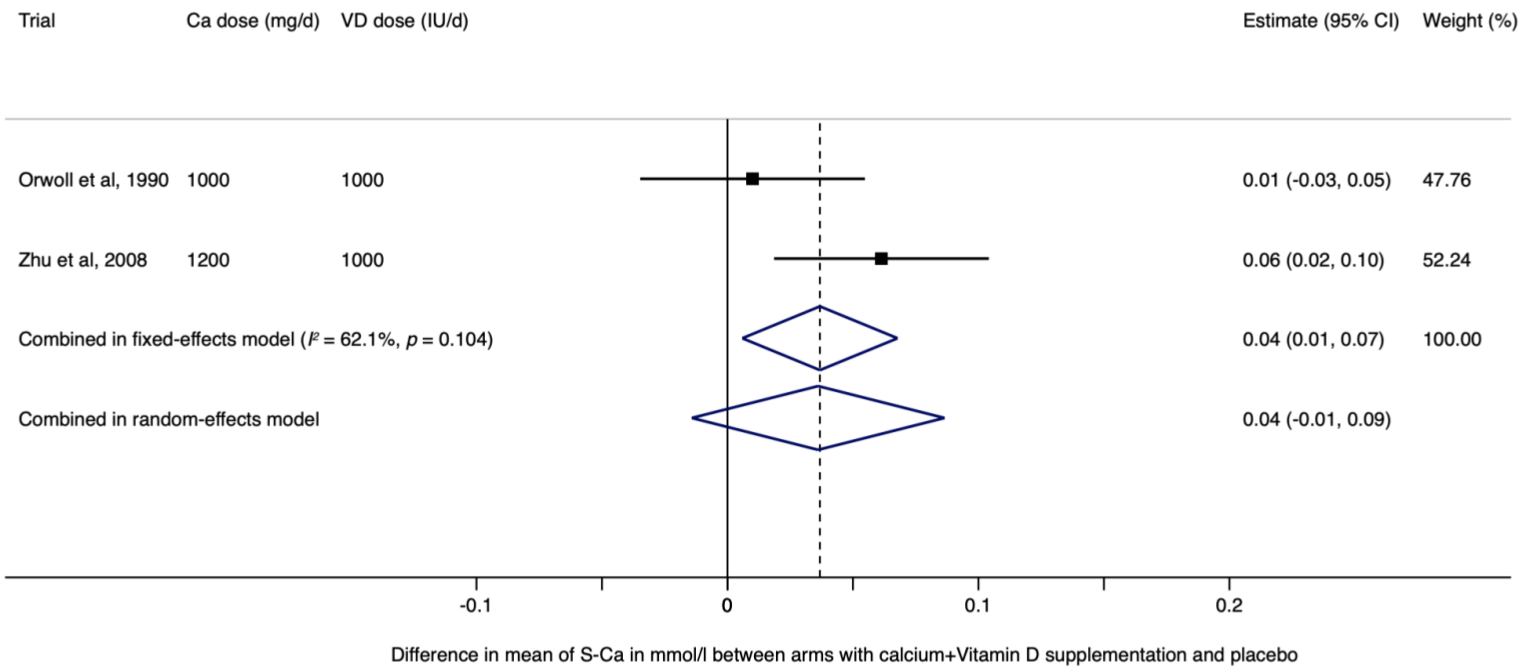

Supplementary Figure 5. Funnel plots for two meta-analyses

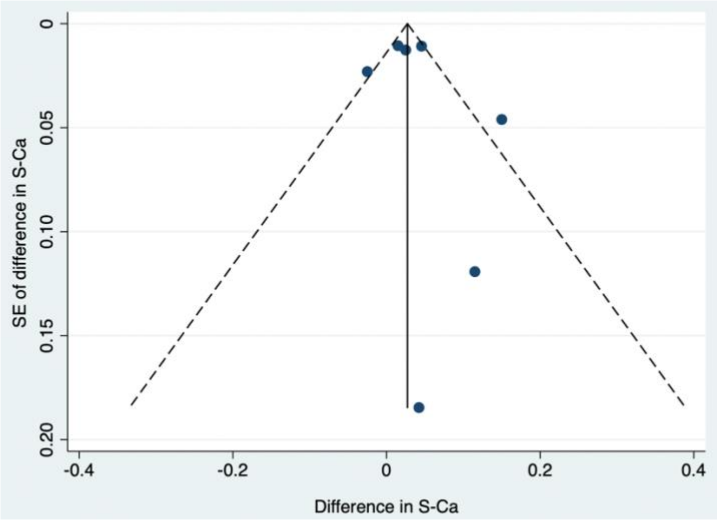

Meta-analysis of calcium supplementation

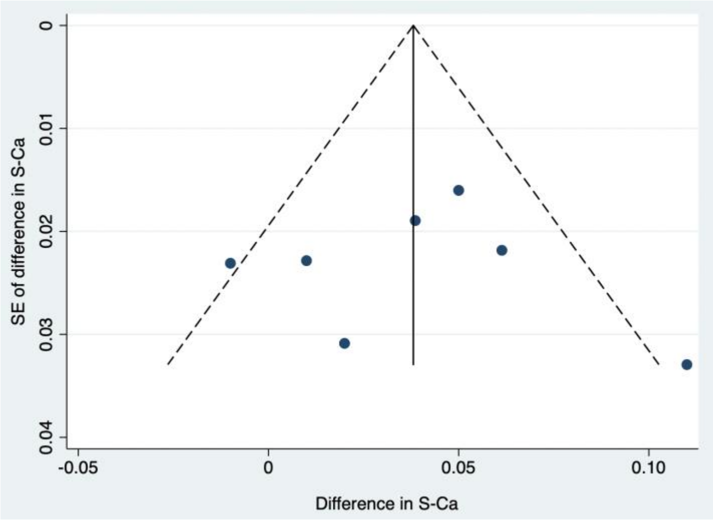

Meta-analysis of calcium plus vitamin D supplementation

Supplementary Table 1. Studies and consortia used in the present study

Exposure or outcome	Data source	Participants [a]	PubMed ID or webpage
Serum calcium	Meta-analysis of GWASs	Up to 61,054 participants of European ancestry from the discovery (17 studies) and replication (11 studies) stages	24068962
Serum calcium	UK Biobank	315,153 participants of European ancestry from the UK Biobank study	http://www.nealelab.is/uk-biobank
Serum 25(OH)D	Meta-analysis of GWASs	42,274 participants of European ancestry for rs117913124 and 79,366 participants of European ancestry for other six SNPs	rs117913124 (28757204) and other SNPs (29343764)
Serum 25(OH)D	UK Biobank	417,580 participants of European ancestry from the UK Biobank study	32242144
Longevity	Meta-analysis of GWASs (Deelen J, et al)	11,262 European-descent participants who lived beyond an age at or above the 90_{th} survival percentile (as cases) and 25,483 controls who died at or before the age at the 60_{th} survival percentile or whose age at the last follow-up visit was at or before the 60_{th} survival percentile	31413261
Longevity	UK Biobank (Pilling LC, et al)	7,182 European-descent participants whose both parents with longevity in top 10% and 79,767 controls	27015805
Cardiovascular disease	FinnGen consortium	111,108 Finnish-descent participants with at least one cardiovascular disease and 107,684 controls	https://www.finngen.fi/fi
Cancer	FinnGen consortium	38,036 Finnish-descent participants with at least one malignant neoplasm and 180,756 controls	https://www.finngen.fi/fi

[a] The GWASs included in the meta-analysis for calcium are listed in Text S1: https://journals.plos.org/plosgenetics/article?id=10.1371/journal.pgen.1003796#s5. The GWASs included in the meta-analysis for 25(OH)D are listed in the Methods part: https://www.nature.com/articles/s41467-017-02662-2#Sec9 and in the Table 1 (for rs117913124): https://www.cell.com/ajhg/fulltext/S0002-9297(17)30277-X#secsectitle0060. Meta-analyses of GWAS for both calcium and 25(OH)D did not include UK Biobank study. The summary-level data of the meta-analysis of GWAS on longevity was based on the discovery and replication stages, where individuals GWAS was list in Table 1: https://www.nature.com/articles/s41467-019-11558-2#data-availability (this meta-analysis of GWAS on longevity did not include the UK Biobank study).

Supplementary Table 2. Diagnostic information for cardiovascular disease and cancer in FinnGen

<table>
<tr><th colspan="2">Included outcomes</th><th>Diagnostic information</th></tr>
<tr><th>Primary outcome</th><th>Secondary outcome</th><th>Hospital discharge registry</th></tr>
<tr><td rowspan="4">Heart disease (rheumatic fever)</td><td>Rheumatic fever with heart involvement</td><td>ICD-10: I020/I01, ICD-9: 391, ICD-8: 391</td></tr>
<tr><td>Rheumatic fever without mention of heart involvement</td><td>ICD-10: I029/I00, ICD-9: 390, ICD-8: 390</td></tr>
<tr><td>Rheumatic valve diseases</td><td>ICD-10: I05/I06/I08/I07, ICD-9: 394/395/396, ICD-8: 3940|3950|3960|3970</td></tr>
<tr><td>Rheumatic heart disease, other</td><td>ICD-10: I09, ICD-9: 3920A/397/393/398, ICD-8: 3920|393|3979|398</td></tr>
<tr><td rowspan="3">Hypertensive diseases</td><td>Hypertension, Pulmonary Arterial</td><td>ICD-10: I270, ICD-9: 4160A, ICD-8: 42600</td></tr>
<tr><td>Hypertensive heart and/or renal disease</td><td>ICD-10: I11/I13/I12, ICD-9: 4029A/4029B/4039A/4040A, ICD-8: 40299|40399|40499</td></tr>
<tr><td>Hypertension</td><td>ICD-10: I13/I674/I11/I10/I12/I15, ICD-9: 4019X/4029A/4029B/4039A/4040A/4059A/4059B/4059X/4372A, ICD-8: 400|401|402|403|404</td></tr>
<tr><td rowspan="7">Ischemic heart diseases</td><td>Angina pectoris</td><td>ICD-10: I20, ICD-9: 413, ICD-8: 413</td></tr>
<tr><td>Myocardial infarction</td><td>ICD-10: I22/I21, ICD-9: 410, ICD-8: 410</td></tr>
<tr><td>Complications following myocardial infarction</td><td>ICD-10: I23</td></tr>
<tr><td>Status post-ami</td><td>ICD-10: I253, ICD-9: 412, ICD-8: 412</td></tr>
<tr><td>Coronary atherosclerosis</td><td>ICD-10: T822/Z951/I25/I24, ICD-9: 414/9960A, ICD-8: 414</td></tr>
<tr><td>Coronary angiopasty</td><td>Operation codes: ^FNF|^FNG|^TFN40|^FN1AT|^FN1BT|^FN1YT, ^82$|^83$|^84$, ^AN2|^AN3|^AN4|^ANA|^ANB</td></tr>
<tr><td>Coronary artery bypass grafting</td><td>Operation codes: ^FNA|^FNB|^FNC|^FND|^FNE, ^5311$|^5312$|^5313$|^5314$|^5315$, ^11$|^25$|^111$|^112$|^113$|^119$, ^AA1|^AA2|^AA3|^AAX</td></tr>
<tr><td rowspan="2">Pulmonary heart disease</td><td>Pulmonary embolism</td><td>ICD-10: I26, ICD-9: 415, ICD-8: 450</td></tr>
<tr><td>Other pulmonary heart/vessel disease</td><td>ICD-10: I28/I27, ICD-9: 416/417, ICD-8: 426</td></tr>
<tr><td rowspan="10">Other heart diseases</td><td>Pericarditis</td><td>ICD-10: I30/I32, ICD-9: 420, ICD-8: 420</td></tr>
<tr><td>Other diseases of pericardium</td><td>ICD-10: I31, ICD-9: 423, ICD-8: 423</td></tr>
<tr><td>Endocarditis</td><td>ICD-10: I38/I39/I33, ICD-9: 0932A/421, ICD-8: 421</td></tr>
<tr><td>Non-rheumatic valve diseases</td><td>ICD-10: I35/I34/I36/I37, ICD-9: 424, ICD-8: 39[4-6]65|424</td></tr>
<tr><td>Valvular operations</td><td>^FGA|^FGC|^FGD|^FGE|^FGW|^F^JE|^F^JF|^FK|^FM, ^5261$|^527|^528|^529|^530</td></tr>
<tr><td>Myocarditis</td><td>ICD-10: I41/I40, ICD-9: 422, ICD-8: 0742|422</td></tr>
<tr><td>Cardiomyopathy</td><td>ICD-10: I42</td></tr>
<tr><td>Conduction disorders</td><td>ICD-10: I44/I45, ICD-9: 426</td></tr>
<tr><td>Cardiac arrest</td><td>ICD-10: I46, ICD-9: 4275A, ICD-8: 4272</td></tr>
</table>

	Paroxysmal tachycardia	ICD-10: I47, ICD-8: 4275
	Atrial fibrillation and flutter	ICD-10: I48, ICD-8: 42792
	Heart failure, strict	ICD-10: I50/I130/I132/I110, ICD-9: 4029B/428, ICD-8: 42700|42710|428|7824
	Other arrhytmias	ICD-10: I49, ICD-9: 427, ICD-8: 42798, exclude ICD-9: 4273, ICD-8: 42792
	Other or ill-defined heart diseases	ICD-10: I52/I51, ICD-9: 429, ICD-8: 42899|42999
Cerebrovascular diseases	Subarachnoid haemmorrhage	ICD-10: I60, ICD-9: 430, ICD-8: 430
	Intracerebral haemmorrhage	ICD-10: I61, ICD-9: 431, ICD-8: 431
	Other intracranial haemorrhages	ICD-10: I62
	Ischaemic stroke	ICD-10: I63/I64, ICD-9: 436/4340A/4331A/4330A/4349A/4341A/4339A, ICD-8: 433|434|436, exclude ICD-10: I636
	Occlusion and stenosis of arteries, not leading to stroke	ICD-10: I66, ICD-9: 4330X/4331X/4339X
	Dissection of cerebral arteries, nonruptured	ICD-10: I670
	Cerebral aneurysm, nonruptured	ICD-10: I671
	Endovascular or surgical operations to intracerebral aneurysms	Operation codes: ^AAC0|^AAC1|^AAC9|^AAL0, ^2161|^2162|^2163|^2164|^2165|^2169|^2160|^2166|^2167|^2168
	Cerebral atherosclerosis	ICD-10: I672, ICD-8: 437
	Progressive vascular leukoencephalopathy	ICD-10: I673
	Hypertensive encephalopathy	ICD-10: I674
	Moyamoya	ICD-10: I675
	Nonpyogenic thrombosis of intracranial venous system	ICD-10: I676
	Cerebral arteritis	ICD-10: I677/I682
	Other specified cerebrovascular diseases	ICD-10: I688/I678/I679, ICD-9: 436/4378X, ICD-8: 438
	Cerebral amyloid angiopathy	ICD-10: I680
	Sequelae of cerebrovascular disease	ICD-10: I69, ICD-9: 438
Diseases of arteries, arterioles and capillaries	Atherosclerosis, excluding cerebral, coronary and PAD	ICD-10: I70, ICD-9: 440, ICD-8: 440
	Aortic aneurysm	ICD-10: I718/I713/I711/I712/I716/I719/I714/I715, ICD-9: 0930A, ICD-8: 0930|441
	Other aneurysm	ICD-10: I72, ICD-9: 442, ICD-8: 442
	Other peripheral vascular diseases	ICD-10: I73, ICD-9: 443, ICD-8: 443
	Embolism and thrombosis of abdominal aorta	CD-10: I740, ICD-9: 4440A, ICD-8: 44400, exclude ICD-10: 4440
	Arterial embolism and thrombosis of lower extremity artery	ICD-10: I743, ICD-9: 4442A
	Other arterial embolism and thrombosis	ICD-10: I74, ICD-9: 444, ICD-8: 444, exclude ICD-10: I743|I740; ICD-9: 4440A|4442A, ICD-8: 4440
	Other diseases of arteries and capillaries	ICD-10: I77/I78, ICD-9: 447/448, ICD-8: 447|448
	Peripheral artery disease	ICD-10: E105/E145/E135/I739/E125/E115/I702, ICD-8: 25006|4402|4439
	Vascular diseases of the intestine	ICD-10: K550/K551/K559, ICD-9: 557, ICD-8: 4442

Diseases of veins, lymphatic vessels and lymph nodes, not elsewhere classified	Phlebitis and thrombophlebitis (not including DVT)	ICD-10: I80, ICD-9: 4518X/4519X, ICD-8: 451, exclude IVD-10: I802|I803, ICD-8: 4510.
	DVT of lower extremities	ICD-10: I803, ICD-9: 4510A/4511A/4512A, ICD-8: 4510
	Portal vein thrombosis	ICD-10: I81, ICD-9: 452, ICD-8: 452
	Other embolism and thrombosis	ICD-10: I82, ICD-9: 453, ICD-8: 453
	Varicose veins	ICD-10: I83, ICD-9: 454, ICD-8: 454
	Oesophageal varices	ICD-10: I85, ICD-8: 4560
	Varicose veins of other sites	ICD-10: I86, ICD-9: 456, ICD-8: 456, exclude ICD-9: 456[0-1], ICD-8: 4560
	Other disorders of veins	ICD-10: I87
	Nonspesific lymphadenitis	ICD-10: I88, ICD-8: 289[1-3]
	Other noninfective disordersof lymphatic vessels and lymph nodes	ICD-10: I89, ICD-9: 457, ICD-8: 457
Other CVD	Hypotension	ICD-10: I95, ICD-9: 458, ICD-8: 4580
	Other other unspecified disorders of the circulatory system	ICD-10: I99/I97, ICD-9: 4590A, ICD-8: 4581|4589
Malignant neoplasm of lip, oral cavity and pharynx	Malignant neoplasm of base of tongue	ICD-10: C01, ICD-9: 1410A, ICD-8: 1410
	Malignant neoplasm of other and unspecified parts of tongue	ICD-10: C02, ICD-9: 141, ICD-8: 141, exclude ICD-9: 1410A, ICD-8: 1410.
	Malignant neoplasm of floor of mouth	ICD-10: C04, ICD-9: 144, ICD-8: 144
	Malignant neoplasm of other and unspecified parts of mouth	ICD-10: C06, ICD-9: 145, ICD-8: 145
	Malignant neoplasm of tonsil	ICD-10: C09, ICD-9: 1460A, ICD-8: 1460
	Malignant neoplasm of oropharynx	ICD-10: C10, ICD-9: 146, ICD-8: 146, exclude ICD-9: 1460A
	Malignant neoplasm of nasopharynx	ICD-10: C11, ICD-9: 147, ICD-8: 147
	Malignant neoplasm of piriform sinus	ICD-10: C12, ICD-9: 1481A, ICD-8: 1481
	Malignant neoplasm of hypopharynx	ICD-10: C13, ICD-9: 148, ICD-8: 148, exclude ICD-9: 1481A, ICD-8: 1481
	Malignant neoplasm of other and ill-defined sites in the lip, oral cavity and pharynx	ICD-10: C14, ICD-9: 149, ICD-8: 149
Malignant neoplasm of digestive organs	Malignant neoplasm of oesophagus	ICD-10: C15, ICD-9: 150, ICD-8: 150
	Malignant neoplasm of stomach	ICD-10: C16, ICD-9: 151, ICD-8: 151
	Malignant neoplasm of small intestine	ICD-10: C17, ICD-9: 152, ICD-8: 152
	Malignant neoplasm of colon	ICD-10: C18, ICD-9: 153, ICD-8: 153
	Malignant neoplasm of rectosigmoid junction	ICD-10: C19, ICD-8: 1540
	Malignant neoplasm of rectum	ICD-10: C20, ICD-9: 154, ICD-8: 1541
	Malignant neoplasm of anus and anal canal	ICD-10: C21, ICD-8: 1542
	Malignant neoplasm of liver and intrahepatic bile ducts	ICD-10: C22, ICD-9: 155, ICD-8: 155
	Malignant neoplasm of gallbladder	ICD-10: C23, ICD-8: 1560

	Malignant neoplasm of other and unspecified parts of biliary tract	ICD-10: C24, ICD-9: 156, ICD-8: 156
	Malignant neoplasm of other and ill-defined digestive organs	ICD-10: C26, ICD-9: 158/159, ICD-8: 15[8-9]
	Malignant neoplasm of pancreas	ICD-10: C25, ICD-9: 157, ICD-8: 157
Malignant neoplasm of bone and articular cartilage	Malignant neoplasm of bone and articular cartilage of limbs	ICD-10: C40, ICD-8: 170[4-5]|170[7-8]
	Malignant neoplasm of bone and articular cartilage of other and unspecified sites	ICD-10: C41, ICD-9: 170, ICD-8: 170
Malignant neoplasm of skin	Malignant melanoma of skin	ICD-10: C43, ICD-9: 172, ICD-8: 172
	Non-melanoma skin cancer	ICD-10: C44, ICD-9: 173, ICD-8: 173
Melignant neoplasm of mesothelium and soft tissue	Mesothelioma	ICD-10: C45, ICD-9: 158/163, ICD-8: 158|163
	Kaposi sarcoma	ICD-10: C46
	Malignant neoplasm of peripheral nerves and autonomic nervous system	ICD-10: C47, ICD-8: 192[4-5]
	Malignant neoplasm of retroperitoneum and peritoneum	ICD-10: C48
	Malignant neoplasm of other connective and soft tissue	ICD-10: C49, ICD-9: 171, ICD-8: 171
Malignant neoplasm of breast	Malignant neoplasm of breast	ICD-10: C50, ICD-9: 174, ICD-8: 174
Malignant neoplasm of female genital organs	Malignant neoplasm of vulva	ICD-10: C51, ICD-8: 1841
	Malignant neoplasm of vagina	ICD-10: C52, ICD-8: 1840
	Malignant neoplasm of cervix uteri	ICD-10: C53, ICD-9: 180, ICD-8: 180
	Malignant neoplasm of corpus uteri	ICD-10: C54, ICD-9: 182, ICD-8: 1820
	Malignant neoplasm of uterus, part unspecified	ICD-10: C55, ICD-8: 182, exclude ICD-8: 1820
	Malignant neoplasm of ovary	ICD-10: C56, ICD-9: 183, ICD-8: 1830
	Malignant neoplasm of other and unspecified female genital organs	ICD-10: C57, ICD-9: 184, ICD-8: 183, exclude ICD-8: 1830
Malignant neoplasm of male genital organs	Malignant neoplasm of penis	ICD-10: C60, ICD-8: 1870
	Malignant neoplasm of prostate	ICD-10: C61, ICD-9: 185, ICD-8: 185
	Malignant neoplasm of testis	ICD-10: C62, ICD-9: 186, ICD-8: 186
	Malignant neoplasm of other and unspecified male genital organs	ICD-10: C63, ICD-9: 187, ICD-8: 187, exclude ICD-9: 187[1-4], ICD-8: 1870
Malignant neoplasm of urinary organs	Malignant neoplasm of kidney, except renal pelvis	ICD-10: C64, ICD-8: 1890
	Malignant neoplasm of renal pelvis	ICD-10: C65, ICD-8: 1891
	Malignant neoplasm of ureter	ICD-10: C66, ICD-8: 1892
	Malignant neoplasm of bladder	ICD-10: C67, ICD-9: 188, ICD-8: 188

	Malignant neoplasm of other and unspecified urinary organs	ICD-10: C68, ICD-8: 1899
Malignant neoplasm of eye, brain and central nervous system	Malignant neoplasm of eye and adnexa	ICD-10: C69, ICD-9: 190, ICD-8: 190
	Malignant neoplasm of meninges	ICD-10: C70, ICD-8: 192[1-2]
	Malignant neoplasm of brain	ICD-10: C71, ICD-9: 191, ICD-8: 191
	Malignant neoplasm of spinal cord, cranial nerves and other parts of central nervous system	ICD-10: C72, ICD-9: 192, ICD-8: 192
Malignant neoplasm of endocrine gland	Malignant neoplasm of thyroid gland	ICD-10: C73, ICD-9: 193, ICD-8: 193
	Malignant neoplasm of adrenal gland	ICD-10: C74, ICD-8: 1940
	Malignant neoplasm of other endocrine glands and related structures	ICD-10: C75, ICD-8: 194[1-9]
Secondary uncertain malignant neoplasm	Malignant neoplasm of other and ill-defined sites	ICD-10: C76, ICD-9: 195, ICD-8: 195
	Secondary and unspecified malignant neoplasm of lymph nodes	ICD-10: C77, ICD-8: 196
	Secondary malignant neoplasm of respiratory and digestive organs	ICD-10: C78, ICD-8: 197
	Secondary malignant neoplasm of other and unspecified sites	ICD-10: C79, ICD-8: 198
	Malignant neoplasm, without specification of site	ICD-10: C80, ICD-9: 199, ICD-8: 199
Primary lymphoid and hematopoietic malignant neoplasms	Hodgkin lymphoma	ICD-10: C81, ICD-9: 201
	Follicular lymphoma	ICD-10: C82, ICD-9: 2000A
	Non-follicular lymphoma	ICD-10: C83, ICD-9: 200, exclude ICD-9: 200A
	Mature T/NK-cell lymphomas	ICD-10: C84
	Other and unspecified types of non-Hodgkin lymphoma	ICD-10: C85
	Malignant immunoproliferative diseases	ICD-10: C88
	Multiple myeloma and malignant plasma cell neoplasms	ICD-10: C90, ICD-9: 203, ICD-8: 203
	Lymphoid leukaemia	ICD-10: C91, ICD-9: 204, ICD-8: 204
	Myeloid leukaemia	ICD-10: C92, ICD-9: 205, ICD-8: 205
	Monocytic leukaemia	ICD-10: C93, ICD-9: 206, ICD-8: 206
	Other leukaemias of specified cell type	ICD-10: C94, ICD-8: 207[0-2]
	Leukaemia of unspecified cell type	ICD-10: C95, ICD-9: 207/208, ICD-8: 207, exclude ICD-9: 207[0-8], ICD-8: 207[0-2]
	Other and unspecified malignant neoplasms of lymphoid, haematopoietic and related tissue	ICD-10: C96, ICD-9: 202, ICD-8: 202
Malignant neoplasms of independent	Malignant neoplasms of independent (primary) multiple sites	ICD-10: C97

(primary) multiple sites

Supplementary Table 3. Associations of genetically predicted serum calcium and 25(OH)D with parent lifespans

**Exposure**	**SNPs**	**Method**	**Change in years**	**95% CI**	***p***
**Serum calcium**					
SNPs from GWAS meta-analysis	7	IVW-random effects	-0.12	-0.24, 0.00	0.050
		Weighted median	-0.08	-0.22, 0.06	0.247
		MR-Egger	-0.04	-0.27, 0.18	0.697
		Cochrane's Q = 5.63 (p = 0.466); MR-Egger intercept = -0.003 (p = 0.433)			
SNPs from UKBB	140	IVW-random effects	-0.03	-0.06, 0.01	0.100
		Weighted median	-0.07	-0.11, -0.03	<0.001
		MR-Egger	-0.06	-0.10, -0.01	0.030
		Cochrane's Q = 248.11 (p < 0.001); MR-Egger intercept = 0.001 (p = 0.143)			
**Serum 25(OH)D**					
SNPs from GWAS meta-analysis	7	IVW-random effects	0.00	-0.02, 0.03	0.810
		Weighted median	-0.01	-0.03, 0.02	0.647
		MR-Egger	-0.02	-0.06, 0.02	0.398
		Cochrane's Q = 5.73 (p = 0.454); MR-Egger intercept = 0.004 (p = 0.214)			
SNPs from UKBB	115	IVW-random effects	0.02	-0.03, 0.07	0.403
		Weighted median	-0.01	-0.05, 0.03	0.574
		MR-Egger	0.01	-0.05, 0.07	0.820
		Cochrane's Q = 405.06 (p < 0.001); MR-Egger intercept = 0.001 (p = 0.501)			

CI, confidence interval; IVW, inverse-variance weighted; SNPs, single nucleotide polymorphisms.

Supplementary Table 4. Associations of genetically predicted serum calcium and 25(OH)D with longevity in sensitivity analyses

Data source	SNPs	Heterogeneity testing		Weighted median estimates		MR-Egger regression		P_{plei}
		Cochrane's Q	P_Q	OR (95% CI)	*P*	OR (95% CI)	*P*	
Serum calcium								
SNPs from GWAS meta-analysis								
Deelen et al	7	2.87	0.825	0.77 (0.54, 1.10)	0.140	0.77 (0.45, 1.32)	0.345	0.982
Pilling et al	6	5.05	0.410	0.36(0.14, 0.93)	0.035	0.38(0.07, 2.15)	0.337	0.099
SNPs from UKBB								
Deelen et al	175	220.27	0.010	0.76(0.58, 1.00)	0.045	0.99(0.68, 1.44)	0.957	0.750
Pilling et al	175	219.42	0.011	0.63(0.48, 0.83)	0.001	0.88(0.62, 1.26)	0.487	0.411
Serum 25(OH)D								
SNPs from GWAS meta-analysis								
Deelen et al	7	4.22	0.647	0.94 (0.82, 1.08)	0.387	0.95 (0.77, 1.16)	0.601	0.848
Pilling et al	6	17.52	0.008	0.81(0.68, 0.97)	0.024	0.69(0.44, 1.06)	0.150	0.168
SNPs from UKBB								
Deelen et al	115	226.65	<0.001	0.92 (0.77, 1.11)	0.399	0.89 (0.71, 1.12)	0.331	0.086
Pilling et al	105	247.08	<0.001	0.76(0.6, 0.97)	0.029	0.8(0.6, 1.08)	0.143	0.033

25(OH)D indicates 25-hydroxyvitamin D; CI indicates confidence interval; P_{dist}, *p* value for distortion test in MR-PRESSO; P_{plei}, *p* value for pleiotropy (the intercept in MR-Egger regression); P_Q, *p* value for Cochrane Q; SNPs, single-nucleotide polymorphisms; UKBB, UK Biobank.
Heterogeneity was observed in the analyses of serum calcium and 25(OH)D instrumented by UKBB data. *P* values for intercept in the MR-Egger statistic in the analysis of calcium were >0.05, which indicated balanced pleiotropy in these analyses. However, an imbalanced pleiotropy might occur in the analysis of 25(OH)D based on UKBB and data from Pilling et al (P_{plei} <0.05). There were consistent inverse associations of serum calcium with longevity in the weighted median model. MR-PRESSO detected outliers in the analysis of 25(OH)D based on GWAS meta-analysis in relation to longevity by Pilling LC et al (the outlier-corrected OR, 0.81; 95% CI, 0.72, 0.90; *p*=0.013) and the analysis of 25(OH)D based on UKBB in relation to longevity by Deelen et al (OR, 0.98; 95% CI, 0.84, 1.14; *p*=0.807) and Pilling et al. (OR, 0.93; 95% CI, 0.76, 1.14; *p*=0.498).

Supplementary Table 5. Associations of genetically predicted serum calcium and 25(OH)D with cardiovascular disease and cancer in sensitivity analyses

Data source	SNPs	Heterogeneity		Weighted median		MR-Egger		P_{plei}
		Cochrane's Q	P_Q	OR (95% CI)	*P*	OR (95% CI)	*P*	
Serum calcium								
SNPs from GWAS meta-analysis								
Cardiovascular disease	7	2.37	0.882	1.07 (0.90, 1.23)	0.382	1.02 (0.82, 1.28)	0.868	0.414
Cancer	7	7.46	0.280	0.96 (0.81, 1.12)	0.590	0.96 (0.74, 1.25)	0.789	0.188
SNPs from UKBB								
Cardiovascular disease	174	224.03	0.005	1.14 (1.02, 1.27)	0.019	1.19 (1.02, 1.40)	0.029	0.456
Cancer	174	184.39	0.263	1.08 (0.96, 1.22)	0.204	1.00 (0.85, 1.18)	0.965	0.993
Serum 25(OH)D								
SNPs from GWAS meta-analysis								
Cardiovascular disease	7	3.41	0.757	1.03 (0.97, 1.09)	0.313	1.01 (0.93, 1.11)	0.770	0.689
Cancer	7	2.83	0.830	0.97 (0.90, 1.05)	0.511	1.00 (0.90, 1.10)	0.983	0.825
SNPs from UKBB								
Cardiovascular disease	120	165.16	0.003	1.03 (0.96, 1.11)	0.431	1.01 (0.93, 1.09)	0.880	0.296
Cancer	120	122.55	0.393	0.93 (0.85, 1.03)	0.163	1.00 (0.93, 1.08)	0.961	0.389

25(OH)D indicates 25-hydroxyvitamin D; CI, confidence interval; P_{dist}, *p* value for distortion test in MR-PRESSO; P_{plei}, *p* value for pleiotropy (the intercept in MR-Egger regression); P_Q, *p* value for Cochrane Q; SNPs, single-nucleotide polymorphisms; UKBB, UK Biobank.
Heterogeneity was observed in the analysis of serum calcium instrumented by UKBB data and cardiovascular disease, and the analysis of serum 25(OH)D instrumented by UKBB data and cardiovascular disease. *P* values for intercept in MR-Egger in this analysis was >0.05, which indicated balanced pleiotropy. There were positive associations of serum calcium (UKBB) with cardiovascular disease in the weighted median and MR-Egger models. MR-PRESSO detected one outlier in the analysis of 25(OH)D based on UKBB in relation to cardiovascular disease (the outlier-corrected OR, 0.99; 95% CI, 0.93, 1.04; *p*=0.645).

Supplementary Table 6. Data for analyses of the association of serum calcium with longevity, cardiovascular disease, and cancer

Exposure	Outcome	SNP	EA	NEA	Exposure_beta	Exposure_se	Outcome_beta	Outcome_se
Calcium_O'Seaghdha_CM	CA_FinnGen	rs10491003	T	C	0.0540	0.0100	-0.020	0.014
Calcium_O'Seaghdha_CM	CA_FinnGen	rs1550532	C	G	0.0360	0.0060	-0.008	0.010
Calcium_O'Seaghdha_CM	CA_FinnGen	rs1570669	G	A	0.0360	0.0060	-0.003	0.009
Calcium_O'Seaghdha_CM	CA_FinnGen	rs1801725	T	G	0.1420	0.0080	-0.005	0.013
Calcium_O'Seaghdha_CM	CA_FinnGen	rs7336933	G	A	0.0440	0.0080	0.011	0.012
Calcium_O'Seaghdha_CM	CA_FinnGen	rs7481584	G	A	0.0360	0.0060	-0.006	0.011
Calcium_O'Seaghdha_CM	CA_FinnGen	rs780094	T	C	0.0340	0.0060	0.005	0.009
Calcium_O'Seaghdha_CM	CVD_FinnGen	rs10491003	T	C	0.0540	0.0100	0.006	0.013
Calcium_O'Seaghdha_CM	CVD_FinnGen	rs1550532	C	G	0.0360	0.0060	0.007	0.008
Calcium_O'Seaghdha_CM	CVD_FinnGen	rs1570669	G	A	0.0360	0.0060	0.005	0.008
Calcium_O'Seaghdha_CM	CVD_FinnGen	rs1801725	T	G	0.1420	0.0080	0.009	0.012
Calcium_O'Seaghdha_CM	CVD_FinnGen	rs7336933	G	A	0.0440	0.0080	0.004	0.010
Calcium_O'Seaghdha_CM	CVD_FinnGen	rs7481584	G	A	0.0360	0.0060	0.016	0.009
Calcium_O'Seaghdha_CM	CVD_FinnGen	rs780094	T	C	0.0340	0.0060	0.002	0.008
Calcium_O'Seaghdha_CM	Longevity_90%	rs780094	T	C	0.0340	0.0060	-0.028	0.020
Calcium_O'Seaghdha_CM	Longevity_90%	rs1550532	C	G	0.0360	0.0060	-0.015	0.021
Calcium_O'Seaghdha_CM	Longevity_90%	rs1801725	T	G	0.1420	0.0080	-0.037	0.027
Calcium_O'Seaghdha_CM	Longevity_90%	rs10491003	T	C	0.0540	0.0100	-0.023	0.034
Calcium_O'Seaghdha_CM	Longevity_90%	rs7481584	G	A	0.0360	0.0060	-0.009	0.022
Calcium_O'Seaghdha_CM	Longevity_90%	rs7336933	G	A	0.0440	0.0080	-0.010	0.027
Calcium_O'Seaghdha_CM	Longevity_90%	rs1570669	G	A	0.0360	0.0060	0.018	0.020
Calcium_O'Seaghdha_CM	Longevity_90%_UKBB	rs10491003	T	C	0.0270	0.0050	-0.039	0.045
Calcium_O'Seaghdha_CM	Longevity_90%_UKBB	rs1570669	G	A	0.0180	0.0030	-0.025	0.028
Calcium_O'Seaghdha_CM	Longevity_90%_UKBB	rs1801725	T	G	0.0710	0.0040	-0.069	0.039
Calcium_O'Seaghdha_CM	Longevity_90%_UKBB	rs7336933	G	A	0.0220	0.0040	0.055	0.036
Calcium_O'Seaghdha_CM	Longevity_90%_UKBB	rs7481584	G	A	0.0180	0.0030	-0.039	0.029
Calcium_O'Seaghdha_CM	Longevity_90%_UKBB	rs780094	T	C	0.0170	0.0030	-0.012	0.027
Calcium_UKBB	CA_FinnGen	rs1036332	C	A	0.0166	0.0022	0.005	0.010
Calcium_UKBB	CA_FinnGen	rs10513810	G	A	-0.0204	0.0030	-0.010	0.012
Calcium_UKBB	CA_FinnGen	rs10754439	T	G	0.0113	0.0020	-0.006	0.009
Calcium_UKBB	CA_FinnGen	rs10819178	G	T	0.0251	0.0020	-0.011	0.009

Calcium_UKBB	CA_FinnGen	rs10858935	A	G	-0.0142	0.0021	0.007	0.009
Calcium_UKBB	CA_FinnGen	rs10917386	T	C	0.0152	0.0021	0.019	0.010
Calcium_UKBB	CA_FinnGen	rs10958700	G	T	0.0152	0.0023	-0.019	0.012
Calcium_UKBB	CA_FinnGen	rs11078597	C	T	0.0386	0.0025	0.012	0.011
Calcium_UKBB	CA_FinnGen	rs11117777	C	T	0.0156	0.0027	0.014	0.012
Calcium_UKBB	CA_FinnGen	rs112174050	T	C	0.0804	0.0062	0.030	0.019
Calcium_UKBB	CA_FinnGen	rs113955164	A	G	0.0158	0.0024	-0.017	0.011
Calcium_UKBB	CA_FinnGen	rs115227958	T	C	-0.0571	0.0053	-0.007	0.032
Calcium_UKBB	CA_FinnGen	rs11588907	T	C	-0.0121	0.0020	-0.010	0.009
Calcium_UKBB	CA_FinnGen	rs115946508	A	C	0.0258	0.0031	0.006	0.017
Calcium_UKBB	CA_FinnGen	rs11621792	T	C	0.0121	0.0020	-0.005	0.009
Calcium_UKBB	CA_FinnGen	rs11629876	T	C	-0.0137	0.0021	0.002	0.010
Calcium_UKBB	CA_FinnGen	rs11671393	C	G	-0.0345	0.0051	0.043	0.021
Calcium_UKBB	CA_FinnGen	rs116769926	A	C	0.0547	0.0064	-0.007	0.048
Calcium_UKBB	CA_FinnGen	rs117080167	T	C	-0.0249	0.0035	-0.005	0.013
Calcium_UKBB	CA_FinnGen	rs117080418	A	T	-0.0740	0.0099	0.054	0.168
Calcium_UKBB	CA_FinnGen	rs117179023	T	C	-0.0536	0.0091	0.033	0.028
Calcium_UKBB	CA_FinnGen	rs117213754	A	G	0.0818	0.0080	-0.049	0.061
Calcium_UKBB	CA_FinnGen	rs11730491	T	G	0.0161	0.0026	-0.031	0.013
Calcium_UKBB	CA_FinnGen	rs117896857	T	C	-0.0410	0.0059	0.024	0.047
Calcium_UKBB	CA_FinnGen	rs11792928	T	C	-0.0126	0.0021	0.015	0.009
Calcium_UKBB	CA_FinnGen	rs12132412	G	A	0.0211	0.0020	0.016	0.009
Calcium_UKBB	CA_FinnGen	rs12135382	T	C	0.0174	0.0020	0.004	0.009
Calcium_UKBB	CA_FinnGen	rs12147703	A	G	-0.0218	0.0032	-0.027	0.013
Calcium_UKBB	CA_FinnGen	rs12339541	A	C	-0.0463	0.0040	0.011	0.018
Calcium_UKBB	CA_FinnGen	rs12378991	A	G	-0.0284	0.0036	-0.012	0.019
Calcium_UKBB	CA_FinnGen	rs12519940	T	C	-0.0201	0.0022	-0.002	0.010
Calcium_UKBB	CA_FinnGen	rs12583851	C	T	-0.0180	0.0022	-0.010	0.009
Calcium_UKBB	CA_FinnGen	rs1260326	C	T	-0.0371	0.0020	-0.003	0.009
Calcium_UKBB	CA_FinnGen	rs12613807	C	T	0.0121	0.0020	-0.007	0.009
Calcium_UKBB	CA_FinnGen	rs12675477	T	C	0.0139	0.0022	-0.013	0.012
Calcium_UKBB	CA_FinnGen	rs12793731	T	C	0.0114	0.0020	0.002	0.009
Calcium_UKBB	CA_FinnGen	rs12918968	C	A	-0.0237	0.0020	0.006	0.009
Calcium_UKBB	CA_FinnGen	rs12922549	T	C	-0.0171	0.0023	-0.013	0.010

Calcium_UKBB	CA_FinnGen	rs12982234	T	C	-0.0452	0.0050	0.055	0.029
Calcium_UKBB	CA_FinnGen	rs12998379	A	G	-0.0180	0.0025	0.007	0.012
Calcium_UKBB	CA_FinnGen	rs13073106	T	C	0.0285	0.0020	0.017	0.009
Calcium_UKBB	CA_FinnGen	rs13107325	T	C	-0.0484	0.0037	0.015	0.037
Calcium_UKBB	CA_FinnGen	rs13108218	G	A	-0.0299	0.0020	-0.002	0.010
Calcium_UKBB	CA_FinnGen	rs13259549	C	T	0.0116	0.0020	-0.026	0.009
Calcium_UKBB	CA_FinnGen	rs13389219	T	C	-0.0129	0.0020	-0.016	0.009
Calcium_UKBB	CA_FinnGen	rs1354034	C	T	-0.0142	0.0020	-0.007	0.010
Calcium_UKBB	CA_FinnGen	rs1374161	A	G	-0.0170	0.0019	-0.010	0.009
Calcium_UKBB	CA_FinnGen	rs138789759	A	G	0.0361	0.0037	-0.026	0.017
Calcium_UKBB	CA_FinnGen	rs147233090	T	C	0.0790	0.0063	0.036	0.061
Calcium_UKBB	CA_FinnGen	rs1476698	G	A	-0.0157	0.0020	-0.010	0.009
Calcium_UKBB	CA_FinnGen	rs148148022	A	G	-0.0539	0.0085	0.003	0.045
Calcium_UKBB	CA_FinnGen	rs1497826	G	C	0.0186	0.0020	-0.011	0.009
Calcium_UKBB	CA_FinnGen	rs149807892	T	C	0.0492	0.0079	0.006	0.064
Calcium_UKBB	CA_FinnGen	rs150268548	A	G	0.0277	0.0038	-0.001	0.018
Calcium_UKBB	CA_FinnGen	rs1544432	G	T	0.0131	0.0022	0.011	0.010
Calcium_UKBB	CA_FinnGen	rs164751	T	G	-0.0146	0.0020	-0.022	0.009
Calcium_UKBB	CA_FinnGen	rs165316	G	A	-0.0139	0.0024	-0.011	0.011
Calcium_UKBB	CA_FinnGen	rs1672991	G	A	0.0571	0.0039	0.011	0.018
Calcium_UKBB	CA_FinnGen	rs17132144	T	C	-0.0189	0.0033	0.013	0.013
Calcium_UKBB	CA_FinnGen	rs17164683	T	C	-0.0164	0.0022	0.010	0.010
Calcium_UKBB	CA_FinnGen	rs17200894	G	C	0.1044	0.0028	-0.022	0.016
Calcium_UKBB	CA_FinnGen	rs17580	A	T	0.0357	0.0045	-0.009	0.047
Calcium_UKBB	CA_FinnGen	rs1763519	C	G	-0.0246	0.0020	0.005	0.009
Calcium_UKBB	CA_FinnGen	rs17774672	A	G	-0.0210	0.0027	-0.006	0.010
Calcium_UKBB	CA_FinnGen	rs17884869	A	G	-0.0849	0.0062	-0.014	0.024
Calcium_UKBB	CA_FinnGen	rs1801282	G	C	-0.0281	0.0030	0.003	0.012
Calcium_UKBB	CA_FinnGen	rs1858800	T	C	0.0233	0.0021	-0.014	0.011
Calcium_UKBB	CA_FinnGen	rs2001884	A	T	-0.0130	0.0019	0.003	0.009
Calcium_UKBB	CA_FinnGen	rs2004315	T	C	0.0253	0.0020	-0.005	0.009
Calcium_UKBB	CA_FinnGen	rs2249825	C	G	-0.0122	0.0022	-0.007	0.009
Calcium_UKBB	CA_FinnGen	rs2274224	C	G	-0.0133	0.0020	-0.014	0.009
Calcium_UKBB	CA_FinnGen	rs2309233	C	T	0.0161	0.0022	0.000	0.010

Calcium_UKBB	CA_FinnGen	rs2327774	C	T	-0.0165	0.0020	-0.008	0.009
Calcium_UKBB	CA_FinnGen	rs2335534	A	G	-0.0298	0.0025	0.026	0.012
Calcium_UKBB	CA_FinnGen	rs2343592	G	A	-0.0178	0.0022	-0.012	0.011
Calcium_UKBB	CA_FinnGen	rs2370218	T	G	-0.0163	0.0023	0.005	0.010
Calcium_UKBB	CA_FinnGen	rs2395158	G	A	-0.0179	0.0026	-0.010	0.014
Calcium_UKBB	CA_FinnGen	rs2419886	T	C	-0.0151	0.0022	-0.006	0.010
Calcium_UKBB	CA_FinnGen	rs2647242	G	A	-0.0154	0.0024	-0.012	0.012
Calcium_UKBB	CA_FinnGen	rs2762938	A	G	0.0119	0.0020	-0.016	0.009
Calcium_UKBB	CA_FinnGen	rs28520334	T	G	0.0165	0.0030	0.012	0.014
Calcium_UKBB	CA_FinnGen	rs28929474	T	C	0.0953	0.0069	-0.005	0.032
Calcium_UKBB	CA_FinnGen	rs2930191	A	G	-0.0158	0.0020	-0.003	0.009
Calcium_UKBB	CA_FinnGen	rs2971855	A	G	0.0142	0.0021	-0.018	0.009
Calcium_UKBB	CA_FinnGen	rs3011642	T	C	0.0148	0.0023	-0.002	0.012
Calcium_UKBB	CA_FinnGen	rs3026445	C	T	-0.0133	0.0020	0.001	0.009
Calcium_UKBB	CA_FinnGen	rs302650	A	G	-0.0143	0.0020	0.009	0.009
Calcium_UKBB	CA_FinnGen	rs3091842	A	G	0.0733	0.0049	0.015	0.016
Calcium_UKBB	CA_FinnGen	rs3133548	T	C	0.0159	0.0028	-0.021	0.013
Calcium_UKBB	CA_FinnGen	rs34290411	G	C	0.0134	0.0021	-0.006	0.011
Calcium_UKBB	CA_FinnGen	rs35118755	T	C	0.0177	0.0027	0.002	0.016
Calcium_UKBB	CA_FinnGen	rs35320690	C	T	0.0195	0.0022	-0.004	0.010
Calcium_UKBB	CA_FinnGen	rs35590487	T	C	-0.0165	0.0023	0.000	0.010
Calcium_UKBB	CA_FinnGen	rs35751693	T	C	0.0326	0.0052	0.026	0.030
Calcium_UKBB	CA_FinnGen	rs35852840	A	C	0.0240	0.0041	-0.015	0.022
Calcium_UKBB	CA_FinnGen	rs36032443	C	A	-0.0190	0.0020	-0.006	0.009
Calcium_UKBB	CA_FinnGen	rs36086195	T	C	0.0154	0.0020	0.007	0.010
Calcium_UKBB	CA_FinnGen	rs36104352	C	A	0.0222	0.0030	0.002	0.018
Calcium_UKBB	CA_FinnGen	rs3748861	A	C	-0.0134	0.0024	0.006	0.011
Calcium_UKBB	CA_FinnGen	rs3794695	T	C	0.0158	0.0025	-0.005	0.011
Calcium_UKBB	CA_FinnGen	rs3795243	C	G	0.0170	0.0029	0.010	0.019
Calcium_UKBB	CA_FinnGen	rs3822858	C	T	-0.0126	0.0020	-0.008	0.010
Calcium_UKBB	CA_FinnGen	rs3931841	G	A	-0.0196	0.0021	-0.003	0.009
Calcium_UKBB	CA_FinnGen	rs4082330	T	C	0.0154	0.0025	0.008	0.014
Calcium_UKBB	CA_FinnGen	rs41278174	A	G	0.0390	0.0059	0.007	0.025
Calcium_UKBB	CA_FinnGen	rs4239142	G	A	-0.0132	0.0022	-0.008	0.010

Calcium_UKBB	CA_FinnGen	rs4320103	G	A	0.0345	0.0051	0.021	0.022
Calcium_UKBB	CA_FinnGen	rs4324076	C	A	-0.0140	0.0019	0.005	0.009
Calcium_UKBB	CA_FinnGen	rs4633480	A	G	0.0151	0.0020	0.007	0.009
Calcium_UKBB	CA_FinnGen	rs4653767	C	T	-0.0150	0.0023	-0.007	0.010
Calcium_UKBB	CA_FinnGen	rs4721467	T	A	-0.0174	0.0022	-0.006	0.011
Calcium_UKBB	CA_FinnGen	rs4744854	C	G	-0.0243	0.0020	-0.005	0.009
Calcium_UKBB	CA_FinnGen	rs4758621	G	A	-0.0204	0.0021	0.006	0.010
Calcium_UKBB	CA_FinnGen	rs4790310	T	C	-0.0148	0.0020	-0.013	0.009
Calcium_UKBB	CA_FinnGen	rs4841132	G	A	0.0425	0.0034	0.030	0.013
Calcium_UKBB	CA_FinnGen	rs4935009	C	A	-0.0172	0.0029	-0.004	0.013
Calcium_UKBB	CA_FinnGen	rs4938642	C	G	0.0254	0.0037	0.030	0.027
Calcium_UKBB	CA_FinnGen	rs498490	T	C	-0.0232	0.0026	-0.007	0.011
Calcium_UKBB	CA_FinnGen	rs55772024	A	G	-0.0133	0.0023	-0.006	0.009
Calcium_UKBB	CA_FinnGen	rs56406311	T	C	0.0134	0.0020	-0.006	0.010
Calcium_UKBB	CA_FinnGen	rs5751350	A	G	0.0127	0.0021	-0.015	0.009
Calcium_UKBB	CA_FinnGen	rs5760495	T	C	0.0123	0.0020	-0.004	0.009
Calcium_UKBB	CA_FinnGen	rs5786388	CA	C	0.0170	0.0020	-0.005	0.009
Calcium_UKBB	CA_FinnGen	rs59821684	G	A	0.0416	0.0058	-0.024	0.045
Calcium_UKBB	CA_FinnGen	rs6127099	T	A	-0.0407	0.0022	-0.003	0.010
Calcium_UKBB	CA_FinnGen	rs62134679	C	T	0.0164	0.0027	-0.004	0.014
Calcium_UKBB	CA_FinnGen	rs62211622	A	C	-0.0141	0.0025	0.025	0.014
Calcium_UKBB	CA_FinnGen	rs62309863	T	G	-0.0114	0.0020	0.008	0.009
Calcium_UKBB	CA_FinnGen	rs62472728	T	C	0.0237	0.0041	-0.029	0.015
Calcium_UKBB	CA_FinnGen	rs634916	T	A	-0.0106	0.0019	-0.018	0.009
Calcium_UKBB	CA_FinnGen	rs6580981	A	G	-0.0137	0.0020	-0.004	0.009
Calcium_UKBB	CA_FinnGen	rs66920316	A	G	-0.0153	0.0024	0.016	0.014
Calcium_UKBB	CA_FinnGen	rs6698689	A	G	-0.0229	0.0030	-0.001	0.015
Calcium_UKBB	CA_FinnGen	rs6741561	T	C	-0.0291	0.0020	-0.001	0.009
Calcium_UKBB	CA_FinnGen	rs6841429	A	C	-0.0300	0.0026	-0.005	0.013
Calcium_UKBB	CA_FinnGen	rs7003580	T	C	0.0123	0.0020	-0.004	0.009
Calcium_UKBB	CA_FinnGen	rs710217	G	A	0.0183	0.0019	0.001	0.009
Calcium_UKBB	CA_FinnGen	rs71565393	T	C	0.0140	0.0026	-0.009	0.013
Calcium_UKBB	CA_FinnGen	rs71658797	A	T	0.0164	0.0030	0.008	0.013
Calcium_UKBB	CA_FinnGen	rs7221118	C	T	-0.0151	0.0024	-0.004	0.010

Calcium_UKBB	CA_FinnGen	rs72697816	T	C	0.0149	0.0026	-0.012	0.011
Calcium_UKBB	CA_FinnGen	rs7370877	G	A	-0.0132	0.0020	0.005	0.009
Calcium_UKBB	CA_FinnGen	rs7402977	A	G	-0.0125	0.0022	-0.001	0.010
Calcium_UKBB	CA_FinnGen	rs74230087	A	G	-0.0422	0.0036	-0.003	0.017
Calcium_UKBB	CA_FinnGen	rs75448233	T	C	0.0253	0.0043	0.016	0.024
Calcium_UKBB	CA_FinnGen	rs7546838	G	A	0.0152	0.0021	0.029	0.009
Calcium_UKBB	CA_FinnGen	rs7559013	C	A	0.0182	0.0029	0.004	0.015
Calcium_UKBB	CA_FinnGen	rs75702986	A	G	0.0277	0.0025	0.001	0.011
Calcium_UKBB	CA_FinnGen	rs7587636	A	G	-0.0127	0.0020	0.000	0.009
Calcium_UKBB	CA_FinnGen	rs75895430	G	C	0.0619	0.0056	-0.048	0.037
Calcium_UKBB	CA_FinnGen	rs7592216	T	G	-0.0158	0.0029	0.032	0.016
Calcium_UKBB	CA_FinnGen	rs7599	G	A	-0.0146	0.0020	-0.006	0.010
Calcium_UKBB	CA_FinnGen	rs76947531	C	T	-0.0689	0.0031	0.011	0.018
Calcium_UKBB	CA_FinnGen	rs7730344	A	C	0.0139	0.0021	0.008	0.009
Calcium_UKBB	CA_FinnGen	rs775249	T	C	-0.0122	0.0022	0.009	0.010
Calcium_UKBB	CA_FinnGen	rs77542162	G	A	-0.0668	0.0065	0.051	0.054
Calcium_UKBB	CA_FinnGen	rs777588	A	G	-0.0223	0.0020	0.016	0.009
Calcium_UKBB	CA_FinnGen	rs778368	C	G	-0.0112	0.0020	-0.011	0.010
Calcium_UKBB	CA_FinnGen	rs7786368	C	T	-0.0187	0.0020	-0.007	0.009
Calcium_UKBB	CA_FinnGen	rs79501693	C	T	-0.0377	0.0068	0.057	0.041
Calcium_UKBB	CA_FinnGen	rs8054061	C	T	0.0146	0.0019	-0.003	0.009
Calcium_UKBB	CA_FinnGen	rs838717	A	G	-0.0354	0.0020	0.002	0.009
Calcium_UKBB	CA_FinnGen	rs883951	G	A	0.0178	0.0022	0.004	0.010
Calcium_UKBB	CA_FinnGen	rs928760	T	C	-0.0142	0.0021	-0.005	0.010
Calcium_UKBB	CA_FinnGen	rs9379881	C	T	-0.0116	0.0019	-0.001	0.009
Calcium_UKBB	CA_FinnGen	rs9388399	C	T	-0.0188	0.0021	0.007	0.009
Calcium_UKBB	CA_FinnGen	rs9399697	T	C	0.0110	0.0019	0.004	0.009
Calcium_UKBB	CA_FinnGen	rs9419741	G	A	0.0118	0.0019	-0.009	0.009
Calcium_UKBB	CA_FinnGen	rs9447004	G	A	-0.0323	0.0019	0.002	0.009
Calcium_UKBB	CA_FinnGen	rs945890	T	A	-0.0130	0.0022	0.021	0.010
Calcium_UKBB	CA_FinnGen	rs948737	T	C	-0.0116	0.0020	-0.004	0.009
Calcium_UKBB	CA_FinnGen	rs949300	A	T	0.0118	0.0020	-0.011	0.009
Calcium_UKBB	CA_FinnGen	rs9530	G	A	-0.0241	0.0019	0.002	0.009
Calcium_UKBB	CA_FinnGen	rs9532958	A	G	0.0264	0.0027	0.006	0.012

Calcium_UKBB	CVD_FinnGen	rs1036332	C	A	0.0166	0.0022	-0.015	0.009
Calcium_UKBB	CVD_FinnGen	rs10513810	G	A	-0.0204	0.0030	0.002	0.011
Calcium_UKBB	CVD_FinnGen	rs10754439	T	G	0.0113	0.0020	0.001	0.008
Calcium_UKBB	CVD_FinnGen	rs10819178	G	T	0.0251	0.0020	-0.001	0.008
Calcium_UKBB	CVD_FinnGen	rs10858935	A	G	-0.0142	0.0021	0.010	0.008
Calcium_UKBB	CVD_FinnGen	rs10917386	T	C	0.0152	0.0021	0.000	0.009
Calcium_UKBB	CVD_FinnGen	rs10958700	G	T	0.0152	0.0023	0.011	0.010
Calcium_UKBB	CVD_FinnGen	rs11078597	C	T	0.0386	0.0025	-0.002	0.010
Calcium_UKBB	CVD_FinnGen	rs11117777	C	T	0.0156	0.0027	-0.010	0.010
Calcium_UKBB	CVD_FinnGen	rs112174050	T	C	0.0804	0.0062	0.003	0.016
Calcium_UKBB	CVD_FinnGen	rs113955164	A	G	0.0158	0.0024	0.020	0.009
Calcium_UKBB	CVD_FinnGen	rs115227958	T	C	-0.0571	0.0053	-0.019	0.028
Calcium_UKBB	CVD_FinnGen	rs11588907	T	C	-0.0121	0.0020	-0.001	0.008
Calcium_UKBB	CVD_FinnGen	rs115946508	A	C	0.0258	0.0031	0.001	0.014
Calcium_UKBB	CVD_FinnGen	rs11621792	T	C	0.0121	0.0020	-0.013	0.008
Calcium_UKBB	CVD_FinnGen	rs11629876	T	C	-0.0137	0.0021	-0.025	0.008
Calcium_UKBB	CVD_FinnGen	rs11671393	C	G	-0.0345	0.0051	-0.037	0.018
Calcium_UKBB	CVD_FinnGen	rs116769926	A	C	0.0547	0.0064	-0.069	0.043
Calcium_UKBB	CVD_FinnGen	rs117080167	T	C	-0.0249	0.0035	-0.018	0.011
Calcium_UKBB	CVD_FinnGen	rs117080418	A	T	-0.0740	0.0099	-0.038	0.149
Calcium_UKBB	CVD_FinnGen	rs117179023	T	C	-0.0536	0.0091	0.018	0.025
Calcium_UKBB	CVD_FinnGen	rs117213754	A	G	0.0818	0.0080	0.015	0.055
Calcium_UKBB	CVD_FinnGen	rs11730491	T	G	0.0161	0.0026	0.009	0.011
Calcium_UKBB	CVD_FinnGen	rs117896857	T	C	-0.0410	0.0059	0.039	0.041
Calcium_UKBB	CVD_FinnGen	rs11792928	T	C	-0.0126	0.0021	0.006	0.008
Calcium_UKBB	CVD_FinnGen	rs12132412	G	A	0.0211	0.0020	0.011	0.008
Calcium_UKBB	CVD_FinnGen	rs12135382	T	C	0.0174	0.0020	-0.012	0.008
Calcium_UKBB	CVD_FinnGen	rs12147703	A	G	-0.0218	0.0032	-0.017	0.011
Calcium_UKBB	CVD_FinnGen	rs12339541	A	C	-0.0463	0.0040	-0.024	0.016
Calcium_UKBB	CVD_FinnGen	rs12378991	A	G	-0.0284	0.0036	0.014	0.016
Calcium_UKBB	CVD_FinnGen	rs12519940	T	C	-0.0201	0.0022	-0.002	0.009
Calcium_UKBB	CVD_FinnGen	rs12583851	C	T	-0.0180	0.0022	0.004	0.008
Calcium_UKBB	CVD_FinnGen	rs1260326	C	T	-0.0371	0.0020	-0.006	0.008
Calcium_UKBB	CVD_FinnGen	rs12613807	C	T	0.0121	0.0020	0.000	0.008

Calcium_UKBB	CVD_FinnGen	rs12675477	T	C	0.0139	0.0022	0.008	0.010
Calcium_UKBB	CVD_FinnGen	rs12793731	T	C	0.0114	0.0020	0.008	0.008
Calcium_UKBB	CVD_FinnGen	rs12918968	C	A	-0.0237	0.0020	-0.023	0.008
Calcium_UKBB	CVD_FinnGen	rs12922549	T	C	-0.0171	0.0023	0.003	0.009
Calcium_UKBB	CVD_FinnGen	rs12982234	T	C	-0.0452	0.0050	-0.003	0.025
Calcium_UKBB	CVD_FinnGen	rs12998379	A	G	-0.0180	0.0025	-0.013	0.010
Calcium_UKBB	CVD_FinnGen	rs13073106	T	C	0.0285	0.0020	0.011	0.008
Calcium_UKBB	CVD_FinnGen	rs13107325	T	C	-0.0484	0.0037	0.008	0.033
Calcium_UKBB	CVD_FinnGen	rs13108218	G	A	-0.0299	0.0020	-0.016	0.008
Calcium_UKBB	CVD_FinnGen	rs13259549	C	T	0.0116	0.0020	-0.016	0.008
Calcium_UKBB	CVD_FinnGen	rs13389219	T	C	-0.0129	0.0020	-0.020	0.008
Calcium_UKBB	CVD_FinnGen	rs1354034	C	T	-0.0142	0.0020	-0.005	0.009
Calcium_UKBB	CVD_FinnGen	rs1374161	A	G	-0.0170	0.0019	0.003	0.008
Calcium_UKBB	CVD_FinnGen	rs138789759	A	G	0.0361	0.0037	-0.001	0.014
Calcium_UKBB	CVD_FinnGen	rs147233090	T	C	0.0790	0.0063	0.036	0.054
Calcium_UKBB	CVD_FinnGen	rs1476698	G	A	-0.0157	0.0020	0.001	0.008
Calcium_UKBB	CVD_FinnGen	rs148148022	A	G	-0.0539	0.0085	-0.073	0.038
Calcium_UKBB	CVD_FinnGen	rs1497826	G	C	0.0186	0.0020	0.000	0.008
Calcium_UKBB	CVD_FinnGen	rs149807892	T	C	0.0492	0.0079	0.005	0.056
Calcium_UKBB	CVD_FinnGen	rs150268548	A	G	0.0277	0.0038	-0.007	0.016
Calcium_UKBB	CVD_FinnGen	rs1544432	G	T	0.0131	0.0022	-0.016	0.009
Calcium_UKBB	CVD_FinnGen	rs164751	T	G	-0.0146	0.0020	-0.011	0.008
Calcium_UKBB	CVD_FinnGen	rs165316	G	A	-0.0139	0.0024	-0.028	0.009
Calcium_UKBB	CVD_FinnGen	rs1672991	G	A	0.0571	0.0039	0.001	0.016
Calcium_UKBB	CVD_FinnGen	rs17132144	T	C	-0.0189	0.0033	0.010	0.012
Calcium_UKBB	CVD_FinnGen	rs17164683	T	C	-0.0164	0.0022	-0.003	0.009
Calcium_UKBB	CVD_FinnGen	rs17200894	G	C	0.1044	0.0028	0.015	0.014
Calcium_UKBB	CVD_FinnGen	rs17580	A	T	0.0357	0.0045	-0.014	0.041
Calcium_UKBB	CVD_FinnGen	rs1763519	C	G	-0.0246	0.0020	0.004	0.008
Calcium_UKBB	CVD_FinnGen	rs17774672	A	G	-0.0210	0.0027	-0.011	0.009
Calcium_UKBB	CVD_FinnGen	rs17884869	A	G	-0.0849	0.0062	0.020	0.021
Calcium_UKBB	CVD_FinnGen	rs1801282	G	C	-0.0281	0.0030	-0.015	0.010
Calcium_UKBB	CVD_FinnGen	rs1858800	T	C	0.0233	0.0021	0.004	0.009
Calcium_UKBB	CVD_FinnGen	rs2001884	A	T	-0.0130	0.0019	-0.007	0.008

Calcium_UKBB	CVD_FinnGen	rs2004315	T	C	0.0253	0.0020	0.010	0.008
Calcium_UKBB	CVD_FinnGen	rs2249825	C	G	-0.0122	0.0022	0.002	0.008
Calcium_UKBB	CVD_FinnGen	rs2274224	C	G	-0.0133	0.0020	-0.025	0.008
Calcium_UKBB	CVD_FinnGen	rs2309233	C	T	0.0161	0.0022	0.014	0.009
Calcium_UKBB	CVD_FinnGen	rs2327774	C	T	-0.0165	0.0020	0.002	0.008
Calcium_UKBB	CVD_FinnGen	rs2335534	A	G	-0.0298	0.0025	-0.019	0.010
Calcium_UKBB	CVD_FinnGen	rs2343592	G	A	-0.0178	0.0022	-0.002	0.009
Calcium_UKBB	CVD_FinnGen	rs2370218	T	G	-0.0163	0.0023	0.010	0.008
Calcium_UKBB	CVD_FinnGen	rs2395158	G	A	-0.0179	0.0026	0.001	0.012
Calcium_UKBB	CVD_FinnGen	rs2419886	T	C	-0.0151	0.0022	0.014	0.009
Calcium_UKBB	CVD_FinnGen	rs2647242	G	A	-0.0154	0.0024	-0.007	0.010
Calcium_UKBB	CVD_FinnGen	rs2762938	A	G	0.0119	0.0020	0.000	0.008
Calcium_UKBB	CVD_FinnGen	rs28520334	T	G	0.0165	0.0030	-0.014	0.013
Calcium_UKBB	CVD_FinnGen	rs28929474	T	C	0.0953	0.0069	0.014	0.028
Calcium_UKBB	CVD_FinnGen	rs2930191	A	G	-0.0158	0.0020	-0.006	0.008
Calcium_UKBB	CVD_FinnGen	rs2971855	A	G	0.0142	0.0021	-0.017	0.008
Calcium_UKBB	CVD_FinnGen	rs3011642	T	C	0.0148	0.0023	0.008	0.010
Calcium_UKBB	CVD_FinnGen	rs3026445	C	T	-0.0133	0.0020	-0.017	0.008
Calcium_UKBB	CVD_FinnGen	rs302650	A	G	-0.0143	0.0020	-0.002	0.008
Calcium_UKBB	CVD_FinnGen	rs3091842	A	G	0.0733	0.0049	0.009	0.014
Calcium_UKBB	CVD_FinnGen	rs3133548	T	C	0.0159	0.0028	-0.004	0.011
Calcium_UKBB	CVD_FinnGen	rs34290411	G	C	0.0134	0.0021	0.001	0.009
Calcium_UKBB	CVD_FinnGen	rs35118755	T	C	0.0177	0.0027	-0.021	0.014
Calcium_UKBB	CVD_FinnGen	rs35320690	C	T	0.0195	0.0022	-0.006	0.009
Calcium_UKBB	CVD_FinnGen	rs35590487	T	C	-0.0165	0.0023	0.001	0.009
Calcium_UKBB	CVD_FinnGen	rs35751693	T	C	0.0326	0.0052	0.020	0.026
Calcium_UKBB	CVD_FinnGen	rs35852840	A	C	0.0240	0.0041	-0.021	0.019
Calcium_UKBB	CVD_FinnGen	rs36032443	C	A	-0.0190	0.0020	0.008	0.008
Calcium_UKBB	CVD_FinnGen	rs36086195	T	C	0.0154	0.0020	0.010	0.008
Calcium_UKBB	CVD_FinnGen	rs36104352	C	A	0.0222	0.0030	0.003	0.015
Calcium_UKBB	CVD_FinnGen	rs3748861	A	C	-0.0134	0.0024	0.004	0.010
Calcium_UKBB	CVD_FinnGen	rs3794695	T	C	0.0158	0.0025	0.008	0.010
Calcium_UKBB	CVD_FinnGen	rs3795243	C	G	0.0170	0.0029	0.004	0.017
Calcium_UKBB	CVD_FinnGen	rs3822858	C	T	-0.0126	0.0020	0.006	0.008

Calcium_UKBB	CVD_FinnGen	rs3931841	G	A	-0.0196	0.0021	-0.011	0.008
Calcium_UKBB	CVD_FinnGen	rs4082330	T	C	0.0154	0.0025	-0.003	0.012
Calcium_UKBB	CVD_FinnGen	rs41278174	A	G	0.0390	0.0059	-0.005	0.021
Calcium_UKBB	CVD_FinnGen	rs4239142	G	A	-0.0132	0.0022	-0.014	0.009
Calcium_UKBB	CVD_FinnGen	rs4320103	G	A	0.0345	0.0051	-0.017	0.019
Calcium_UKBB	CVD_FinnGen	rs4324076	C	A	-0.0140	0.0019	0.013	0.008
Calcium_UKBB	CVD_FinnGen	rs4633480	A	G	0.0151	0.0020	0.006	0.008
Calcium_UKBB	CVD_FinnGen	rs4653767	C	T	-0.0150	0.0023	0.002	0.009
Calcium_UKBB	CVD_FinnGen	rs4721467	T	A	-0.0174	0.0022	0.010	0.010
Calcium_UKBB	CVD_FinnGen	rs4744854	C	G	-0.0243	0.0020	0.005	0.008
Calcium_UKBB	CVD_FinnGen	rs4758621	G	A	-0.0204	0.0021	-0.016	0.009
Calcium_UKBB	CVD_FinnGen	rs4790310	T	C	-0.0148	0.0020	0.008	0.008
Calcium_UKBB	CVD_FinnGen	rs4841132	G	A	0.0425	0.0034	0.002	0.012
Calcium_UKBB	CVD_FinnGen	rs4935009	C	A	-0.0172	0.0029	0.003	0.011
Calcium_UKBB	CVD_FinnGen	rs4938642	C	G	0.0254	0.0037	-0.020	0.024
Calcium_UKBB	CVD_FinnGen	rs498490	T	C	-0.0232	0.0026	-0.003	0.009
Calcium_UKBB	CVD_FinnGen	rs55772024	A	G	-0.0133	0.0023	0.002	0.008
Calcium_UKBB	CVD_FinnGen	rs56406311	T	C	0.0134	0.0020	-0.023	0.008
Calcium_UKBB	CVD_FinnGen	rs5751350	A	G	0.0127	0.0021	0.015	0.008
Calcium_UKBB	CVD_FinnGen	rs5760495	T	C	0.0123	0.0020	0.009	0.008
Calcium_UKBB	CVD_FinnGen	rs5786388	CA	C	0.0170	0.0020	-0.003	0.008
Calcium_UKBB	CVD_FinnGen	rs59821684	G	A	0.0416	0.0058	-0.004	0.040
Calcium_UKBB	CVD_FinnGen	rs6127099	T	A	-0.0407	0.0022	-0.012	0.009
Calcium_UKBB	CVD_FinnGen	rs62134679	C	T	0.0164	0.0027	0.008	0.012
Calcium_UKBB	CVD_FinnGen	rs62211622	A	C	-0.0141	0.0025	-0.021	0.012
Calcium_UKBB	CVD_FinnGen	rs62309863	T	G	-0.0114	0.0020	-0.008	0.008
Calcium_UKBB	CVD_FinnGen	rs62472728	T	C	0.0237	0.0041	0.015	0.013
Calcium_UKBB	CVD_FinnGen	rs634916	T	A	-0.0106	0.0019	-0.006	0.008
Calcium_UKBB	CVD_FinnGen	rs6580981	A	G	-0.0137	0.0020	0.000	0.008
Calcium_UKBB	CVD_FinnGen	rs66920316	A	G	-0.0153	0.0024	-0.007	0.012
Calcium_UKBB	CVD_FinnGen	rs6698689	A	G	-0.0229	0.0030	0.008	0.013
Calcium_UKBB	CVD_FinnGen	rs6741561	T	C	-0.0291	0.0020	-0.003	0.008
Calcium_UKBB	CVD_FinnGen	rs6841429	A	C	-0.0300	0.0026	0.003	0.012
Calcium_UKBB	CVD_FinnGen	rs7003580	T	C	0.0123	0.0020	0.003	0.008

Calcium_UKBB	CVD_FinnGen	rs710217	G	A	0.0183	0.0019	-0.005	0.008
Calcium_UKBB	CVD_FinnGen	rs71565393	T	C	0.0140	0.0026	-0.005	0.011
Calcium_UKBB	CVD_FinnGen	rs71658797	A	T	0.0164	0.0030	0.014	0.012
Calcium_UKBB	CVD_FinnGen	rs7221118	C	T	-0.0151	0.0024	0.002	0.008
Calcium_UKBB	CVD_FinnGen	rs72697816	T	C	0.0149	0.0026	0.001	0.009
Calcium_UKBB	CVD_FinnGen	rs7370877	G	A	-0.0132	0.0020	0.006	0.008
Calcium_UKBB	CVD_FinnGen	rs7402977	A	G	-0.0125	0.0022	-0.016	0.009
Calcium_UKBB	CVD_FinnGen	rs74230087	A	G	-0.0422	0.0036	-0.016	0.015
Calcium_UKBB	CVD_FinnGen	rs75448233	T	C	0.0253	0.0043	0.026	0.021
Calcium_UKBB	CVD_FinnGen	rs7546838	G	A	0.0152	0.0021	-0.001	0.008
Calcium_UKBB	CVD_FinnGen	rs7559013	C	A	0.0182	0.0029	0.004	0.013
Calcium_UKBB	CVD_FinnGen	rs75702986	A	G	0.0277	0.0025	0.010	0.010
Calcium_UKBB	CVD_FinnGen	rs7587636	A	G	-0.0127	0.0020	0.020	0.008
Calcium_UKBB	CVD_FinnGen	rs75895430	G	C	0.0619	0.0056	0.013	0.032
Calcium_UKBB	CVD_FinnGen	rs7592216	T	G	-0.0158	0.0029	0.009	0.014
Calcium_UKBB	CVD_FinnGen	rs7599	G	A	-0.0146	0.0020	0.005	0.008
Calcium_UKBB	CVD_FinnGen	rs76947531	C	T	-0.0689	0.0031	0.001	0.016
Calcium_UKBB	CVD_FinnGen	rs7730344	A	C	0.0139	0.0021	-0.010	0.008
Calcium_UKBB	CVD_FinnGen	rs775249	T	C	-0.0122	0.0022	0.016	0.008
Calcium_UKBB	CVD_FinnGen	rs77542162	G	A	-0.0668	0.0065	0.016	0.048
Calcium_UKBB	CVD_FinnGen	rs777588	A	G	-0.0223	0.0020	-0.003	0.008
Calcium_UKBB	CVD_FinnGen	rs778368	C	G	-0.0112	0.0020	-0.016	0.009
Calcium_UKBB	CVD_FinnGen	rs7786368	C	T	-0.0187	0.0020	-0.004	0.008
Calcium_UKBB	CVD_FinnGen	rs79501693	C	T	-0.0377	0.0068	0.047	0.036
Calcium_UKBB	CVD_FinnGen	rs8054061	C	T	0.0146	0.0019	0.025	0.008
Calcium_UKBB	CVD_FinnGen	rs838717	A	G	-0.0354	0.0020	-0.014	0.008
Calcium_UKBB	CVD_FinnGen	rs883951	G	A	0.0178	0.0022	0.011	0.008
Calcium_UKBB	CVD_FinnGen	rs928760	T	C	-0.0142	0.0021	0.020	0.009
Calcium_UKBB	CVD_FinnGen	rs9379881	C	T	-0.0116	0.0019	0.008	0.008
Calcium_UKBB	CVD_FinnGen	rs9388399	C	T	-0.0188	0.0021	0.009	0.008
Calcium_UKBB	CVD_FinnGen	rs9399697	T	C	0.0110	0.0019	-0.010	0.008
Calcium_UKBB	CVD_FinnGen	rs9419741	G	A	0.0118	0.0019	0.000	0.008
Calcium_UKBB	CVD_FinnGen	rs9447004	G	A	-0.0323	0.0019	-0.010	0.008
Calcium_UKBB	CVD_FinnGen	rs945890	T	A	-0.0130	0.0022	0.003	0.009

Calcium_UKBB	CVD_FinnGen	rs948737	T	C	-0.0116	0.0020	0.004	0.008
Calcium_UKBB	CVD_FinnGen	rs949300	A	T	0.0118	0.0020	0.006	0.008
Calcium_UKBB	CVD_FinnGen	rs9530	G	A	-0.0241	0.0019	-0.008	0.008
Calcium_UKBB	CVD_FinnGen	rs9532958	A	G	0.0264	0.0027	0.005	0.010
Calcium_UKBB	Longevity_90%	rs36086195	T	C	0.0018	0.0002	-0.034	0.020
Calcium_UKBB	Longevity_90%	rs12132412	G	A	0.0025	0.0002	0.004	0.022
Calcium_UKBB	Longevity_90%	rs10917386	T	C	0.0018	0.0003	0.000	0.021
Calcium_UKBB	Longevity_90%	rs710217	G	A	0.0022	0.0002	-0.010	0.019
Calcium_UKBB	Longevity_90%	rs112174050	T	C	0.0096	0.0007	-0.087	0.087
Calcium_UKBB	Longevity_90%	rs75895430	G	C	0.0074	0.0007	-0.041	0.079
Calcium_UKBB	Longevity_90%	rs71658797	A	T	0.0020	0.0004	0.041	0.032
Calcium_UKBB	Longevity_90%	rs165316	A	G	0.0017	0.0003	-0.027	0.025
Calcium_UKBB	Longevity_90%	rs72697816	T	C	0.0018	0.0003	0.026	0.026
Calcium_UKBB	Longevity_90%	rs10754439	T	G	0.0014	0.0002	-0.006	0.019
Calcium_UKBB	Longevity_90%	rs80350997	A	G	0.0039	0.0004	0.014	0.037
Calcium_UKBB	Longevity_90%	rs12743084	C	G	0.0024	0.0002	0.022	0.021
Calcium_UKBB	Longevity_90%	rs7546838	G	A	0.0018	0.0002	0.007	0.020
Calcium_UKBB	Longevity_90%	rs11588907	C	T	0.0015	0.0002	-0.001	0.020
Calcium_UKBB	Longevity_90%	rs1036332	C	A	0.0020	0.0003	0.010	0.022
Calcium_UKBB	Longevity_90%	rs11117777	C	T	0.0019	0.0003	0.042	0.027
Calcium_UKBB	Longevity_90%	rs1497826	G	C	0.0022	0.0002	0.021	0.020
Calcium_UKBB	Longevity_90%	rs6698689	G	A	0.0028	0.0004	0.041	0.031
Calcium_UKBB	Longevity_90%	rs4653767	T	C	0.0018	0.0003	-0.016	0.021
Calcium_UKBB	Longevity_90%	rs66920316	G	A	0.0018	0.0003	0.036	0.025
Calcium_UKBB	Longevity_90%	rs12998379	G	A	0.0022	0.0003	-0.037	0.029
Calcium_UKBB	Longevity_90%	rs7587636	G	A	0.0015	0.0002	0.008	0.020
Calcium_UKBB	Longevity_90%	rs1260326	T	C	0.0045	0.0002	-0.029	0.020
Calcium_UKBB	Longevity_90%	rs62134679	C	T	0.0020	0.0003	0.026	0.026
Calcium_UKBB	Longevity_90%	rs777588	G	A	0.0027	0.0002	0.035	0.020
Calcium_UKBB	Longevity_90%	rs7370877	A	G	0.0016	0.0002	0.011	0.020
Calcium_UKBB	Longevity_90%	rs6741561	C	T	0.0035	0.0002	0.031	0.020
Calcium_UKBB	Longevity_90%	rs35751693	T	C	0.0039	0.0006	0.120	0.067
Calcium_UKBB	Longevity_90%	rs1374161	G	A	0.0020	0.0002	-0.037	0.020
Calcium_UKBB	Longevity_90%	rs3931841	A	G	0.0024	0.0003	-0.004	0.021

Calcium_UKBB	Longevity_90%	rs3748861	C	A	0.0016	0.0003	0.016	0.026
Calcium_UKBB	Longevity_90%	rs13389219	C	T	0.0015	0.0002	0.006	0.020
Calcium_UKBB	Longevity_90%	rs7592216	G	T	0.0019	0.0003	-0.049	0.029
Calcium_UKBB	Longevity_90%	rs12613807	C	T	0.0015	0.0002	-0.012	0.019
Calcium_UKBB	Longevity_90%	rs7559013	C	A	0.0022	0.0003	0.006	0.029
Calcium_UKBB	Longevity_90%	rs778368	G	C	0.0013	0.0002	-0.018	0.020
Calcium_UKBB	Longevity_90%	rs838717	G	A	0.0042	0.0002	-0.009	0.019
Calcium_UKBB	Longevity_90%	rs2971855	A	G	0.0017	0.0003	0.016	0.021
Calcium_UKBB	Longevity_90%	rs1476698	A	G	0.0019	0.0002	-0.013	0.020
Calcium_UKBB	Longevity_90%	rs28520334	T	G	0.0020	0.0004	-0.016	0.032
Calcium_UKBB	Longevity_90%	rs1801282	C	G	0.0034	0.0004	-0.012	0.029
Calcium_UKBB	Longevity_90%	rs1354034	T	C	0.0017	0.0002	-0.032	0.020
Calcium_UKBB	Longevity_90%	rs138789759	A	G	0.0043	0.0004	-0.058	0.034
Calcium_UKBB	Longevity_90%	rs115227958	C	T	0.0069	0.0006	0.037	0.060
Calcium_UKBB	Longevity_90%	rs76947531	T	C	0.0083	0.0004	0.056	0.034
Calcium_UKBB	Longevity_90%	rs17200894	G	C	0.0125	0.0003	-0.071	0.027
Calcium_UKBB	Longevity_90%	rs35320690	C	T	0.0023	0.0003	-0.009	0.022
Calcium_UKBB	Longevity_90%	rs56406311	T	C	0.0016	0.0002	0.007	0.021
Calcium_UKBB	Longevity_90%	rs13073106	T	C	0.0034	0.0002	-0.024	0.020
Calcium_UKBB	Longevity_90%	rs10513810	A	G	0.0024	0.0004	-0.003	0.030
Calcium_UKBB	Longevity_90%	rs13108218	A	G	0.0036	0.0002	-0.040	0.022
Calcium_UKBB	Longevity_90%	rs3795243	C	G	0.0020	0.0004	0.012	0.030
Calcium_UKBB	Longevity_90%	rs11730491	T	G	0.0019	0.0003	-0.019	0.026
Calcium_UKBB	Longevity_90%	rs6841429	C	A	0.0036	0.0003	-0.003	0.026
Calcium_UKBB	Longevity_90%	rs13107325	C	T	0.0058	0.0004	0.130	0.040
Calcium_UKBB	Longevity_90%	rs2647242	A	G	0.0018	0.0003	-0.014	0.024
Calcium_UKBB	Longevity_90%	rs62309863	G	T	0.0014	0.0002	0.025	0.020
Calcium_UKBB	Longevity_90%	rs4320103	G	A	0.0041	0.0006	-0.030	0.049
Calcium_UKBB	Longevity_90%	rs7730344	A	C	0.0017	0.0003	-0.039	0.021
Calcium_UKBB	Longevity_90%	rs17132144	C	T	0.0023	0.0004	-0.015	0.034
Calcium_UKBB	Longevity_90%	rs12519940	C	T	0.0024	0.0003	0.017	0.022
Calcium_UKBB	Longevity_90%	rs113955164	A	G	0.0019	0.0003	0.012	0.026
Calcium_UKBB	Longevity_90%	rs9379881	T	C	0.0014	0.0002	-0.012	0.019
Calcium_UKBB	Longevity_90%	rs2395158	A	G	0.0021	0.0003	0.035	0.038

Calcium_UKBB	Longevity_90%	rs71565393	T	C	0.0017	0.0003	0.029	0.026
Calcium_UKBB	Longevity_90%	rs55772024	G	A	0.0016	0.0003	0.008	0.022
Calcium_UKBB	Longevity_90%	rs9447004	A	G	0.0039	0.0002	0.032	0.019
Calcium_UKBB	Longevity_90%	rs9399697	T	C	0.0013	0.0002	0.008	0.019
Calcium_UKBB	Longevity_90%	rs3822858	T	C	0.0015	0.0002	-0.027	0.020
Calcium_UKBB	Longevity_90%	rs9388399	T	C	0.0023	0.0003	0.027	0.022
Calcium_UKBB	Longevity_90%	rs945890	A	T	0.0016	0.0003	0.028	0.022
Calcium_UKBB	Longevity_90%	rs1763519	G	C	0.0030	0.0002	0.017	0.021
Calcium_UKBB	Longevity_90%	rs2327774	T	C	0.0020	0.0002	-0.032	0.026
Calcium_UKBB	Longevity_90%	rs634916	A	T	0.0013	0.0002	-0.029	0.019
Calcium_UKBB	Longevity_90%	rs4721467	A	T	0.0021	0.0003	0.023	0.022
Calcium_UKBB	Longevity_90%	rs9530	A	G	0.0029	0.0002	-0.019	0.019
Calcium_UKBB	Longevity_90%	rs7786368	T	C	0.0022	0.0002	-0.006	0.019
Calcium_UKBB	Longevity_90%	rs17164683	C	T	0.0020	0.0003	-0.039	0.022
Calcium_UKBB	Longevity_90%	rs257380	T	A	0.0017	0.0002	0.001	0.021
Calcium_UKBB	Longevity_90%	rs62472728	T	C	0.0028	0.0005	-0.040	0.041
Calcium_UKBB	Longevity_90%	rs115946508	A	C	0.0031	0.0004	0.081	0.031
Calcium_UKBB	Longevity_90%	rs4841132	G	A	0.0051	0.0004	-0.003	0.035
Calcium_UKBB	Longevity_90%	rs2309233	C	T	0.0019	0.0003	0.036	0.024
Calcium_UKBB	Longevity_90%	rs36104352	C	A	0.0027	0.0004	-0.049	0.031
Calcium_UKBB	Longevity_90%	rs10958700	G	T	0.0018	0.0003	-0.020	0.024
Calcium_UKBB	Longevity_90%	rs12675477	T	C	0.0017	0.0003	-0.018	0.022
Calcium_UKBB	Longevity_90%	rs3133548	T	C	0.0019	0.0003	-0.025	0.028
Calcium_UKBB	Longevity_90%	rs2343592	A	G	0.0021	0.0003	-0.020	0.022
Calcium_UKBB	Longevity_90%	rs13259549	C	T	0.0014	0.0002	0.014	0.020
Calcium_UKBB	Longevity_90%	rs7003580	T	C	0.0015	0.0002	0.016	0.021
Calcium_UKBB	Longevity_90%	rs2370218	G	T	0.0020	0.0003	-0.039	0.023
Calcium_UKBB	Longevity_90%	rs883951	G	A	0.0021	0.0003	0.021	0.022
Calcium_UKBB	Longevity_90%	rs12378991	G	A	0.0034	0.0004	0.004	0.038
Calcium_UKBB	Longevity_90%	rs4744854	G	C	0.0029	0.0002	0.060	0.021
Calcium_UKBB	Longevity_90%	rs62575977	T	C	0.0018	0.0002	0.015	0.020
Calcium_UKBB	Longevity_90%	rs12339541	C	A	0.0056	0.0005	-0.055	0.040
Calcium_UKBB	Longevity_90%	rs10819178	G	T	0.0030	0.0002	-0.030	0.020
Calcium_UKBB	Longevity_90%	rs11792928	C	T	0.0015	0.0003	-0.028	0.022

Calcium_UKBB	Longevity_90%	rs498490	C	T	0.0028	0.0003	-0.041	0.025
Calcium_UKBB	Longevity_90%	rs117179023	C	T	0.0064	0.0011	0.088	0.092
Calcium_UKBB	Longevity_90%	rs74230087	G	A	0.0051	0.0004	-0.022	0.037
Calcium_UKBB	Longevity_90%	rs112371897	T	C	0.0064	0.0004	-0.016	0.033
Calcium_UKBB	Longevity_90%	rs3011642	T	C	0.0018	0.0003	0.023	0.023
Calcium_UKBB	Longevity_90%	rs17774672	G	A	0.0025	0.0003	-0.010	0.026
Calcium_UKBB	Longevity_90%	rs4935009	A	C	0.0021	0.0003	0.086	0.028
Calcium_UKBB	Longevity_90%	rs4082330	T	C	0.0018	0.0003	-0.031	0.028
Calcium_UKBB	Longevity_90%	rs9419741	G	A	0.0014	0.0002	-0.039	0.020
Calcium_UKBB	Longevity_90%	rs2274224	G	C	0.0016	0.0002	-0.016	0.020
Calcium_UKBB	Longevity_90%	rs2419886	C	T	0.0018	0.0003	-0.028	0.023
Calcium_UKBB	Longevity_90%	rs4758621	A	G	0.0025	0.0003	0.009	0.022
Calcium_UKBB	Longevity_90%	rs2004315	T	C	0.0030	0.0002	-0.019	0.020
Calcium_UKBB	Longevity_90%	rs2930191	G	A	0.0019	0.0002	-0.039	0.022
Calcium_UKBB	Longevity_90%	rs4633480	A	G	0.0018	0.0002	0.003	0.019
Calcium_UKBB	Longevity_90%	rs12793731	T	C	0.0014	0.0002	0.016	0.030
Calcium_UKBB	Longevity_90%	rs302650	G	A	0.0017	0.0002	0.006	0.019
Calcium_UKBB	Longevity_90%	rs948737	C	T	0.0014	0.0002	0.012	0.021
Calcium_UKBB	Longevity_90%	rs141949189	T	A	0.0014	0.0002	-0.007	0.028
Calcium_UKBB	Longevity_90%	rs4938642	C	G	0.0030	0.0004	-0.007	0.038
Calcium_UKBB	Longevity_90%	rs949300	A	T	0.0014	0.0002	-0.011	0.020
Calcium_UKBB	Longevity_90%	rs73632745	C	T	0.0055	0.0004	0.058	0.036
Calcium_UKBB	Longevity_90%	rs117213754	A	G	0.0098	0.0010	-0.040	0.091
Calcium_UKBB	Longevity_90%	rs117080167	C	T	0.0030	0.0004	0.062	0.035
Calcium_UKBB	Longevity_90%	rs6580981	G	A	0.0016	0.0002	0.011	0.019
Calcium_UKBB	Longevity_90%	rs775249	C	T	0.0015	0.0003	0.000	0.022
Calcium_UKBB	Longevity_90%	rs10858935	G	A	0.0017	0.0003	0.018	0.022
Calcium_UKBB	Longevity_90%	rs3026445	T	C	0.0016	0.0002	-0.037	0.020
Calcium_UKBB	Longevity_90%	rs2249825	G	C	0.0015	0.0003	-0.014	0.023
Calcium_UKBB	Longevity_90%	rs9532958	A	G	0.0032	0.0003	-0.018	0.028
Calcium_UKBB	Longevity_90%	rs12583851	T	C	0.0022	0.0003	-0.033	0.022
Calcium_UKBB	Longevity_90%	rs11621792	T	C	0.0015	0.0002	-0.035	0.021
Calcium_UKBB	Longevity_90%	rs35852840	A	C	0.0029	0.0005	-0.002	0.045
Calcium_UKBB	Longevity_90%	rs12147703	G	A	0.0026	0.0004	-0.031	0.031

Calcium_UKBB	Longevity_90%	rs28929474	T	C	0.0114	0.0008	-0.061	0.076
Calcium_UKBB	Longevity_90%	rs17580	A	T	0.0043	0.0005	-0.065	0.051
Calcium_UKBB	Longevity_90%	rs35590487	C	T	0.0020	0.0003	0.011	0.027
Calcium_UKBB	Longevity_90%	rs7402977	G	A	0.0015	0.0003	-0.018	0.022
Calcium_UKBB	Longevity_90%	rs147233090	T	C	0.0095	0.0008	0.143	0.061
Calcium_UKBB	Longevity_90%	rs4324076	A	C	0.0017	0.0002	-0.018	0.019
Calcium_UKBB	Longevity_90%	rs36032443	A	C	0.0023	0.0002	0.022	0.020
Calcium_UKBB	Longevity_90%	rs60616569	G	A	0.0017	0.0003	-0.033	0.034
Calcium_UKBB	Longevity_90%	rs11629876	C	T	0.0016	0.0002	-0.049	0.020
Calcium_UKBB	Longevity_90%	rs41278174	A	G	0.0047	0.0007	0.003	0.078
Calcium_UKBB	Longevity_90%	rs12922549	C	T	0.0021	0.0003	0.012	0.025
Calcium_UKBB	Longevity_90%	rs3794695	T	C	0.0019	0.0003	0.005	0.025
Calcium_UKBB	Longevity_90%	rs1858800	T	C	0.0028	0.0002	0.033	0.020
Calcium_UKBB	Longevity_90%	rs8054061	C	T	0.0018	0.0002	-0.013	0.019
Calcium_UKBB	Longevity_90%	rs12918968	A	C	0.0028	0.0002	0.016	0.022
Calcium_UKBB	Longevity_90%	rs11078597	C	T	0.0046	0.0003	-0.007	0.028
Calcium_UKBB	Longevity_90%	rs4790310	C	T	0.0018	0.0002	0.017	0.020
Calcium_UKBB	Longevity_90%	rs75448233	T	C	0.0030	0.0005	0.113	0.051
Calcium_UKBB	Longevity_90%	rs2001884	T	A	0.0016	0.0002	0.012	0.019
Calcium_UKBB	Longevity_90%	rs4239142	A	G	0.0016	0.0003	0.008	0.022
Calcium_UKBB	Longevity_90%	rs7221118	T	C	0.0018	0.0003	-0.018	0.024
Calcium_UKBB	Longevity_90%	rs77704893	C	T	0.0039	0.0005	0.018	0.035
Calcium_UKBB	Longevity_90%	rs62071306	A	C	0.0021	0.0003	0.041	0.023
Calcium_UKBB	Longevity_90%	rs77542162	A	G	0.0080	0.0008	-0.016	0.077
Calcium_UKBB	Longevity_90%	rs34290411	G	C	0.0016	0.0003	-0.001	0.022
Calcium_UKBB	Longevity_90%	rs150268548	A	G	0.0033	0.0005	-0.026	0.044
Calcium_UKBB	Longevity_90%	rs1672991	G	A	0.0068	0.0005	0.018	0.040
Calcium_UKBB	Longevity_90%	rs75702986	A	G	0.0033	0.0003	0.028	0.027
Calcium_UKBB	Longevity_90%	rs7599	A	G	0.0018	0.0002	-0.014	0.020
Calcium_UKBB	Longevity_90%	rs12982234	C	T	0.0054	0.0006	0.007	0.060
Calcium_UKBB	Longevity_90%	rs2335534	G	A	0.0036	0.0003	0.018	0.025
Calcium_UKBB	Longevity_90%	rs149807892	T	C	0.0059	0.0009	-0.214	0.107
Calcium_UKBB	Longevity_90%	rs79501693	T	C	0.0045	0.0008	0.044	0.077
Calcium_UKBB	Longevity_90%	rs35118755	T	C	0.0021	0.0003	-0.023	0.030

Calcium_UKBB	Longevity_90%	rs59821684	G	A	0.0050	0.0007	-0.018	0.065
Calcium_UKBB	Longevity_90%	rs62211622	C	A	0.0017	0.0003	0.008	0.026
Calcium_UKBB	Longevity_90%	rs3091842	A	G	0.0088	0.0006	-0.033	0.045
Calcium_UKBB	Longevity_90%	rs6127099	A	T	0.0049	0.0003	0.007	0.023
Calcium_UKBB	Longevity_90%	rs2762938	A	G	0.0014	0.0002	-0.010	0.021
Calcium_UKBB	Longevity_90%	rs928760	C	T	0.0017	0.0003	-0.007	0.021
Calcium_UKBB	Longevity_90%	rs5760495	T	C	0.0015	0.0002	0.018	0.027
Calcium_UKBB	Longevity_90%	rs1544432	G	T	0.0016	0.0003	0.013	0.022
Calcium_UKBB	Longevity_90%	rs5751350	A	G	0.0015	0.0002	0.009	0.021
Calcium_UKBB	Longevity_90%_UKBB	rs1036332	C	A	0.0020	0.0003	0.021	0.030
Calcium_UKBB	Longevity_90%_UKBB	rs10754439	T	G	0.0010	0.0002	0.016	0.027
Calcium_UKBB	Longevity_90%_UKBB	rs10819178	G	T	0.0030	0.0002	-0.015	0.027
Calcium_UKBB	Longevity_90%_UKBB	rs10858935	A	G	-0.0020	0.0003	-0.021	0.028
Calcium_UKBB	Longevity_90%_UKBB	rs10917386	T	C	0.0020	0.0003	-0.024	0.028
Calcium_UKBB	Longevity_90%_UKBB	rs10958700	G	T	0.0020	0.0003	-0.015	0.031
Calcium_UKBB	Longevity_90%_UKBB	rs11078597	C	T	0.0050	0.0003	-0.036	0.034
Calcium_UKBB	Longevity_90%_UKBB	rs11117777	C	T	0.0020	0.0003	-0.005	0.036
Calcium_UKBB	Longevity_90%_UKBB	rs112174050	T	C	0.0100	0.0007	0.049	0.083
Calcium_UKBB	Longevity_90%_UKBB	rs112371897	T	C	0.0060	0.0004	-0.028	0.045
Calcium_UKBB	Longevity_90%_UKBB	rs115227958	T	C	-0.0070	0.0006	0.012	0.071
Calcium_UKBB	Longevity_90%_UKBB	rs11588907	T	C	-0.0010	0.0002	-0.009	0.028
Calcium_UKBB	Longevity_90%_UKBB	rs115946508	A	C	0.0030	0.0004	0.076	0.042
Calcium_UKBB	Longevity_90%_UKBB	rs11621792	T	C	0.0010	0.0002	-0.001	0.026
Calcium_UKBB	Longevity_90%_UKBB	rs11629876	T	C	-0.0020	0.0002	-0.012	0.028
Calcium_UKBB	Longevity_90%_UKBB	rs11671393	C	G	-0.0040	0.0006	0.000	0.068
Calcium_UKBB	Longevity_90%_UKBB	rs116769926	A	C	0.0070	0.0008	0.071	0.086
Calcium_UKBB	Longevity_90%_UKBB	rs117080167	T	C	-0.0030	0.0004	-0.061	0.048
Calcium_UKBB	Longevity_90%_UKBB	rs117080418	A	T	-0.0090	0.0012	-0.170	0.134
Calcium_UKBB	Longevity_90%_UKBB	rs117179023	T	C	-0.0060	0.0011	0.137	0.120
Calcium_UKBB	Longevity_90%_UKBB	rs117213754	A	G	0.0100	0.0010	0.072	0.109
Calcium_UKBB	Longevity_90%_UKBB	rs11730491	T	G	0.0020	0.0003	0.031	0.035
Calcium_UKBB	Longevity_90%_UKBB	rs117896857	T	C	-0.0050	0.0007	-0.014	0.081
Calcium_UKBB	Longevity_90%_UKBB	rs11792928	T	C	-0.0020	0.0003	0.010	0.029
Calcium_UKBB	Longevity_90%_UKBB	rs1187117	T	G	-0.0040	0.0003	0.126	0.034

Calcium_UKBB	Longevity_90%_UKBB	rs12132412	G	A	0.0030	0.0002	-0.031	0.027
Calcium_UKBB	Longevity_90%_UKBB	rs12135382	T	C	0.0020	0.0002	0.003	0.027
Calcium_UKBB	Longevity_90%_UKBB	rs12147703	A	G	-0.0030	0.0004	0.045	0.043
Calcium_UKBB	Longevity_90%_UKBB	rs12339541	A	C	-0.0060	0.0005	0.031	0.053
Calcium_UKBB	Longevity_90%_UKBB	rs12378991	A	G	-0.0030	0.0004	-0.031	0.048
Calcium_UKBB	Longevity_90%_UKBB	rs12519940	T	C	-0.0020	0.0003	0.007	0.029
Calcium_UKBB	Longevity_90%_UKBB	rs12583851	C	T	-0.0020	0.0003	0.032	0.030
Calcium_UKBB	Longevity_90%_UKBB	rs1260326	C	T	-0.0040	0.0002	-0.003	0.027
Calcium_UKBB	Longevity_90%_UKBB	rs12613807	C	T	0.0010	0.0002	0.015	0.026
Calcium_UKBB	Longevity_90%_UKBB	rs12675477	T	C	0.0020	0.0003	0.018	0.029
Calcium_UKBB	Longevity_90%_UKBB	rs12743084	G	C	-0.0020	0.0002	0.009	0.026
Calcium_UKBB	Longevity_90%_UKBB	rs12793731	T	C	0.0010	0.0002	-0.028	0.026
Calcium_UKBB	Longevity_90%_UKBB	rs12918968	C	A	-0.0030	0.0002	0.033	0.026
Calcium_UKBB	Longevity_90%_UKBB	rs12922549	T	C	-0.0020	0.0003	0.011	0.031
Calcium_UKBB	Longevity_90%_UKBB	rs12982234	T	C	-0.0050	0.0006	-0.209	0.067
Calcium_UKBB	Longevity_90%_UKBB	rs12998379	A	G	-0.0020	0.0003	0.038	0.033
Calcium_UKBB	Longevity_90%_UKBB	rs13107325	T	C	-0.0060	0.0004	-0.059	0.050
Calcium_UKBB	Longevity_90%_UKBB	rs13108218	G	A	-0.0040	0.0002	0.052	0.027
Calcium_UKBB	Longevity_90%_UKBB	rs13259549	C	T	0.0010	0.0002	0.011	0.027
Calcium_UKBB	Longevity_90%_UKBB	rs13389219	T	C	-0.0020	0.0002	0.058	0.027
Calcium_UKBB	Longevity_90%_UKBB	rs1354034	C	T	-0.0020	0.0002	-0.026	0.027
Calcium_UKBB	Longevity_90%_UKBB	rs1374161	A	G	-0.0020	0.0002	-0.026	0.026
Calcium_UKBB	Longevity_90%_UKBB	rs138789759	A	G	0.0040	0.0004	-0.018	0.050
Calcium_UKBB	Longevity_90%_UKBB	rs147233090	T	C	0.0090	0.0008	-0.029	0.085
Calcium_UKBB	Longevity_90%_UKBB	rs148148022	A	G	-0.0060	0.0010	0.077	0.116
Calcium_UKBB	Longevity_90%_UKBB	rs1497826	G	C	0.0020	0.0002	0.001	0.027
Calcium_UKBB	Longevity_90%_UKBB	rs149807892	T	C	0.0060	0.0009	0.209	0.104
Calcium_UKBB	Longevity_90%_UKBB	rs150268548	A	G	0.0030	0.0005	-0.078	0.052
Calcium_UKBB	Longevity_90%_UKBB	rs1544432	G	T	0.0020	0.0003	0.009	0.030
Calcium_UKBB	Longevity_90%_UKBB	rs164751	T	G	-0.0020	0.0002	-0.014	0.026
Calcium_UKBB	Longevity_90%_UKBB	rs165316	G	A	-0.0020	0.0003	-0.010	0.033
Calcium_UKBB	Longevity_90%_UKBB	rs1672991	G	A	0.0070	0.0005	0.128	0.052
Calcium_UKBB	Longevity_90%_UKBB	rs17132144	T	C	-0.0020	0.0004	-0.026	0.045
Calcium_UKBB	Longevity_90%_UKBB	rs17164683	T	C	-0.0020	0.0003	0.045	0.030

Calcium_UKBB	Longevity_90%_UKBB	rs17200894	G	C	0.0130	0.0003	-0.083	0.037
Calcium_UKBB	Longevity_90%_UKBB	rs17580	A	T	0.0040	0.0005	0.083	0.062
Calcium_UKBB	Longevity_90%_UKBB	rs1763519	C	G	-0.0030	0.0002	0.007	0.027
Calcium_UKBB	Longevity_90%_UKBB	rs17774672	A	G	-0.0030	0.0003	0.051	0.036
Calcium_UKBB	Longevity_90%_UKBB	rs17884869	A	G	-0.0100	0.0007	-0.159	0.082
Calcium_UKBB	Longevity_90%_UKBB	rs1801282	G	C	-0.0030	0.0004	-0.049	0.040
Calcium_UKBB	Longevity_90%_UKBB	rs1858800	T	C	0.0030	0.0002	-0.007	0.028
Calcium_UKBB	Longevity_90%_UKBB	rs2001884	A	T	-0.0020	0.0002	0.042	0.026
Calcium_UKBB	Longevity_90%_UKBB	rs2004315	T	C	0.0030	0.0002	-0.005	0.027
Calcium_UKBB	Longevity_90%_UKBB	rs2249825	C	G	-0.0010	0.0003	0.003	0.030
Calcium_UKBB	Longevity_90%_UKBB	rs2274224	C	G	-0.0020	0.0002	0.082	0.026
Calcium_UKBB	Longevity_90%_UKBB	rs2309233	C	T	0.0020	0.0003	-0.054	0.030
Calcium_UKBB	Longevity_90%_UKBB	rs2327774	C	T	-0.0020	0.0002	0.015	0.027
Calcium_UKBB	Longevity_90%_UKBB	rs2335534	A	G	-0.0040	0.0003	-0.004	0.034
Calcium_UKBB	Longevity_90%_UKBB	rs2343592	G	A	-0.0020	0.0003	-0.009	0.029
Calcium_UKBB	Longevity_90%_UKBB	rs2370218	T	G	-0.0020	0.0003	0.004	0.031
Calcium_UKBB	Longevity_90%_UKBB	rs2395158	G	A	-0.0020	0.0003	0.045	0.035
Calcium_UKBB	Longevity_90%_UKBB	rs2419886	T	C	-0.0020	0.0003	-0.094	0.030
Calcium_UKBB	Longevity_90%_UKBB	rs257380	A	T	-0.0020	0.0002	0.018	0.026
Calcium_UKBB	Longevity_90%_UKBB	rs2647242	G	A	-0.0020	0.0003	0.041	0.033
Calcium_UKBB	Longevity_90%_UKBB	rs2762938	A	G	0.0010	0.0002	0.003	0.027
Calcium_UKBB	Longevity_90%_UKBB	rs28520334	T	G	0.0020	0.0004	-0.006	0.040
Calcium_UKBB	Longevity_90%_UKBB	rs28929474	T	C	0.0110	0.0008	-0.030	0.093
Calcium_UKBB	Longevity_90%_UKBB	rs2930191	A	G	-0.0020	0.0002	0.028	0.027
Calcium_UKBB	Longevity_90%_UKBB	rs3011642	T	C	0.0020	0.0003	-0.005	0.030
Calcium_UKBB	Longevity_90%_UKBB	rs3026445	C	T	-0.0020	0.0002	0.012	0.027
Calcium_UKBB	Longevity_90%_UKBB	rs302650	A	G	-0.0020	0.0002	0.015	0.026
Calcium_UKBB	Longevity_90%_UKBB	rs3091842	A	G	0.0090	0.0006	-0.022	0.066
Calcium_UKBB	Longevity_90%_UKBB	rs3133548	T	C	0.0020	0.0003	0.036	0.038
Calcium_UKBB	Longevity_90%_UKBB	rs34290411	G	C	0.0020	0.0003	-0.005	0.029
Calcium_UKBB	Longevity_90%_UKBB	rs35118755	T	C	0.0020	0.0003	0.020	0.037
Calcium_UKBB	Longevity_90%_UKBB	rs35320690	C	T	0.0020	0.0003	0.052	0.029
Calcium_UKBB	Longevity_90%_UKBB	rs35590487	T	C	-0.0020	0.0003	-0.021	0.031
Calcium_UKBB	Longevity_90%_UKBB	rs35751693	T	C	0.0040	0.0006	-0.074	0.069

Calcium_UKBB	Longevity_90%_UKBB	rs35852840	A	C	0.0030	0.0005	0.061	0.056
Calcium_UKBB	Longevity_90%_UKBB	rs36032443	C	A	-0.0020	0.0002	-0.014	0.027
Calcium_UKBB	Longevity_90%_UKBB	rs36086195	T	C	0.0020	0.0002	0.012	0.027
Calcium_UKBB	Longevity_90%_UKBB	rs36104352	C	A	0.0030	0.0004	-0.010	0.040
Calcium_UKBB	Longevity_90%_UKBB	rs3748861	A	C	-0.0020	0.0003	-0.038	0.033
Calcium_UKBB	Longevity_90%_UKBB	rs3794695	T	C	0.0020	0.0003	-0.055	0.033
Calcium_UKBB	Longevity_90%_UKBB	rs3795243	C	G	0.0020	0.0004	-0.020	0.039
Calcium_UKBB	Longevity_90%_UKBB	rs3822858	C	T	-0.0020	0.0002	-0.005	0.027
Calcium_UKBB	Longevity_90%_UKBB	rs3931841	G	A	-0.0020	0.0003	0.040	0.028
Calcium_UKBB	Longevity_90%_UKBB	rs4082330	T	C	0.0020	0.0003	-0.017	0.034
Calcium_UKBB	Longevity_90%_UKBB	rs41278174	A	G	0.0050	0.0007	-0.023	0.080
Calcium_UKBB	Longevity_90%_UKBB	rs4239142	G	A	-0.0020	0.0003	-0.026	0.030
Calcium_UKBB	Longevity_90%_UKBB	rs424062	G	A	-0.0020	0.0004	0.056	0.037
Calcium_UKBB	Longevity_90%_UKBB	rs4324076	C	A	-0.0020	0.0002	0.000	0.026
Calcium_UKBB	Longevity_90%_UKBB	rs4633480	A	G	0.0020	0.0002	-0.011	0.026
Calcium_UKBB	Longevity_90%_UKBB	rs4653767	C	T	-0.0020	0.0003	0.003	0.029
Calcium_UKBB	Longevity_90%_UKBB	rs4721467	T	A	-0.0020	0.0003	-0.043	0.030
Calcium_UKBB	Longevity_90%_UKBB	rs4758621	G	A	-0.0020	0.0003	0.054	0.028
Calcium_UKBB	Longevity_90%_UKBB	rs4790310	T	C	-0.0020	0.0002	0.026	0.027
Calcium_UKBB	Longevity_90%_UKBB	rs4841132	G	A	0.0050	0.0004	-0.058	0.045
Calcium_UKBB	Longevity_90%_UKBB	rs4935009	C	A	-0.0020	0.0003	0.016	0.039
Calcium_UKBB	Longevity_90%_UKBB	rs4938642	C	G	0.0030	0.0004	0.050	0.050
Calcium_UKBB	Longevity_90%_UKBB	rs498490	T	C	-0.0030	0.0003	-0.011	0.035
Calcium_UKBB	Longevity_90%_UKBB	rs55772024	A	G	-0.0020	0.0003	0.035	0.030
Calcium_UKBB	Longevity_90%_UKBB	rs56406311	T	C	0.0020	0.0002	0.018	0.027
Calcium_UKBB	Longevity_90%_UKBB	rs5751350	A	G	0.0020	0.0002	-0.003	0.028
Calcium_UKBB	Longevity_90%_UKBB	rs5760495	T	C	0.0010	0.0002	-0.018	0.028
Calcium_UKBB	Longevity_90%_UKBB	rs59821684	G	A	0.0050	0.0007	0.000	0.078
Calcium_UKBB	Longevity_90%_UKBB	rs60616569	A	G	-0.0020	0.0003	-0.039	0.031
Calcium_UKBB	Longevity_90%_UKBB	rs6127099	T	A	-0.0050	0.0003	0.044	0.030
Calcium_UKBB	Longevity_90%_UKBB	rs62071306	C	A	-0.0020	0.0003	-0.047	0.030
Calcium_UKBB	Longevity_90%_UKBB	rs62134679	C	T	0.0020	0.0003	0.011	0.037
Calcium_UKBB	Longevity_90%_UKBB	rs62211622	A	C	-0.0020	0.0003	-0.010	0.034
Calcium_UKBB	Longevity_90%_UKBB	rs62268807	A	T	-0.0020	0.0003	0.014	0.031

Calcium_UKBB	Longevity_90%_UKBB	rs62309863	T	G	-0.0010	0.0002	0.039	0.027
Calcium_UKBB	Longevity_90%_UKBB	rs62472728	T	C	0.0030	0.0005	-0.057	0.055
Calcium_UKBB	Longevity_90%_UKBB	rs634916	T	A	-0.0010	0.0002	0.069	0.026
Calcium_UKBB	Longevity_90%_UKBB	rs6580981	A	G	-0.0020	0.0002	0.021	0.026
Calcium_UKBB	Longevity_90%_UKBB	rs66920316	A	G	-0.0020	0.0003	-0.010	0.033
Calcium_UKBB	Longevity_90%_UKBB	rs6698689	A	G	-0.0030	0.0004	0.015	0.040
Calcium_UKBB	Longevity_90%_UKBB	rs6741561	T	C	-0.0030	0.0002	0.014	0.027
Calcium_UKBB	Longevity_90%_UKBB	rs6841429	A	C	-0.0040	0.0003	-0.023	0.035
Calcium_UKBB	Longevity_90%_UKBB	rs7003580	T	C	0.0010	0.0002	0.038	0.027
Calcium_UKBB	Longevity_90%_UKBB	rs710217	G	A	0.0020	0.0002	-0.041	0.026
Calcium_UKBB	Longevity_90%_UKBB	rs71565393	T	C	0.0020	0.0003	-0.036	0.034
Calcium_UKBB	Longevity_90%_UKBB	rs71658797	A	T	0.0020	0.0004	-0.021	0.040
Calcium_UKBB	Longevity_90%_UKBB	rs7221118	C	T	-0.0020	0.0003	-0.046	0.032
Calcium_UKBB	Longevity_90%_UKBB	rs72697816	T	C	0.0020	0.0003	-0.007	0.035
Calcium_UKBB	Longevity_90%_UKBB	rs73632745	T	C	-0.0050	0.0004	0.042	0.050
Calcium_UKBB	Longevity_90%_UKBB	rs7370877	G	A	-0.0020	0.0002	-0.047	0.027
Calcium_UKBB	Longevity_90%_UKBB	rs7402977	A	G	-0.0010	0.0003	-0.013	0.030
Calcium_UKBB	Longevity_90%_UKBB	rs74230087	A	G	-0.0050	0.0004	-0.026	0.049
Calcium_UKBB	Longevity_90%_UKBB	rs75448233	T	C	0.0030	0.0005	-0.079	0.059
Calcium_UKBB	Longevity_90%_UKBB	rs7546838	G	A	0.0020	0.0002	-0.067	0.028
Calcium_UKBB	Longevity_90%_UKBB	rs7559013	C	A	0.0020	0.0003	-0.069	0.039
Calcium_UKBB	Longevity_90%_UKBB	rs75702986	A	G	0.0030	0.0003	-0.029	0.034
Calcium_UKBB	Longevity_90%_UKBB	rs7587636	A	G	-0.0020	0.0002	0.007	0.026
Calcium_UKBB	Longevity_90%_UKBB	rs75895430	G	C	0.0070	0.0007	-0.099	0.075
Calcium_UKBB	Longevity_90%_UKBB	rs7592216	T	G	-0.0020	0.0003	0.042	0.039
Calcium_UKBB	Longevity_90%_UKBB	rs7599	G	A	-0.0020	0.0002	-0.046	0.027
Calcium_UKBB	Longevity_90%_UKBB	rs76947531	C	T	-0.0080	0.0004	0.055	0.042
Calcium_UKBB	Longevity_90%_UKBB	rs7730344	A	C	0.0020	0.0003	-0.011	0.029
Calcium_UKBB	Longevity_90%_UKBB	rs775249	T	C	-0.0010	0.0003	0.015	0.029
Calcium_UKBB	Longevity_90%_UKBB	rs77542162	G	A	-0.0080	0.0008	-0.150	0.086
Calcium_UKBB	Longevity_90%_UKBB	rs77704893	C	T	0.0040	0.0005	0.054	0.050
Calcium_UKBB	Longevity_90%_UKBB	rs777588	A	G	-0.0030	0.0002	0.065	0.026
Calcium_UKBB	Longevity_90%_UKBB	rs7786368	C	T	-0.0020	0.0002	0.040	0.026
Calcium_UKBB	Longevity_90%_UKBB	rs79501693	C	T	-0.0050	0.0008	0.050	0.092

Calcium_UKBB	Longevity_90%_UKBB	rs80350997	A	G	0.0040	0.0004	0.018	0.049
Calcium_UKBB	Longevity_90%_UKBB	rs8054061	C	T	0.0020	0.0002	-0.042	0.026
Calcium_UKBB	Longevity_90%_UKBB	rs883951	G	A	0.0020	0.0003	0.028	0.030
Calcium_UKBB	Longevity_90%_UKBB	rs928760	T	C	-0.0020	0.0003	-0.031	0.028
Calcium_UKBB	Longevity_90%_UKBB	rs9379881	C	T	-0.0010	0.0002	0.029	0.026
Calcium_UKBB	Longevity_90%_UKBB	rs9388399	C	T	-0.0020	0.0003	0.035	0.028
Calcium_UKBB	Longevity_90%_UKBB	rs9399697	T	C	0.0010	0.0002	0.001	0.026
Calcium_UKBB	Longevity_90%_UKBB	rs9419741	G	A	0.0010	0.0002	-0.033	0.026
Calcium_UKBB	Longevity_90%_UKBB	rs9447004	G	A	-0.0040	0.0002	0.031	0.026
Calcium_UKBB	Longevity_90%_UKBB	rs945890	T	A	-0.0020	0.0003	-0.006	0.029
Calcium_UKBB	Longevity_90%_UKBB	rs948737	T	C	-0.0010	0.0002	0.041	0.028
Calcium_UKBB	Longevity_90%_UKBB	rs949300	A	T	0.0010	0.0002	0.018	0.027
Calcium_UKBB	Longevity_90%_UKBB	rs9530	G	A	-0.0030	0.0002	0.019	0.026
Calcium_UKBB	Longevity_90%_UKBB	rs9532958	A	G	0.0030	0.0003	0.050	0.037

EA, effect allele; NEA, non-effect allele; SNP, single nucleotide polymorphism.

Supplementary Table 7. Data for analyses of serum 25(OH)D

Exposure	Outcome	SNP	EA	NEA	Exposure_beta	Exposure_se	Outcome_beta	Outcome_se
25(OH)D_Jiang_X_Manousaki_D	FinnGen_CA	rs10741657	A	G	0.090	0.007	0.004	0.009
25(OH)D_Jiang_X_Manousaki_D	FinnGen_CA	rs10745742	T	C	0.050	0.007	-0.001	0.009
25(OH)D_Jiang_X_Manousaki_D	FinnGen_CA	rs117913124	G	A	0.430	0.020	0.039	0.032
25(OH)D_Jiang_X_Manousaki_D	FinnGen_CA	rs12785878	T	G	0.110	0.007	0.006	0.009
25(OH)D_Jiang_X_Manousaki_D	FinnGen_CA	rs17216707	T	C	0.080	0.008	0.003	0.011
25(OH)D_Jiang_X_Manousaki_D	FinnGen_CA	rs3755967	C	T	0.270	0.007	-0.009	0.011
25(OH)D_Jiang_X_Manousaki_D	FinnGen_CA	rs8018720	G	C	0.050	0.009	-0.001	0.012
25(OH)D_Jiang_X_Manousaki_D	FinnGen_CVD	rs10741657	A	G	0.0900	0.0070	0.008	0.008
25(OH)D_Jiang_X_Manousaki_D	FinnGen_CVD	rs10745742	T	C	0.0500	0.0070	0.005	0.008
25(OH)D_Jiang_X_Manousaki_D	FinnGen_CVD	rs117913124	G	A	0.4300	0.0200	0.005	0.028
25(OH)D_Jiang_X_Manousaki_D	FinnGen_CVD	rs12785878	T	G	0.1100	0.0070	-0.007	0.008
25(OH)D_Jiang_X_Manousaki_D	FinnGen_CVD	rs17216707	T	C	0.0800	0.0080	0.011	0.010
25(OH)D_Jiang_X_Manousaki_D	FinnGen_CVD	rs3755967	C	T	0.2700	0.0070	0.009	0.010
25(OH)D_Jiang_X_Manousaki_D	FinnGen_CVD	rs8018720	G	C	0.0500	0.0090	0.000	0.011
25(OH)D_Jiang_X_Manousaki_D	Longevity_90%	rs3755967	C	T	0.270	0.007	-0.011	0.021
25(OH)D_Jiang_X_Manousaki_D	Longevity_90%	rs117913124	G	A	0.430	0.020	-0.042	0.078
25(OH)D_Jiang_X_Manousaki_D	Longevity_90%	rs10741657	A	G	0.090	0.007	-0.009	0.020
25(OH)D_Jiang_X_Manousaki_D	Longevity_90%	rs12785878	T	G	0.110	0.007	-0.033	0.021
25(OH)D_Jiang_X_Manousaki_D	Longevity_90%	rs10745742	T	C	0.050	0.007	0.015	0.020
25(OH)D_Jiang_X_Manousaki_D	Longevity_90%	rs8018720	G	C	0.050	0.009	-0.032	0.025
25(OH)D_Jiang_X_Manousaki_D	Longevity_90%	rs17216707	T	C	0.080	0.008	0.012	0.026
25(OH)D_Jiang_X_Manousaki_D	Longevity_90%_UKBB	rs10741657	A	G	0.090	0.007	0.008	0.027
25(OH)D_Jiang_X_Manousaki_D	Longevity_90%_UKBB	rs10745742	T	C	0.050	0.007	0.095	0.027
25(OH)D_Jiang_X_Manousaki_D	Longevity_90%_UKBB	rs117913124	G	A	0.430	0.020	-0.117	0.080
25(OH)D_Jiang_X_Manousaki_D	Longevity_90%_UKBB	rs12785878	T	G	0.110	0.007	-0.024	0.032
25(OH)D_Jiang_X_Manousaki_D	Longevity_90%_UKBB	rs17216707	T	C	0.080	0.008	-0.041	0.034
25(OH)D_Jiang_X_Manousaki_D	Longevity_90%_UKBB	rs3755967	C	T	0.270	0.007	-0.055	0.029
25(OH)D_Jiang_X_Manousaki_D	Longevity_90%_UKBB	rs8018720	G	C	0.050	0.009	-0.042	0.034

25(OH)D_UKBB	FinnGen_CA	rs10070734	T	C	-0.013	0.002	0.004	0.010
25(OH)D_UKBB	FinnGen_CA	rs10085881	T	C	0.015	0.002	0.001	0.010
25(OH)D_UKBB	FinnGen_CA	rs1038165	C	T	-0.012	0.002	0.008	0.009
25(OH)D_UKBB	FinnGen_CA	rs10426	G	A	-0.025	0.002	-0.001	0.010
25(OH)D_UKBB	FinnGen_CA	rs10454087	C	T	0.014	0.002	-0.005	0.010
25(OH)D_UKBB	FinnGen_CA	rs1047891	C	A	0.015	0.002	-0.005	0.010
25(OH)D_UKBB	FinnGen_CA	rs10859995	T	C	0.044	0.002	-0.006	0.009
25(OH)D_UKBB	FinnGen_CA	rs10887718	C	T	0.011	0.002	0.007	0.009
25(OH)D_UKBB	FinnGen_CA	rs10908419	G	A	0.012	0.002	0.013	0.009
25(OH)D_UKBB	FinnGen_CA	rs10908465	C	T	-0.020	0.002	0.005	0.010
25(OH)D_UKBB	FinnGen_CA	rs11076175	A	G	-0.018	0.003	0.009	0.012
25(OH)D_UKBB	FinnGen_CA	rs11108368	A	G	-0.014	0.002	-0.002	0.009
25(OH)D_UKBB	FinnGen_CA	rs11127186	T	C	-0.011	0.002	-0.012	0.009
25(OH)D_UKBB	FinnGen_CA	rs11182428	T	C	0.013	0.002	-0.007	0.009
25(OH)D_UKBB	FinnGen_CA	rs11264322	G	A	0.013	0.002	0.007	0.009
25(OH)D_UKBB	FinnGen_CA	rs11458206	T	TG	-0.014	0.002	0.003	0.010
25(OH)D_UKBB	FinnGen_CA	rs1149605	T	C	-0.023	0.003	0.017	0.012
25(OH)D_UKBB	FinnGen_CA	rs11542462	G	A	0.024	0.003	0.018	0.015
25(OH)D_UKBB	FinnGen_CA	rs115621755	C	T	0.012	0.002	0.007	0.009
25(OH)D_UKBB	FinnGen_CA	rs11591147	G	T	-0.045	0.007	-0.001	0.024
25(OH)D_UKBB	FinnGen_CA	rs11606	C	G	-0.012	0.002	-0.007	0.009
25(OH)D_UKBB	FinnGen_CA	rs116970203	G	A	0.428	0.006	0.043	0.032
25(OH)D_UKBB	FinnGen_CA	rs11732896	G	A	0.016	0.002	0.004	0.010
25(OH)D_UKBB	FinnGen_CA	rs117576073	G	T	0.215	0.009	0.011	0.028
25(OH)D_UKBB	FinnGen_CA	rs12056768	T	G	0.025	0.002	-0.009	0.009
25(OH)D_UKBB	FinnGen_CA	rs12123821	C	T	-0.074	0.005	-0.055	0.023
25(OH)D_UKBB	FinnGen_CA	rs1229984	T	C	0.047	0.006	-0.102	0.060
25(OH)D_UKBB	FinnGen_CA	rs12317268	A	G	0.021	0.003	-0.021	0.010
25(OH)D_UKBB	FinnGen_CA	rs12372115	G	T	0.022	0.004	-0.006	0.020
25(OH)D_UKBB	FinnGen_CA	rs1260326	T	C	-0.021	0.002	0.003	0.009
25(OH)D_UKBB	FinnGen_CA	rs12794714	G	A	0.113	0.002	-0.013	0.009

25(OH)D_UKBB	FinnGen_CA	rs12798050	C	T	-0.048	0.004	-0.010	0.010
25(OH)D_UKBB	FinnGen_CA	rs12803256	A	G	-0.067	0.004	-0.007	0.009
25(OH)D_UKBB	FinnGen_CA	rs12881545	G	C	-0.012	0.002	0.017	0.009
25(OH)D_UKBB	FinnGen_CA	rs13060130	C	T	0.017	0.003	0.005	0.012
25(OH)D_UKBB	FinnGen_CA	rs13104260	G	A	-0.020	0.002	0.009	0.011
25(OH)D_UKBB	FinnGen_CA	rs13284054	T	C	-0.018	0.003	-0.018	0.014
25(OH)D_UKBB	FinnGen_CA	rs1352846	A	G	0.196	0.002	-0.016	0.011
25(OH)D_UKBB	FinnGen_CA	rs140371183	A	G	-0.063	0.007	0.000	0.050
25(OH)D_UKBB	FinnGen_CA	rs142158911	G	A	-0.025	0.003	-0.017	0.015
25(OH)D_UKBB	FinnGen_CA	rs1660839	G	A	-0.015	0.002	0.003	0.011
25(OH)D_UKBB	FinnGen_CA	rs17216707	T	C	0.032	0.003	0.003	0.011
25(OH)D_UKBB	FinnGen_CA	rs17231506	C	T	0.014	0.002	-0.001	0.010
25(OH)D_UKBB	FinnGen_CA	rs1800588	C	T	0.034	0.002	0.017	0.010
25(OH)D_UKBB	FinnGen_CA	rs187429064	A	G	-0.068	0.009	-0.042	0.021
25(OH)D_UKBB	FinnGen_CA	rs1933064	G	A	-0.015	0.002	-0.017	0.009
25(OH)D_UKBB	FinnGen_CA	rs2012736	C	A	0.048	0.004	-0.031	0.017
25(OH)D_UKBB	FinnGen_CA	rs2037511	G	A	-0.018	0.003	-0.006	0.012
25(OH)D_UKBB	FinnGen_CA	rs2074735	G	C	-0.028	0.004	0.003	0.013
25(OH)D_UKBB	FinnGen_CA	rs212100	T	C	0.066	0.003	-0.001	0.012
25(OH)D_UKBB	FinnGen_CA	rs2131925	G	T	0.023	0.002	0.001	0.010
25(OH)D_UKBB	FinnGen_CA	rs2207132	G	A	0.035	0.006	-0.009	0.018
25(OH)D_UKBB	FinnGen_CA	rs2229742	G	C	0.025	0.003	-0.019	0.014
25(OH)D_UKBB	FinnGen_CA	rs2248551	G	A	0.023	0.003	-0.009	0.011
25(OH)D_UKBB	FinnGen_CA	rs2346264	A	C	0.014	0.002	-0.020	0.012
25(OH)D_UKBB	FinnGen_CA	rs2585442	C	G	-0.027	0.002	0.000	0.010
25(OH)D_UKBB	FinnGen_CA	rs261291	T	C	0.028	0.002	-0.021	0.009
25(OH)D_UKBB	FinnGen_CA	rs2710651	G	A	0.011	0.002	-0.012	0.009
25(OH)D_UKBB	FinnGen_CA	rs2725371	A	G	-0.012	0.002	-0.002	0.010
25(OH)D_UKBB	FinnGen_CA	rs2762943	T	G	-0.045	0.004	-0.010	0.015
25(OH)D_UKBB	FinnGen_CA	rs28364331	A	G	-0.070	0.007	0.014	0.081
25(OH)D_UKBB	FinnGen_CA	rs28367476	A	G	0.014	0.002	-0.024	0.009

25(OH)D_UKBB	FinnGen_CA	rs28374650	C	T	0.013	0.002	0.001	0.011
25(OH)D_UKBB	FinnGen_CA	rs2847500	G	A	0.022	0.003	0.006	0.013
25(OH)D_UKBB	FinnGen_CA	rs28692966	G	A	-0.015	0.002	-0.012	0.010
25(OH)D_UKBB	FinnGen_CA	rs2952289	C	T	-0.018	0.002	-0.016	0.012
25(OH)D_UKBB	FinnGen_CA	rs31612	T	C	0.015	0.003	0.009	0.011
25(OH)D_UKBB	FinnGen_CA	rs325384	C	T	0.014	0.002	-0.003	0.010
25(OH)D_UKBB	FinnGen_CA	rs34290760	C	G	0.039	0.006	0.007	0.019
25(OH)D_UKBB	FinnGen_CA	rs35057908	A	T	-0.016	0.002	-0.002	0.010
25(OH)D_UKBB	FinnGen_CA	rs35408430	C	T	0.021	0.002	0.005	0.009
25(OH)D_UKBB	FinnGen_CA	rs3814995	C	T	0.012	0.002	0.007	0.009
25(OH)D_UKBB	FinnGen_CA	rs3849374	G	C	0.016	0.003	-0.022	0.011
25(OH)D_UKBB	FinnGen_CA	rs4121823	T	A	0.019	0.003	0.022	0.012
25(OH)D_UKBB	FinnGen_CA	rs4327060	C	T	0.025	0.004	-0.001	0.014
25(OH)D_UKBB	FinnGen_CA	rs4364259	G	A	-0.016	0.003	0.004	0.011
25(OH)D_UKBB	FinnGen_CA	rs4418728	G	T	-0.011	0.002	0.000	0.009
25(OH)D_UKBB	FinnGen_CA	rs4575545	G	A	0.016	0.002	0.000	0.010
25(OH)D_UKBB	FinnGen_CA	rs4616820	C	T	0.012	0.002	-0.013	0.009
25(OH)D_UKBB	FinnGen_CA	rs484195	A	G	0.016	0.002	-0.007	0.010
25(OH)D_UKBB	FinnGen_CA	rs541041	G	A	0.016	0.003	0.010	0.011
25(OH)D_UKBB	FinnGen_CA	rs55829990	T	C	0.019	0.002	0.000	0.009
25(OH)D_UKBB	FinnGen_CA	rs590215	C	T	0.013	0.002	-0.006	0.011
25(OH)D_UKBB	FinnGen_CA	rs6003456	T	A	0.013	0.002	-0.010	0.012
25(OH)D_UKBB	FinnGen_CA	rs6123359	A	G	-0.025	0.003	0.027	0.014
25(OH)D_UKBB	FinnGen_CA	rs613808	A	G	-0.016	0.002	-0.008	0.009
25(OH)D_UKBB	FinnGen_CA	rs61816761	G	A	-0.077	0.010	-0.043	0.085
25(OH)D_UKBB	FinnGen_CA	rs61883501	A	C	-0.033	0.006	0.010	0.034
25(OH)D_UKBB	FinnGen_CA	rs61891388	T	G	-0.013	0.002	0.017	0.009
25(OH)D_UKBB	FinnGen_CA	rs62007299	G	A	0.013	0.002	0.005	0.010
25(OH)D_UKBB	FinnGen_CA	rs6671730	G	A	0.015	0.002	0.015	0.009
25(OH)D_UKBB	FinnGen_CA	rs6672758	C	T	-0.017	0.003	0.007	0.010
25(OH)D_UKBB	FinnGen_CA	rs6782190	G	A	0.018	0.002	-0.012	0.010

25(OH)D_UKBB	FinnGen_CA	rs705117	C	T	-0.032	0.003	-0.016	0.013
25(OH)D_UKBB	FinnGen_CA	rs7149014	T	C	0.013	0.002	0.000	0.009
25(OH)D_UKBB	FinnGen_CA	rs727857	G	A	0.014	0.002	0.000	0.009
25(OH)D_UKBB	FinnGen_CA	rs72834856	T	G	0.025	0.004	-0.024	0.018
25(OH)D_UKBB	FinnGen_CA	rs72997623	C	A	-0.028	0.004	0.002	0.016
25(OH)D_UKBB	FinnGen_CA	rs73413596	T	C	-0.022	0.004	0.001	0.019
25(OH)D_UKBB	FinnGen_CA	rs7412	C	T	-0.030	0.004	-0.017	0.020
25(OH)D_UKBB	FinnGen_CA	rs7522116	C	T	0.013	0.002	-0.010	0.009
25(OH)D_UKBB	FinnGen_CA	rs7528419	A	G	-0.020	0.002	0.000	0.011
25(OH)D_UKBB	FinnGen_CA	rs7569755	G	A	-0.014	0.002	-0.012	0.011
25(OH)D_UKBB	FinnGen_CA	rs75741381	C	G	0.016	0.003	-0.006	0.012
25(OH)D_UKBB	FinnGen_CA	rs7604788	C	T	-0.036	0.006	0.028	0.019
25(OH)D_UKBB	FinnGen_CA	rs76798800	G	T	0.017	0.002	-0.012	0.011
25(OH)D_UKBB	FinnGen_CA	rs77532868	C	T	-0.027	0.004	0.017	0.025
25(OH)D_UKBB	FinnGen_CA	rs77924615	G	A	0.017	0.003	0.008	0.011
25(OH)D_UKBB	FinnGen_CA	rs78151190	A	C	0.017	0.003	-0.013	0.015
25(OH)D_UKBB	FinnGen_CA	rs78168201	C	T	-0.066	0.009	-0.031	0.066
25(OH)D_UKBB	FinnGen_CA	rs78649910	T	A	0.021	0.003	0.000	0.014
25(OH)D_UKBB	FinnGen_CA	rs8018720	G	C	0.038	0.003	-0.001	0.012
25(OH)D_UKBB	FinnGen_CA	rs804281	A	G	-0.015	0.002	0.003	0.013
25(OH)D_UKBB	FinnGen_CA	rs8091117	C	A	0.026	0.004	0.009	0.017
25(OH)D_UKBB	FinnGen_CA	rs8113404	C	T	-0.013	0.002	-0.001	0.010
25(OH)D_UKBB	FinnGen_CA	rs867772	A	G	0.014	0.002	0.008	0.010
25(OH)D_UKBB	FinnGen_CA	rs9476310	C	T	-0.012	0.002	0.013	0.009
25(OH)D_UKBB	FinnGen_CA	rs9490317	T	C	-0.011	0.002	-0.001	0.009
25(OH)D_UKBB	FinnGen_CA	rs964184	G	C	-0.035	0.003	-0.005	0.013
25(OH)D_UKBB	FinnGen_CA	rs9861009	T	C	-0.014	0.002	-0.005	0.010
25(OH)D_UKBB	FinnGen_CVD	rs10070734	T	C	-0.013	0.002	0.001	0.009
25(OH)D_UKBB	FinnGen_CVD	rs10085881	T	C	0.015	0.002	0.007	0.009
25(OH)D_UKBB	FinnGen_CVD	rs1038165	C	T	-0.012	0.002	0.001	0.008
25(OH)D_UKBB	FinnGen_CVD	rs10426	G	A	-0.025	0.002	0.003	0.009

25(OH)D_UKBB	FinnGen_CVD	rs10454087	C	T	0.014	0.002	-0.003	0.009
25(OH)D_UKBB	FinnGen_CVD	rs1047891	C	A	0.015	0.002	0.010	0.008
25(OH)D_UKBB	FinnGen_CVD	rs10859995	T	C	0.044	0.002	0.013	0.008
25(OH)D_UKBB	FinnGen_CVD	rs10887718	C	T	0.011	0.002	0.001	0.008
25(OH)D_UKBB	FinnGen_CVD	rs10908419	G	A	0.012	0.002	0.011	0.008
25(OH)D_UKBB	FinnGen_CVD	rs10908465	C	T	-0.020	0.002	0.004	0.009
25(OH)D_UKBB	FinnGen_CVD	rs11076175	A	G	-0.018	0.003	-0.009	0.010
25(OH)D_UKBB	FinnGen_CVD	rs11108368	A	G	-0.014	0.002	0.007	0.008
25(OH)D_UKBB	FinnGen_CVD	rs11127186	T	C	-0.011	0.002	-0.010	0.008
25(OH)D_UKBB	FinnGen_CVD	rs11182428	T	C	0.013	0.002	-0.004	0.008
25(OH)D_UKBB	FinnGen_CVD	rs11264322	G	A	0.013	0.002	-0.001	0.008
25(OH)D_UKBB	FinnGen_CVD	rs11458206	T	TG	-0.014	0.002	0.005	0.008
25(OH)D_UKBB	FinnGen_CVD	rs1149605	T	C	-0.023	0.003	-0.001	0.010
25(OH)D_UKBB	FinnGen_CVD	rs11542462	G	A	0.024	0.003	0.005	0.013
25(OH)D_UKBB	FinnGen_CVD	rs115621755	C	T	0.012	0.002	-0.020	0.008
25(OH)D_UKBB	FinnGen_CVD	rs11591147	G	T	-0.045	0.007	0.037	0.021
25(OH)D_UKBB	FinnGen_CVD	rs11606	C	G	-0.012	0.002	-0.003	0.008
25(OH)D_UKBB	FinnGen_CVD	rs116970203	G	A	0.428	0.006	0.005	0.028
25(OH)D_UKBB	FinnGen_CVD	rs11732896	G	A	0.016	0.002	-0.005	0.009
25(OH)D_UKBB	FinnGen_CVD	rs117576073	G	T	0.215	0.009	-0.018	0.024
25(OH)D_UKBB	FinnGen_CVD	rs12056768	T	G	0.025	0.002	-0.007	0.008
25(OH)D_UKBB	FinnGen_CVD	rs12123821	C	T	-0.074	0.005	-0.005	0.020
25(OH)D_UKBB	FinnGen_CVD	rs1229984	T	C	0.047	0.006	-0.016	0.054
25(OH)D_UKBB	FinnGen_CVD	rs12317268	A	G	0.021	0.003	0.017	0.009
25(OH)D_UKBB	FinnGen_CVD	rs12372115	G	T	0.022	0.004	-0.013	0.017
25(OH)D_UKBB	FinnGen_CVD	rs1260326	T	C	-0.021	0.002	0.006	0.008
25(OH)D_UKBB	FinnGen_CVD	rs12794714	G	A	0.113	0.002	0.004	0.008
25(OH)D_UKBB	FinnGen_CVD	rs12798050	C	T	-0.048	0.004	0.009	0.008
25(OH)D_UKBB	FinnGen_CVD	rs12803256	A	G	-0.067	0.004	0.005	0.008
25(OH)D_UKBB	FinnGen_CVD	rs12881545	G	C	-0.012	0.002	0.004	0.008
25(OH)D_UKBB	FinnGen_CVD	rs13060130	C	T	0.017	0.003	0.018	0.010

25(OH)D_UKBB	FinnGen_CVD	rs13104260	G	A	-0.020	0.002	0.006	0.009
25(OH)D_UKBB	FinnGen_CVD	rs13284054	T	C	-0.018	0.003	0.009	0.013
25(OH)D_UKBB	FinnGen_CVD	rs1352846	A	G	0.196	0.002	0.007	0.010
25(OH)D_UKBB	FinnGen_CVD	rs140371183	A	G	-0.063	0.007	-0.065	0.044
25(OH)D_UKBB	FinnGen_CVD	rs142158911	G	A	-0.025	0.003	0.020	0.013
25(OH)D_UKBB	FinnGen_CVD	rs1660839	G	A	-0.015	0.002	0.006	0.010
25(OH)D_UKBB	FinnGen_CVD	rs17216707	T	C	0.032	0.003	0.011	0.010
25(OH)D_UKBB	FinnGen_CVD	rs17231506	C	T	0.014	0.002	0.017	0.009
25(OH)D_UKBB	FinnGen_CVD	rs1800588	C	T	0.034	0.002	-0.026	0.009
25(OH)D_UKBB	FinnGen_CVD	rs187429064	A	G	-0.068	0.009	-0.025	0.018
25(OH)D_UKBB	FinnGen_CVD	rs1933064	G	A	-0.015	0.002	0.005	0.008
25(OH)D_UKBB	FinnGen_CVD	rs2012736	C	A	0.048	0.004	-0.013	0.015
25(OH)D_UKBB	FinnGen_CVD	rs2037511	G	A	-0.018	0.003	0.000	0.011
25(OH)D_UKBB	FinnGen_CVD	rs2074735	G	C	-0.028	0.004	0.008	0.011
25(OH)D_UKBB	FinnGen_CVD	rs212100	T	C	0.066	0.003	-0.009	0.011
25(OH)D_UKBB	FinnGen_CVD	rs2131925	G	T	0.023	0.002	-0.002	0.009
25(OH)D_UKBB	FinnGen_CVD	rs2207132	G	A	0.035	0.006	-0.003	0.016
25(OH)D_UKBB	FinnGen_CVD	rs2229742	G	C	0.025	0.003	-0.019	0.012
25(OH)D_UKBB	FinnGen_CVD	rs2248551	G	A	0.023	0.003	-0.023	0.009
25(OH)D_UKBB	FinnGen_CVD	rs2346264	A	C	0.014	0.002	-0.008	0.011
25(OH)D_UKBB	FinnGen_CVD	rs2585442	C	G	-0.027	0.002	0.005	0.008
25(OH)D_UKBB	FinnGen_CVD	rs261291	T	C	0.028	0.002	-0.008	0.008
25(OH)D_UKBB	FinnGen_CVD	rs2710651	G	A	0.011	0.002	0.005	0.008
25(OH)D_UKBB	FinnGen_CVD	rs2725371	A	G	-0.012	0.002	0.026	0.009
25(OH)D_UKBB	FinnGen_CVD	rs2762943	T	G	-0.045	0.004	-0.016	0.013
25(OH)D_UKBB	FinnGen_CVD	rs28364331	A	G	-0.070	0.007	0.015	0.070
25(OH)D_UKBB	FinnGen_CVD	rs28367476	A	G	0.014	0.002	0.004	0.008
25(OH)D_UKBB	FinnGen_CVD	rs28374650	C	T	0.013	0.002	0.014	0.010
25(OH)D_UKBB	FinnGen_CVD	rs2847500	G	A	0.022	0.003	-0.015	0.011
25(OH)D_UKBB	FinnGen_CVD	rs28692966	G	A	-0.015	0.002	0.021	0.008
25(OH)D_UKBB	FinnGen_CVD	rs2952289	C	T	-0.018	0.002	-0.013	0.010

25(OH)D_UKBB	FinnGen_CVD	rs31612	T	C	0.015	0.003	-0.001	0.009
25(OH)D_UKBB	FinnGen_CVD	rs325384	C	T	0.014	0.002	0.007	0.008
25(OH)D_UKBB	FinnGen_CVD	rs34290760	C	G	0.039	0.006	-0.022	0.016
25(OH)D_UKBB	FinnGen_CVD	rs35057908	A	T	-0.016	0.002	-0.006	0.008
25(OH)D_UKBB	FinnGen_CVD	rs35408430	C	T	0.021	0.002	0.001	0.008
25(OH)D_UKBB	FinnGen_CVD	rs3814995	C	T	0.012	0.002	0.006	0.008
25(OH)D_UKBB	FinnGen_CVD	rs3849374	G	C	0.016	0.003	0.000	0.010
25(OH)D_UKBB	FinnGen_CVD	rs4121823	T	A	0.019	0.003	0.006	0.011
25(OH)D_UKBB	FinnGen_CVD	rs4327060	C	T	0.025	0.004	0.019	0.012
25(OH)D_UKBB	FinnGen_CVD	rs4364259	G	A	-0.016	0.003	-0.006	0.009
25(OH)D_UKBB	FinnGen_CVD	rs4418728	G	T	-0.011	0.002	-0.003	0.008
25(OH)D_UKBB	FinnGen_CVD	rs4575545	G	A	0.016	0.002	0.012	0.008
25(OH)D_UKBB	FinnGen_CVD	rs4616820	C	T	0.012	0.002	0.008	0.008
25(OH)D_UKBB	FinnGen_CVD	rs484195	A	G	0.016	0.002	-0.007	0.008
25(OH)D_UKBB	FinnGen_CVD	rs541041	G	A	0.016	0.003	0.000	0.010
25(OH)D_UKBB	FinnGen_CVD	rs55829990	T	C	0.019	0.002	0.006	0.008
25(OH)D_UKBB	FinnGen_CVD	rs590215	C	T	0.013	0.002	-0.011	0.010
25(OH)D_UKBB	FinnGen_CVD	rs6003456	T	A	0.013	0.002	-0.003	0.010
25(OH)D_UKBB	FinnGen_CVD	rs6123359	A	G	-0.025	0.003	-0.007	0.012
25(OH)D_UKBB	FinnGen_CVD	rs613808	A	G	-0.016	0.002	0.015	0.008
25(OH)D_UKBB	FinnGen_CVD	rs61816761	G	A	-0.077	0.010	-0.006	0.075
25(OH)D_UKBB	FinnGen_CVD	rs61883501	A	C	-0.033	0.006	0.000	0.030
25(OH)D_UKBB	FinnGen_CVD	rs61891388	T	G	-0.013	0.002	0.004	0.008
25(OH)D_UKBB	FinnGen_CVD	rs62007299	G	A	0.013	0.002	-0.011	0.008
25(OH)D_UKBB	FinnGen_CVD	rs6671730	G	A	0.015	0.002	0.010	0.008
25(OH)D_UKBB	FinnGen_CVD	rs6672758	C	T	-0.017	0.003	-0.004	0.009
25(OH)D_UKBB	FinnGen_CVD	rs6782190	G	A	0.018	0.002	0.011	0.009
25(OH)D_UKBB	FinnGen_CVD	rs705117	C	T	-0.032	0.003	-0.002	0.012
25(OH)D_UKBB	FinnGen_CVD	rs7149014	T	C	0.013	0.002	0.001	0.008
25(OH)D_UKBB	FinnGen_CVD	rs727857	G	A	0.014	0.002	-0.011	0.008
25(OH)D_UKBB	FinnGen_CVD	rs72834856	T	G	0.025	0.004	0.002	0.016

25(OH)D_UKBB	FinnGen_CVD	rs72997623	C	A	-0.028	0.004	0.002	0.014
25(OH)D_UKBB	FinnGen_CVD	rs73413596	T	C	-0.022	0.004	0.032	0.017
25(OH)D_UKBB	FinnGen_CVD	rs7412	C	T	-0.030	0.004	0.039	0.017
25(OH)D_UKBB	FinnGen_CVD	rs7522116	C	T	0.013	0.002	-0.007	0.008
25(OH)D_UKBB	FinnGen_CVD	rs7528419	A	G	-0.020	0.002	0.019	0.009
25(OH)D_UKBB	FinnGen_CVD	rs7569755	G	A	-0.014	0.002	-0.017	0.009
25(OH)D_UKBB	FinnGen_CVD	rs75741381	C	G	0.016	0.003	-0.007	0.011
25(OH)D_UKBB	FinnGen_CVD	rs7604788	C	T	-0.036	0.006	-0.012	0.016
25(OH)D_UKBB	FinnGen_CVD	rs76798800	G	T	0.017	0.002	-0.013	0.009
25(OH)D_UKBB	FinnGen_CVD	rs77532868	C	T	-0.027	0.004	0.004	0.022
25(OH)D_UKBB	FinnGen_CVD	rs77924615	G	A	0.017	0.003	0.029	0.009
25(OH)D_UKBB	FinnGen_CVD	rs78151190	A	C	0.017	0.003	-0.015	0.013
25(OH)D_UKBB	FinnGen_CVD	rs78168201	C	T	-0.066	0.009	-0.048	0.058
25(OH)D_UKBB	FinnGen_CVD	rs78649910	T	A	0.021	0.003	-0.006	0.012
25(OH)D_UKBB	FinnGen_CVD	rs8018720	G	C	0.038	0.003	0.000	0.011
25(OH)D_UKBB	FinnGen_CVD	rs804281	A	G	-0.015	0.002	0.015	0.011
25(OH)D_UKBB	FinnGen_CVD	rs8091117	C	A	0.026	0.004	0.008	0.014
25(OH)D_UKBB	FinnGen_CVD	rs8113404	C	T	-0.013	0.002	0.007	0.008
25(OH)D_UKBB	FinnGen_CVD	rs867772	A	G	0.014	0.002	0.003	0.009
25(OH)D_UKBB	FinnGen_CVD	rs9476310	C	T	-0.012	0.002	0.003	0.008
25(OH)D_UKBB	FinnGen_CVD	rs9490317	T	C	-0.011	0.002	-0.009	0.008
25(OH)D_UKBB	FinnGen_CVD	rs964184	G	C	-0.035	0.003	0.042	0.011
25(OH)D_UKBB	FinnGen_CVD	rs9861009	T	C	-0.014	0.002	-0.002	0.009
25(OH)D_UKBB	Longevity_90%	rs6671730	G	A	0.015	0.002	-0.056	0.022
25(OH)D_UKBB	Longevity_90%	rs35408430	C	T	0.021	0.002	0.007	0.020
25(OH)D_UKBB	Longevity_90%	rs7522116	C	T	0.013	0.002	0.006	0.020
25(OH)D_UKBB	Longevity_90%	rs11591147	T	G	0.045	0.007	0.150	0.110
25(OH)D_UKBB	Longevity_90%	rs2131925	G	T	0.023	0.002	-0.047	0.020
25(OH)D_UKBB	Longevity_90%	rs7528419	G	A	0.020	0.002	0.033	0.023
25(OH)D_UKBB	Longevity_90%	rs140371183	G	A	0.063	0.007	0.061	0.082
25(OH)D_UKBB	Longevity_90%	rs12123821	T	C	0.074	0.005	0.076	0.056

25(OH)D_UKBB	Longevity_90%	rs1933064	A	G	0.015	0.002	-0.009	0.021
25(OH)D_UKBB	Longevity_90%	rs10908419	G	A	0.012	0.002	-0.018	0.019
25(OH)D_UKBB	Longevity_90%	rs76798800	G	T	0.017	0.002	-0.003	0.022
25(OH)D_UKBB	Longevity_90%	rs11264322	G	A	0.013	0.002	0.009	0.020
25(OH)D_UKBB	Longevity_90%	rs10908465	T	C	0.020	0.002	-0.072	0.022
25(OH)D_UKBB	Longevity_90%	rs867772	A	G	0.014	0.002	0.022	0.021
25(OH)D_UKBB	Longevity_90%	rs6672758	T	C	0.017	0.003	0.007	0.024
25(OH)D_UKBB	Longevity_90%	rs7604788	T	C	0.036	0.006	0.075	0.054
25(OH)D_UKBB	Longevity_90%	rs541041	G	A	0.016	0.003	0.047	0.025
25(OH)D_UKBB	Longevity_90%	rs1260326	C	T	0.021	0.002	0.029	0.020
25(OH)D_UKBB	Longevity_90%	rs11127186	C	T	0.011	0.002	0.012	0.021
25(OH)D_UKBB	Longevity_90%	rs727857	G	A	0.014	0.002	-0.017	0.020
25(OH)D_UKBB	Longevity_90%	rs2710651	G	A	0.011	0.002	0.021	0.019
25(OH)D_UKBB	Longevity_90%	rs3849374	G	C	0.016	0.003	0.029	0.024
25(OH)D_UKBB	Longevity_90%	rs7569755	A	G	0.014	0.002	0.010	0.021
25(OH)D_UKBB	Longevity_90%	rs1047891	C	A	0.015	0.002	-0.009	0.022
25(OH)D_UKBB	Longevity_90%	rs2012736	C	A	0.048	0.004	-0.032	0.043
25(OH)D_UKBB	Longevity_90%	rs13060130	C	T	0.017	0.003	-0.035	0.027
25(OH)D_UKBB	Longevity_90%	rs6782190	G	A	0.018	0.002	0.013	0.020
25(OH)D_UKBB	Longevity_90%	rs9861009	C	T	0.014	0.002	0.034	0.022
25(OH)D_UKBB	Longevity_90%	rs78649910	T	A	0.021	0.003	0.044	0.035
25(OH)D_UKBB	Longevity_90%	rs4364259	A	G	0.016	0.003	-0.021	0.029
25(OH)D_UKBB	Longevity_90%	rs4616820	C	T	0.012	0.002	0.003	0.019
25(OH)D_UKBB	Longevity_90%	rs13104260	A	G	0.020	0.002	-0.031	0.022
25(OH)D_UKBB	Longevity_90%	rs705117	T	C	0.032	0.003	0.017	0.027
25(OH)D_UKBB	Longevity_90%	rs1352846	A	G	0.196	0.002	-0.013	0.021
25(OH)D_UKBB	Longevity_90%	rs11732896	G	A	0.016	0.002	0.029	0.021
25(OH)D_UKBB	Longevity_90%	rs28364331	G	A	0.070	0.007	0.049	0.087
25(OH)D_UKBB	Longevity_90%	rs1229984	T	C	0.047	0.006	0.122	0.053
25(OH)D_UKBB	Longevity_90%	rs10070734	C	T	0.013	0.002	0.032	0.022
25(OH)D_UKBB	Longevity_90%	rs31612	T	C	0.015	0.003	-0.014	0.025

25(OH)D_UKBB	Longevity_90%	rs72834856	T	G	0.025	0.004	0.057	0.041
25(OH)D_UKBB	Longevity_90%	rs78151190	A	C	0.017	0.003	0.011	0.030
25(OH)D_UKBB	Longevity_90%	rs9476310	T	C	0.012	0.002	-0.046	0.020
25(OH)D_UKBB	Longevity_90%	rs9490317	C	T	0.011	0.002	0.002	0.019
25(OH)D_UKBB	Longevity_90%	rs2248551	G	A	0.023	0.003	0.015	0.027
25(OH)D_UKBB	Longevity_90%	rs10085881	T	C	0.015	0.002	0.012	0.022
25(OH)D_UKBB	Longevity_90%	rs7784802	T	A	0.014	0.002	-0.005	0.021
25(OH)D_UKBB	Longevity_90%	rs75741381	C	G	0.016	0.003	-0.012	0.028
25(OH)D_UKBB	Longevity_90%	rs6966728	C	T	0.012	0.002	0.009	0.019
25(OH)D_UKBB	Longevity_90%	rs2346264	A	C	0.014	0.002	0.008	0.023
25(OH)D_UKBB	Longevity_90%	rs34290760	C	G	0.039	0.006	-0.045	0.062
25(OH)D_UKBB	Longevity_90%	rs804281	G	A	0.015	0.002	0.021	0.020
25(OH)D_UKBB	Longevity_90%	rs28692966	A	G	0.015	0.002	-0.028	0.022
25(OH)D_UKBB	Longevity_90%	rs2725371	G	A	0.012	0.002	0.002	0.021
25(OH)D_UKBB	Longevity_90%	rs4738684	G	A	0.012	0.002	0.020	0.020
25(OH)D_UKBB	Longevity_90%	rs12056768	T	G	0.025	0.002	-0.007	0.020
25(OH)D_UKBB	Longevity_90%	rs13284054	C	T	0.018	0.003	-0.028	0.030
25(OH)D_UKBB	Longevity_90%	rs10887718	C	T	0.011	0.002	-0.003	0.019
25(OH)D_UKBB	Longevity_90%	rs77532868	T	C	0.027	0.004	0.005	0.050
25(OH)D_UKBB	Longevity_90%	rs3925446	A	G	0.016	0.002	0.067	0.024
25(OH)D_UKBB	Longevity_90%	rs4418728	T	G	0.011	0.002	0.037	0.019
25(OH)D_UKBB	Longevity_90%	rs61883501	C	A	0.033	0.006	-0.120	0.071
25(OH)D_UKBB	Longevity_90%	rs116970203	G	A	0.428	0.006	-0.052	0.078
25(OH)D_UKBB	Longevity_90%	rs12794714	G	A	0.113	0.002	-0.015	0.019
25(OH)D_UKBB	Longevity_90%	rs61891388	G	T	0.013	0.002	-0.035	0.019
25(OH)D_UKBB	Longevity_90%	rs1660839	A	G	0.015	0.002	0.003	0.022
25(OH)D_UKBB	Longevity_90%	rs12803256	G	A	0.067	0.004	-0.034	0.021
25(OH)D_UKBB	Longevity_90%	rs12798050	T	C	0.048	0.004	0.007	0.025
25(OH)D_UKBB	Longevity_90%	rs72997623	A	C	0.028	0.004	-0.028	0.032
25(OH)D_UKBB	Longevity_90%	rs1149605	C	T	0.023	0.003	0.001	0.026
25(OH)D_UKBB	Longevity_90%	rs964184	C	G	0.035	0.003	0.055	0.028

25(OH)D_UKBB	Longevity_90%	rs613808	G	A	0.016	0.002	0.052	0.021
25(OH)D_UKBB	Longevity_90%	rs2847500	G	A	0.022	0.003	0.016	0.032
25(OH)D_UKBB	Longevity_90%	rs12317268	A	G	0.021	0.003	-0.016	0.026
25(OH)D_UKBB	Longevity_90%	rs11182428	T	C	0.013	0.002	0.018	0.019
25(OH)D_UKBB	Longevity_90%	rs1038165	T	C	0.012	0.002	-0.016	0.019
25(OH)D_UKBB	Longevity_90%	rs10859995	T	C	0.044	0.002	0.003	0.019
25(OH)D_UKBB	Longevity_90%	rs11108368	G	A	0.014	0.002	-0.019	0.020
25(OH)D_UKBB	Longevity_90%	rs12372115	G	T	0.022	0.004	0.031	0.041
25(OH)D_UKBB	Longevity_90%	rs73413596	C	T	0.022	0.004	0.016	0.038
25(OH)D_UKBB	Longevity_90%	rs7149014	T	C	0.013	0.002	0.026	0.020
25(OH)D_UKBB	Longevity_90%	rs8018720	G	C	0.038	0.003	-0.032	0.025
25(OH)D_UKBB	Longevity_90%	rs12881545	C	G	0.012	0.002	0.019	0.021
25(OH)D_UKBB	Longevity_90%	rs261291	T	C	0.028	0.002	-0.038	0.021
25(OH)D_UKBB	Longevity_90%	rs1800588	C	T	0.034	0.002	0.011	0.024
25(OH)D_UKBB	Longevity_90%	rs55829990	T	C	0.019	0.002	0.022	0.020
25(OH)D_UKBB	Longevity_90%	rs62007299	G	A	0.013	0.002	0.033	0.021
25(OH)D_UKBB	Longevity_90%	rs325384	C	T	0.014	0.002	-0.011	0.022
25(OH)D_UKBB	Longevity_90%	rs77924615	G	A	0.017	0.003	-0.038	0.026
25(OH)D_UKBB	Longevity_90%	rs17231506	C	T	0.014	0.002	-0.038	0.021
25(OH)D_UKBB	Longevity_90%	rs11076175	G	A	0.018	0.003	-0.016	0.026
25(OH)D_UKBB	Longevity_90%	rs4327060	C	T	0.025	0.004	0.068	0.045
25(OH)D_UKBB	Longevity_90%	rs4575545	G	A	0.016	0.002	0.025	0.021
25(OH)D_UKBB	Longevity_90%	rs11542462	G	A	0.024	0.003	0.041	0.035
25(OH)D_UKBB	Longevity_90%	rs10454087	C	T	0.014	0.002	0.013	0.022
25(OH)D_UKBB	Longevity_90%	rs2952289	T	C	0.018	0.002	-0.018	0.024
25(OH)D_UKBB	Longevity_90%	rs8091117	C	A	0.026	0.004	-0.002	0.037
25(OH)D_UKBB	Longevity_90%	rs4121823	T	A	0.019	0.003	-0.018	0.027
25(OH)D_UKBB	Longevity_90%	rs590215	C	T	0.013	0.002	0.026	0.022
25(OH)D_UKBB	Longevity_90%	rs2037511	A	G	0.018	0.003	0.009	0.026
25(OH)D_UKBB	Longevity_90%	rs142158911	A	G	0.025	0.003	0.082	0.032
25(OH)D_UKBB	Longevity_90%	rs3814995	C	T	0.012	0.002	0.011	0.025

25(OH)D_UKBB	Longevity_90%	rs7412	T	C	0.030	0.004	0.245	0.037
25(OH)D_UKBB	Longevity_90%	rs484195	A	G	0.016	0.002	0.095	0.022
25(OH)D_UKBB	Longevity_90%	rs212100	T	C	0.066	0.003	-0.022	0.026
25(OH)D_UKBB	Longevity_90%	rs10426	A	G	0.025	0.002	0.048	0.024
25(OH)D_UKBB	Longevity_90%	rs8113404	T	C	0.013	0.002	-0.005	0.022
25(OH)D_UKBB	Longevity_90%	rs11606	G	C	0.012	0.002	-0.006	0.023
25(OH)D_UKBB	Longevity_90%	rs2207132	G	A	0.035	0.006	0.197	0.072
25(OH)D_UKBB	Longevity_90%	rs6123359	G	A	0.025	0.003	0.016	0.032
25(OH)D_UKBB	Longevity_90%	rs17216707	T	C	0.032	0.003	0.012	0.026
25(OH)D_UKBB	Longevity_90%	rs2585442	G	C	0.027	0.002	0.017	0.024
25(OH)D_UKBB	Longevity_90%	rs2762943	G	T	0.045	0.004	-0.039	0.056
25(OH)D_UKBB	Longevity_90%	rs2229742	G	C	0.025	0.003	0.026	0.034
25(OH)D_UKBB	Longevity_90%	rs6003456	T	A	0.013	0.002	-0.051	0.023
25(OH)D_UKBB	Longevity_90%	rs2074735	C	G	0.028	0.004	-0.013	0.039
25(OH)D_UKBB	Longevity_90%_UKBB	rs10070734	T	C	-0.013	0.002	-0.077	0.029
25(OH)D_UKBB	Longevity_90%_UKBB	rs10085881	T	C	0.015	0.002	0.019	0.029
25(OH)D_UKBB	Longevity_90%_UKBB	rs1038165	C	T	-0.012	0.002	-0.015	0.027
25(OH)D_UKBB	Longevity_90%_UKBB	rs10426	G	A	-0.025	0.002	0.043	0.032
25(OH)D_UKBB	Longevity_90%_UKBB	rs10454087	C	T	0.014	0.002	0.073	0.029
25(OH)D_UKBB	Longevity_90%_UKBB	rs1047891	C	A	0.015	0.002	0.030	0.028
25(OH)D_UKBB	Longevity_90%_UKBB	rs10859995	T	C	0.044	0.002	0.044	0.026
25(OH)D_UKBB	Longevity_90%_UKBB	rs10887718	C	T	0.011	0.002	-0.003	0.026
25(OH)D_UKBB	Longevity_90%_UKBB	rs10908419	G	A	0.012	0.002	0.002	0.026
25(OH)D_UKBB	Longevity_90%_UKBB	rs10908465	C	T	-0.02	0.002	0.033	0.029
25(OH)D_UKBB	Longevity_90%_UKBB	rs11076175	A	G	-0.018	0.003	0.037	0.034
25(OH)D_UKBB	Longevity_90%_UKBB	rs11108368	A	G	-0.014	0.002	0.000	0.027
25(OH)D_UKBB	Longevity_90%_UKBB	rs11127186	T	C	-0.011	0.002	0.009	0.026
25(OH)D_UKBB	Longevity_90%_UKBB	rs11182428	T	C	0.013	0.002	-0.017	0.026
25(OH)D_UKBB	Longevity_90%_UKBB	rs11264322	G	A	0.013	0.002	0.026	0.026
25(OH)D_UKBB	Longevity_90%_UKBB	rs1149605	T	C	-0.023	0.003	0.064	0.035
25(OH)D_UKBB	Longevity_90%_UKBB	rs11542462	G	A	0.024	0.003	-0.026	0.038

25(OH)D_UKBB	Longevity_90%_UKBB	rs115621755	C	T	0.012	0.002	0.009	0.028
25(OH)D_UKBB	Longevity_90%_UKBB	rs11591147	G	T	-0.045	0.007	-0.208	0.100
25(OH)D_UKBB	Longevity_90%_UKBB	rs11606	C	G	-0.012	0.002	0.009	0.027
25(OH)D_UKBB	Longevity_90%_UKBB	rs116970203	G	A	0.428	0.006	-0.116	0.080
25(OH)D_UKBB	Longevity_90%_UKBB	rs11732896	G	A	0.016	0.002	0.024	0.028
25(OH)D_UKBB	Longevity_90%_UKBB	rs117576073	G	T	0.215	0.009	-0.100	0.117
25(OH)D_UKBB	Longevity_90%_UKBB	rs12056768	T	G	0.025	0.002	0.001	0.027
25(OH)D_UKBB	Longevity_90%_UKBB	rs12123821	C	T	-0.074	0.005	-0.030	0.061
25(OH)D_UKBB	Longevity_90%_UKBB	rs1229984	T	C	0.047	0.006	0.115	0.085
25(OH)D_UKBB	Longevity_90%_UKBB	rs12317268	A	G	0.021	0.003	-0.076	0.037
25(OH)D_UKBB	Longevity_90%_UKBB	rs12372115	G	T	0.022	0.004	0.055	0.051
25(OH)D_UKBB	Longevity_90%_UKBB	rs1260326	T	C	-0.021	0.002	0.003	0.027
25(OH)D_UKBB	Longevity_90%_UKBB	rs12794714	G	A	0.113	0.002	0.015	0.026
25(OH)D_UKBB	Longevity_90%_UKBB	rs12803256	A	G	-0.067	0.004	0.016	0.031
25(OH)D_UKBB	Longevity_90%_UKBB	rs12881545	G	C	-0.012	0.002	0.034	0.028
25(OH)D_UKBB	Longevity_90%_UKBB	rs13060130	C	T	0.017	0.003	0.001	0.038
25(OH)D_UKBB	Longevity_90%_UKBB	rs13104260	G	A	-0.02	0.002	0.018	0.030
25(OH)D_UKBB	Longevity_90%_UKBB	rs13284054	T	C	-0.018	0.003	-0.052	0.040
25(OH)D_UKBB	Longevity_90%_UKBB	rs1352846	A	G	0.196	0.002	-0.053	0.029
25(OH)D_UKBB	Longevity_90%_UKBB	rs140371183	A	G	-0.063	0.007	0.026	0.072
25(OH)D_UKBB	Longevity_90%_UKBB	rs142158911	G	A	-0.025	0.003	-0.088	0.041
25(OH)D_UKBB	Longevity_90%_UKBB	rs1660839	G	A	-0.015	0.002	-0.033	0.030
25(OH)D_UKBB	Longevity_90%_UKBB	rs17216707	T	C	0.032	0.003	-0.041	0.034
25(OH)D_UKBB	Longevity_90%_UKBB	rs17231506	C	T	0.014	0.002	-0.032	0.028
25(OH)D_UKBB	Longevity_90%_UKBB	rs1800588	C	T	0.034	0.002	0.020	0.032
25(OH)D_UKBB	Longevity_90%_UKBB	rs187429064	A	G	-0.068	0.009	-0.240	0.119
25(OH)D_UKBB	Longevity_90%_UKBB	rs1933064	G	A	-0.015	0.002	0.018	0.026
25(OH)D_UKBB	Longevity_90%_UKBB	rs2037511	G	A	-0.018	0.003	0.058	0.035
25(OH)D_UKBB	Longevity_90%_UKBB	rs2074735	G	C	-0.028	0.004	-0.050	0.054
25(OH)D_UKBB	Longevity_90%_UKBB	rs212100	T	C	0.066	0.003	0.013	0.035
25(OH)D_UKBB	Longevity_90%_UKBB	rs2131925	G	T	0.023	0.002	-0.021	0.027

25(OH)D_UKBB	Longevity_90%_UKBB	rs2207132	G	A	0.035	0.006	0.079	0.073
25(OH)D_UKBB	Longevity_90%_UKBB	rs2229742	G	C	0.025	0.003	0.062	0.043
25(OH)D_UKBB	Longevity_90%_UKBB	rs2248551	G	A	0.023	0.003	0.027	0.035
25(OH)D_UKBB	Longevity_90%_UKBB	rs2346264	A	C	0.014	0.002	-0.044	0.032
25(OH)D_UKBB	Longevity_90%_UKBB	rs2585442	C	G	-0.027	0.002	0.015	0.031
25(OH)D_UKBB	Longevity_90%_UKBB	rs261291	T	C	0.028	0.002	-0.019	0.027
25(OH)D_UKBB	Longevity_90%_UKBB	rs2710651	G	A	0.011	0.002	-0.011	0.026
25(OH)D_UKBB	Longevity_90%_UKBB	rs2725371	A	G	-0.012	0.002	-0.055	0.028
25(OH)D_UKBB	Longevity_90%_UKBB	rs2762943	T	G	-0.045	0.004	0.046	0.049
25(OH)D_UKBB	Longevity_90%_UKBB	rs28364331	A	G	-0.07	0.007	0.091	0.098
25(OH)D_UKBB	Longevity_90%_UKBB	rs28374650	C	T	0.013	0.002	-0.059	0.035
25(OH)D_UKBB	Longevity_90%_UKBB	rs2847500	G	A	0.022	0.003	0.025	0.040
25(OH)D_UKBB	Longevity_90%_UKBB	rs28692966	G	A	-0.015	0.002	-0.007	0.030
25(OH)D_UKBB	Longevity_90%_UKBB	rs2952289	C	T	-0.018	0.002	-0.050	0.032
25(OH)D_UKBB	Longevity_90%_UKBB	rs31612	T	C	0.015	0.003	-0.009	0.034
25(OH)D_UKBB	Longevity_90%_UKBB	rs325384	C	T	0.014	0.002	-0.043	0.029
25(OH)D_UKBB	Longevity_90%_UKBB	rs34290760	C	G	0.039	0.006	-0.083	0.077
25(OH)D_UKBB	Longevity_90%_UKBB	rs35057908	A	T	-0.016	0.002	0.048	0.027
25(OH)D_UKBB	Longevity_90%_UKBB	rs35408430	C	T	0.021	0.002	0.020	0.028
25(OH)D_UKBB	Longevity_90%_UKBB	rs3814995	C	T	0.012	0.002	0.026	0.028
25(OH)D_UKBB	Longevity_90%_UKBB	rs3849374	G	C	0.016	0.003	-0.030	0.034
25(OH)D_UKBB	Longevity_90%_UKBB	rs3925446	G	A	-0.016	0.002	0.002	0.032
25(OH)D_UKBB	Longevity_90%_UKBB	rs4121823	T	A	0.019	0.003	-0.013	0.036
25(OH)D_UKBB	Longevity_90%_UKBB	rs4327060	C	T	0.025	0.004	-0.001	0.058
25(OH)D_UKBB	Longevity_90%_UKBB	rs4364259	G	A	-0.016	0.003	-0.023	0.033
25(OH)D_UKBB	Longevity_90%_UKBB	rs4418728	G	T	-0.011	0.002	0.007	0.026
25(OH)D_UKBB	Longevity_90%_UKBB	rs4575545	G	A	0.016	0.002	0.001	0.028
25(OH)D_UKBB	Longevity_90%_UKBB	rs4616820	C	T	0.012	0.002	0.001	0.026
25(OH)D_UKBB	Longevity_90%_UKBB	rs4738684	A	G	-0.012	0.002	-0.004	0.028
25(OH)D_UKBB	Longevity_90%_UKBB	rs484195	A	G	0.016	0.002	0.173	0.028
25(OH)D_UKBB	Longevity_90%_UKBB	rs541041	G	A	0.016	0.003	0.041	0.034

25(OH)D_UKBB	Longevity_90%_UKBB	rs55829990	T	C	0.019	0.002	0.044	0.028
25(OH)D_UKBB	Longevity_90%_UKBB	rs590215	C	T	0.013	0.002	0.064	0.030
25(OH)D_UKBB	Longevity_90%_UKBB	rs6003456	T	A	0.013	0.002	0.013	0.031
25(OH)D_UKBB	Longevity_90%_UKBB	rs6123359	A	G	-0.025	0.003	0.025	0.043
25(OH)D_UKBB	Longevity_90%_UKBB	rs613808	A	G	-0.016	0.002	0.036	0.029
25(OH)D_UKBB	Longevity_90%_UKBB	rs61816761	G	A	-0.077	0.01	-0.047	0.091
25(OH)D_UKBB	Longevity_90%_UKBB	rs61883501	A	C	-0.033	0.006	0.034	0.074
25(OH)D_UKBB	Longevity_90%_UKBB	rs61891388	T	G	-0.013	0.002	-0.082	0.026
25(OH)D_UKBB	Longevity_90%_UKBB	rs62007299	G	A	0.013	0.002	0.084	0.029
25(OH)D_UKBB	Longevity_90%_UKBB	rs6671730	G	A	0.015	0.002	-0.075	0.026
25(OH)D_UKBB	Longevity_90%_UKBB	rs6672758	C	T	-0.017	0.003	-0.028	0.033
25(OH)D_UKBB	Longevity_90%_UKBB	rs6782190	G	A	0.018	0.002	0.022	0.027
25(OH)D_UKBB	Longevity_90%_UKBB	rs6966728	C	T	0.012	0.002	0.022	0.026
25(OH)D_UKBB	Longevity_90%_UKBB	rs705117	C	T	-0.032	0.003	0.003	0.037
25(OH)D_UKBB	Longevity_90%_UKBB	rs7149014	T	C	0.013	0.002	-0.007	0.028
25(OH)D_UKBB	Longevity_90%_UKBB	rs727857	G	A	0.014	0.002	0.029	0.027
25(OH)D_UKBB	Longevity_90%_UKBB	rs72834856	T	G	0.025	0.004	0.067	0.050
25(OH)D_UKBB	Longevity_90%_UKBB	rs72997623	C	A	-0.028	0.004	-0.044	0.047
25(OH)D_UKBB	Longevity_90%_UKBB	rs73413596	T	C	-0.022	0.004	-0.034	0.050
25(OH)D_UKBB	Longevity_90%_UKBB	rs7412	C	T	-0.03	0.004	-0.247	0.048
25(OH)D_UKBB	Longevity_90%_UKBB	rs7522116	C	T	0.013	0.002	-0.079	0.026
25(OH)D_UKBB	Longevity_90%_UKBB	rs7528419	A	G	-0.02	0.002	-0.101	0.031
25(OH)D_UKBB	Longevity_90%_UKBB	rs7569755	G	A	-0.014	0.002	-0.022	0.029
25(OH)D_UKBB	Longevity_90%_UKBB	rs75741381	C	G	0.016	0.003	0.039	0.037
25(OH)D_UKBB	Longevity_90%_UKBB	rs7604788	C	T	-0.036	0.006	-0.043	0.073
25(OH)D_UKBB	Longevity_90%_UKBB	rs76798800	G	T	0.017	0.002	-0.043	0.030
25(OH)D_UKBB	Longevity_90%_UKBB	rs77532868	C	T	-0.027	0.004	-0.033	0.058
25(OH)D_UKBB	Longevity_90%_UKBB	rs7784802	A	T	-0.014	0.002	-0.053	0.027
25(OH)D_UKBB	Longevity_90%_UKBB	rs77924615	G	A	0.017	0.003	-0.020	0.033
25(OH)D_UKBB	Longevity_90%_UKBB	rs78151190	A	C	0.017	0.003	0.071	0.039
25(OH)D_UKBB	Longevity_90%_UKBB	rs78168201	C	T	-0.066	0.009	-0.041	0.101

25(OH)D_UKBB	Longevity_90%_UKBB	rs78649910	T	A	0.021	0.003	0.056	0.042
25(OH)D_UKBB	Longevity_90%_UKBB	rs8018720	G	C	0.038	0.003	-0.042	0.034
25(OH)D_UKBB	Longevity_90%_UKBB	rs804281	A	G	-0.015	0.002	0.026	0.027
25(OH)D_UKBB	Longevity_90%_UKBB	rs8091117	C	A	0.026	0.004	-0.094	0.053
25(OH)D_UKBB	Longevity_90%_UKBB	rs8113404	C	T	-0.013	0.002	-0.022	0.028
25(OH)D_UKBB	Longevity_90%_UKBB	rs867772	A	G	0.014	0.002	-0.018	0.028
25(OH)D_UKBB	Longevity_90%_UKBB	rs9476310	C	T	-0.012	0.002	-0.011	0.026
25(OH)D_UKBB	Longevity_90%_UKBB	rs9490317	T	C	-0.011	0.002	0.031	0.026
25(OH)D_UKBB	Longevity_90%_UKBB	rs964184	G	C	-0.035	0.003	-0.022	0.038
25(OH)D_UKBB	Longevity_90%_UKBB	rs9861009	T	C	-0.014	0.002	0.002	0.029

EA, effect allele; NEA, non-effect allele; SNP, single nucleotide polymorphism.
